# Exploring the Diversity of Plant-Associated Viruses and Related Viruses in Riverine Freshwater Samples Collected in Berlin, Germany

**DOI:** 10.3390/pathogens12121458

**Published:** 2023-12-15

**Authors:** Roland Zell, Marco Groth, Lukas Selinka, Hans-Christoph Selinka

**Affiliations:** 1 Section of Experimental Virology, Institute for Medical Microbiology, Jena University Hospital, Friedrich Schiller University, 07745 Jena, Germany; 2 CF Next Generation Sequencing, Leibniz Institute on Aging-Fritz Lipmann Institute, 07745 Jena, Germany; 3 Section II 1.4 Microbiological Risks, Department of Environmental Hygiene, German Environment Agency, 14195 Berlin, Germany

**Keywords:** riverine ecosystems, viromes, phylogenetic analysis, metagenomic, RNA viruses, plant viruses

## Abstract

Plant-infecting RNA viruses from 30 families and floating genera, as well as a great number of uncultured as yet-unclassified plant-associated viruses have been described. Even so, the plant RNA virosphere is still underexplored. RNA extracted from enriched virus particles of 50 L water samples from the Teltow Canal and the Havel River in Berlin, Germany, was sequenced using Illumina next-generation sequencing. Sequences were searched for plant viruses with BLAST and DIAMOND. Phylogenetic analyses were conducted with IQ-TREE 2. Altogether, 647 virus sequences greater than 1 kb were detected and further analyzed. These data revealed the presence of accepted and novel viruses related to *Albetovirus*, *Alphaflexiviridae*, *Aspiviridae*, *Bromoviridae*, *Endornaviridae*, *Partitiviridae*, *Potyviridae*, *Solemoviridae*, *Tombusviridae* and *Virgaviridae*. The vast majority of the sequences were novel and could not be taxonomically assigned. Several tombus- and endorna-like viruses make use of alternative translation tables that suggest unicellular green algae, ciliates, or diplomonades as their hosts. The identification of 27 albeto-like satellite viruses increases available sequence data five-fold. Sixteen new poty-like viruses align with other poty-like viruses in a link that combines the *Astroviridae* and *Potyviridae* families. Further, the identification of viruses with peptidase A6-like and peptidase A21-like capsid proteins suggests horizontal gene transfer in the evolution of these viruses.

## 1. Introduction

The availability of potent nucleic acid extraction methods, affordable deep sequencing techniques, and powerful bioinformatic data processing tools has facilitated the unbiased identification of tens of thousands of previously unknown viruses from substrates as diverse as tissue specimens of a wide variety of animals and plants, fungi, protists, fecal samples and environmental matrices such as water, sediment, and soil [1,2,3]. Many of these new viruses are, in fact, uncultured virus genomes (UViGs) without any information except nucleotide sequences and their source. However, recent progress has allowed the unveiling of the vast extent of the virosphere, and every new viral metagenomics (viromics) study continues to identify new viruses. All these studies have in common that virus-like sequences are detectable in abundance in all investigated substrates and often with only little similarity to known viruses. Sequence divergence and little information on protein function are presently the main obstacles to proper classification, even though the formal classification of viruses and the creation of new taxa on the basis of sequence data from uncultivated viruses is feasible and already practiced [4,5,6]. These data collected in recent years enabled a provisional reconstruction of the RNA virus evolution and a draft of a comprehensive hierarchical taxonomy of viruses [5,7,8].

Our view on the biological role of viruses changed. Decades ago, virus research focused mainly on disease-causing pathogens and culturable viruses, which emphasized a highly biased segment of the virosphere. Now, viruses are considered important elements of holobionts, microbial communities, and the phytobiome and are hypothesized to perform key functions in ecosystems, e.g., by regulating host abundance, influencing population composition, and modification of aquatic food webs and nutrient cycles [9,10,11,12,13,14,15]. Regarding the impact of viruses on the stability of ecosystems, plant viruses are attributed to a pivotal role as plants constitute about 80% of the biomass on Earth [16]. Whereas many crop plants suffer from virus diseases, recent viromics studies revealed numerous viruses associated with wild plants, indicating a non-pathogenic role for many plant viruses. It is assumed that evolution fostered mutual plant-virus relationships of the phytobiome in unmanaged habitats in contrast to agriculture [15,17,18]. Plant cells are characterized by an extracellular cell wall that abuts the cell membrane and impedes viral infection. Therefore, either complex interactions between viruses, their plant hosts, and vectors evolved to ensure successful virus entry or vertical persistent infections established [18]. Since many of the recently discovered plant viruses are as yet uncultured, details of their multiplication cycles, host range, vectors, or transmission routes are unknown. Even the natural composition of plant infecting viromes of many ecosystems is still obscure.

The majority of viromics studies investigated the marine virosphere, which is dominated by DNA phages, but less is known about viruses in freshwater and soil ecosystems; especially RNA viruses in these environments are underexplored [19,20,21,22,23,24,25,26]. However, the characterization of viral communities is the first step toward understanding the role of viruses in an ecosystem. In order to investigate the virome of two riverine freshwater bodies influenced by human activities, we sequenced RNA extracted from water samples of the Teltow Canal and Havel River in Berlin, Germany. The diversity of picorna-like and hepe-like viruses in these waterbodies has been published previously [27,28]. Here, we describe hundreds of novel viruses presumably associated with plant hosts or related to plant viruses. Only a few viruses were readily typeable and belong to the genus *Albetovirus* and the *Alphaflexiviridae*, *Bromoviridae*, *Solemoviridae*, *Tombusviridae*, and *Virgaviridae* families. The overwhelming amount of the viral sequences of both waterbodies is new and eludes incorporation in acknowledged lower-rank virus taxa.

## 2. Materials and Methods

### 2.1. Sample Collection and Virus Enrichment 

**Figure 1:**
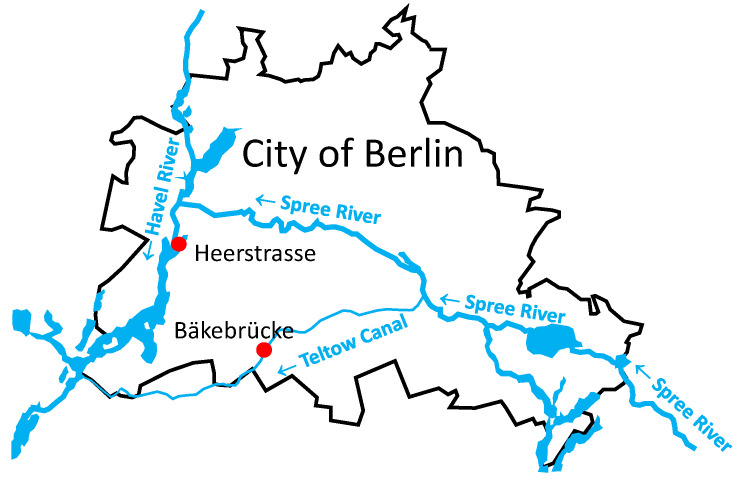
Map of the sampling locations. The municipal border of the City of Berlin is indicated by a black line. Two sampling sites, Heerstrasse at the Havel River and Bäkebrücke at the Teltow Canal, are indicated with red dots. Arrows indicate the flow directions of the Havel River, Spree River, and Teltow Canal.

Two 50 L freshwater samples were collected from (i) the Teltow Canal in Berlin, Bäkebrücke (coordinates 52°26′03″ N, 13°18′57″ E; see Figure 1), on 18 July 2017, sample ID MR233-17, and (ii) the Havel River in Berlin, Heerstrasse (coordinates 52°30′46″ N, 13°12′14″ E), on 28 June 2018, sample ID MR644-18. The procedure of virus enrichment by glass wool filtration followed the protocol of Wyn-Jones et al. [29]) and has been described previously [27]. Briefly, water samples were taken to the laboratory, aliquoted in 10 L subsamples, and mixed thoroughly by stirring for 20 min at 4 °C. The pH was adjusted to 3.5. Virus particles were adsorbed to negatively charged glass wool and eluted from the column with 3% beef extract/0.05 M glycine buffer pH 9.5. The eluate (180 mL) was neutralized (pH 7) and filtered (0.45 µm). Enriched virus particles were sedimented by ultracentrifugation (100,000× *g*, 2.5 h, 4 °C). The sediment was redissolved in 0.5 mL phosphate-buffered saline and stored at −80 °C.

### 2.2. RNA Preparation, Illumina Next-Generation Sequencing, Sequence Data Processing

RNA extraction was conducted using the QIAamp Viral RNA mini kit (Qiagen, Hilden, Germany) following the manufacturer’s protocol. The libraries were prepared as follows: Teltow Canal sample: 450 ng of total RNA was introduced into Illumina’s TruSeq stranded total RNA library preparation kit combined with Ribo-Zero Gold rRNA Removal Kit according to the manufacturer’s description; Havel River sample: 100 ng was introduced into Illumina’s TruSeq stranded mRNA library preparation kit. In order to address all RNA molecules (not only poly-adenylated RNA), the protocol was adapted as follows: RNA was precipitated using isopropanol and resolved in Fragment, Prime, Finish Mix (FPF). From this step on, the manufacturer’s protocol was followed (p20, step 12, TruSeq Stranded mRNA Sample Preparation Guide, Part # 15031047 Rev. E, Illumina).

The libraries were quantified and quality-checked using the 2100 Bioanalyzer in combination with a DNA 7500 assay and then sequenced on a HiSeq 2500 platform (rapid run mode, 2 × 150 bp paired-end). Sequence data were extracted in FastQ format using bcl2FastQ v2.19.1.403 (Illumina). Adapter sequences were trimmed using Cutadapt v1.8.3 [30] (parameters: -q 10 -m 30 -a AGATCGGAAGAGCACACGTCTGAACTCCAGTCA -A AGATCGGAAGAGCGTCGTGTAGGGAAAGAGTGT). Sample MR233-17 yielded 70,018,635 read pairs after adapter trimming and removal of duplicons, and sample MR644-18 had a total of 51,902,006 read pairs. De novo assembly was performed with two software tools, the clc_assembler v. 5.2.1 (parameters: -p fb ss 50 500) and metaSPAdes v3.15.3 [31] using standard parameters (-k auto). The clc_assembler yielded 537,529 contigs > 200 nt (sample MR233-17) and 162,082 contigs (sample MR644-18), respectively, whereas assembly with metaSPAdes resulted in 1,314,849 scaffolds for the Teltow Canal sample MR233-17 and 388,367 scaffolds for the Havel River sample MR644-18.

### 2.3. Sequence Data Analysis

All metaSPAdes scaffolds and clc contigs >200 nt were used to search a virus protein database compiled from all NCBI GenBank entries with Taxonomy ID 10239; search term “viruses[organism].” The search was executed with DIAMOND [32] and BLAST+ version 2.13.0 (https://ftp.ncbi.nlm.nih.gov/blast/executables/blast+/2.13.0/, accessed on 11 December 2023). For specific searches, scaffolds/contigs were searched with reference sequences using tBLASTx or BLASTn. In order to obtain the final full-length and partial genome sequences >1 kb, manual curation was performed by connecting overlapping contigs if appropriate. Protein domains were predicted using the NCBI web search tools BLASTp suite (https://blast.ncbi.nlm.nih.gov/Blast.cgi, accessed on 11 December 2023) and Pfam conserved domain database (CDD at https://www.ncbi.nlm.nih.gov/Structure/cdd/wrpsb.cgi, accessed on 11 December 2023). For phylogenetic analyses, sequences of this study and reference sequences downloaded from GenBank were aligned with ClustalW or Muscle implemented in Mega version X [33], manually adjusted, and utilized for maximum likelihood tree inference with IQ-TREE 2.1.3 for Windows [34]. The best-fit substitution models were estimated using the automatic model selection option (ModelFinder) of IQ-TREE. Branch support was assessed using standard non-parametric bootstrap (-b 1000) or the ultrafast bootstrap approximation UFBoot2 (-B 10000) implemented in IQ-TREE [35]. Mapping of reads to scaffolds/contigs was performed with HISAT2 [36]. HISAT2 was also used to map scaffolds/contigs to the curated sequences.

## 3. Results

### 3.1. Study Approach

Viruses from 30 RNA virus families and floating genera are known to infect plants. These are *Albetovirus*, *Alphaflexiviridae*, *Amalgaviridae*, *Aspiviridae*, *Aumaivirus*, *Benyviridae*, *Betaflexiviridae*, *Botourmiaviridae*, *Bromoviridae*, *Closteroviridae*, *Deltaflexiviridae*, *Endornaviridae*, *Fimoviridae*, *Gammaflexiviridae*, *Kitaviridae*, *Mayoviridae*, *Mymonaviridae*, *Papanivirus*, *Partitiviridae*, *Potyviridae*, *Rhabdoviridae*, *Secoviridae*, *Sedoreoviridae*, *Solemoviridae*, *Spinareoviridae*, *Tombusviridae*, *Tospoviridae*, *Tymoviridae*, *Virgaviridae*, and *Virtovirus*. For the identification of plant virus scaffolds and contigs, we used DIAMOND [32]. The DIAMOND search yielded numerous hits with e-values greater than 1 × 10 − 10 (Teltow Canal sample MR233-17: *n* = 66,371 metaSPAdes scaffolds and *n* = 41,408 CLC contigs; Havel River sample MR644-18: 16,922 metaSPAdes scaffolds and 8810 CLC contigs). Table 1 includes the sequences that were assigned by DIAMOND to plant-infecting viruses. In a second approach, we used BLASTn and tBLASTx to query the contig/scaffold banks with reference sequences representing each of the 30 plant-infecting virus families and genera. In the next step, all proposed contigs/scaffolds greater than 1 kb assigned to plant-infecting viruses (855 metaSPAdes scaffolds, 626 CLC contigs) were reassessed with BLAST in order to fix misassignments. Virus sequences smaller than 1 kb of the following taxa were identified but not investigated further: *Benyviridae* (*n* = 2), *Betaflexiviridae* (*n* = 9), *Closteroviridae* (*n* = 25), *Mymonaviridae* (*n* = 1), and *Tymoviridae* (*n* = 7). Furthermore, scaffolds greater than 1 kb assigned to *Amalgaviridae* (*n* = 2) and *Botourmiaviridae* (*n* = 142) clustered with sequences of mycoviruses rather than plant viruses of the respective families and were not further investigated here. Likewise, scaffolds assigned to the *Kitaviridae* (*n* = 1), *Secoviridae* (*n* = 5), and *Rhabdoviridae* (*n* = 1) were found to belong to non-plant-infecting viruses. These sequences (marked with asterisks in Table 1) and other obviously misassigned scaffolds/contigs were also discarded. Three contigs assigned to *Virtovirus* were revealed to be albeto-like viruses. No scaffolds/contigs of *Aumaivirus*, *Deltaflexiviridae*, *Fimoviridae*, *Gammaflexiviridae*, *Mayoviridae*, *Papanivirus*, *Sedoreoviridae*, *Spinareoviridae*, and *Tospoviridae* were identified by DIAMOND or BLAST. Our final set of plant viruses and plant virus-related viruses comprised 647 sequences greater than 1 kb (524 sequences from the Teltow Canal sample, 123 sequences from the Havel River sample; Supplementary Table S1).

In order to test the hypothesis of whether plant-infecting viruses of our Teltow Canal sample MR233-17 were also present in the Havel River sample and two Teltow Canal samples collected in 2016 [28], we used HISAT2 to search for scaffolds and contigs that map to the 524 sequences of MR233-17. In a reciprocal test, the datasets were also analyzed for sequences that map to the 123 Havel River sequences. The mapping results are presented in Table 2. These data demonstrate the presence of poty-like, solemo-like, tombus-like, and virga-like viruses in all samples of both waterbodies. Albeto-like, bromo-like, endorna-like, and partiti-like viruses were detected in both waterbodies but not in the samples from 2016. Alphaflexi-like sequences were present in Teltow Canal samples from 2016 and 2017.

### 3.2. Genetic Analysis of Viruses from Teltow Canal and Havel River

For further characterization, phylogenetic analyses were conducted with the RNA-dependent RNA polymerase (RdRp) encoding sequence, as the RdRp is the only universal and conserved protein of all RNA viruses. If no RdRp sequence was available, the helicase (Hel), movement protein (MP), and capsid protein (CP) encoding sequences were analyzed instead. It was made sure that the BLAST top hits with the highest scores were included in all sequence compilations.

#### 3.2.1. *Tombusviridae*

Viruses of the *Tombusviridae* and related tombus-like viruses constitute the majority of plant-infecting viruses in the Teltow Canal and Havel River. DIAMOND assigned 1710 metaSPAdes scaffolds/1174 CLC contigs of the Teltow Canal sample and 603 scaffolds/395 contigs of the Havel River to the *Tombusviridae*. This family comprises three subfamilies with 18 genera and 96 species (https://ictv.global/taxonomy, accessed on 11 December 2023). Members of the *Tombusviridae* except dianthoviruses have a monopartite, linear, positive-sense RNA genome (+ssRNA) ranging from 3.7 to 4.8 kb, whereas dianthoviruses have two +ssRNA genome segments with altogether 5.25 kb [37]. The RdRp gene is interrupted by an in-frame stop codon. A functional polymerase is generated either by a translational readthrough mechanism (*Procedovirinae*) or by ribosomal slippage, which leads to a −1 frameshift (*Regressovirinae, Calvusvirinae*). The capsid protein (CP), one or two movement proteins (MP), and a silencing suppressor protein are translated from subgenomic RNAs (sgRNAs). Other open reading frames (ORFs) may encode proteins. The majority of viruses of the *Tombusviridae* have a CP comprising three domains, i.e., the N-terminal RNA-interacting R domain, the central C domain with jellyroll fold, and the C-terminal protruding P domain. Only viruses of the genera *Alphanecrovirus*, *Betanecrovirus*, *Luteovirus, Machlomovirusus,* and *Panicovirus* lack the P domain. Such viruses exhibit a smoother appearance in electron-microscopical illustrations in comparison to viruses with the P domain. The icosahedral T = 3 capsid is composed of 180 subunits of CP. Umbraviruses have no CP.

Only tombus-like virus sequences greater than 1 kb were further analyzed. The sizes of metaSPAdes scaffolds ranged from 1 to 8.546 kb (mean 2.5411 kb), and those of the clc contigs from 1 to 8.184 kb (mean 2.3158 kb) for the Teltow Canal sample. For the Havel River sample, scaffold/contig lengths varied from 1 to 5.005 kb (mean 2.5959 kb) and 1 to 5.013 kb (mean 2.2991 kb), respectively. The final collection comprised 335 tombus-like viruses of the Teltow Canal and 106 tombus-like viruses of the Havel River. These viruses were named TC-Tombus-LV-1 to −335 and H-Tombus-LV-1 to −106, respectively.

**Figure 2:**
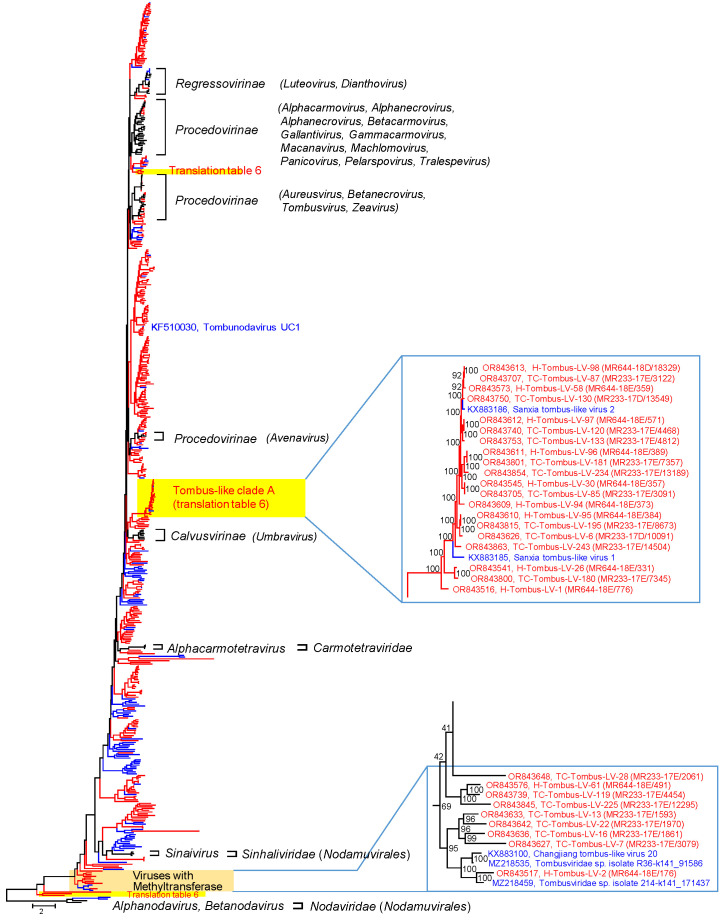
Phylogenetic analysis of tombus-like viruses. The dataset includes 518 RdRp sequences and comprises 357 tombus-like viruses of Teltow Canal and Havel River (red lines), 99 unclassified tombus-like viruses downloaded from GenBank (blue lines), 57 reference viruses of the *Tolivirales* and 5 reference viruses of the *Nodamuvirales* (black lines). Amino acid sequences were aligned with MEGA and used for tree inference with IQTREE 2 (optimal substitution model: VT+F+R10; 10,000 ultrafast replications). The tree was arbitrarily rooted with nodaviruses. Square brackets indicate reference viruses. Viruses that suppress termination codons are highlighted with yellow boxes. The scale bar indicates substitutions per site. Numbers at nodes of split images present bootstrap support. Details of the tree are presented in Figure S1.

For a phylogenetic analysis of the RdRp, 357 virus sequences of this study with suited polymerase gene sequence (282 TC-Tombus-LVs, 75 H-Tombus-LVs) plus 62 reference sequences of classified viruses and 99 sequences of unassigned tombus-like viruses retrieved from GenBank were aligned and used for tree inference (Figure 2 and Figure S1). The RdRp phylotree, which was arbitrarily rooted with nodavirus sequences, reveals monophyletic clades of *Regressovirinae* and *Calvusvirinae* and three branches with sequences of the *Procedovirinae*. In addition, numerous clades of unassigned viruses from Teltow Canal, Havel River and other studies suggest the presence of many yet undescribed species and genera of the *Tombusviridae*. These clades complicate a clear distinction between the families *Tombusviridae*, *Carmotetraviridae,* and *Sinhaliviridae* on the basis of their RdRp sequences. Due to long maximum likelihood distances, the exact position of some clades in this tree is unreliable, as indicated by low bootstrap support. In particular, branching close to the root appears to be inconsistent as RdRp sequences of this part of the tree show a mix of RdRp types 1 and 3, which corresponds to various conserved protein domain families (cd families), i.e., cd01699, cd23173, cd23174, cd23179, and cd23242.

Only 29 tombus-like viruses of our study cluster with viruses of existing subfamilies of the *Tombusviridae* and are candidates of this family (*Procedovirinae*: *n* = 25, *Regressovirinae*: *n* = 4, see genome layouts compiled in Figure S8A,B). The remaining 306 tombus-like sequences of Teltow Canal and Havel River could not be assigned to one of the acknowledged genera of the *Tombusviridae* and *Carmotetraviridae* and likely represent novel taxa of the *Tolivirales*.

Several findings are of interest:

(i) A group of 22 viruses (tombus-like clade A in Figure 2) but also a few other viruses use an alternative translation table that suppresses the UAG and UAA termination codons (translation table 6; https://www.bioinformatics.org/JaMBW/2/3/TranslationTables.html, accessed on 11 December 2023). The genome layout of tombus-like clade A viruses comprises three conserved ORFs (see Figure S8C). These are ORF 1, which encodes a hypothetical protein without similarity to known proteins, and two ORFs coding for the catalytic core domain of the RdRp and the CP. In addition, one to three ORFs with lengths up to 274 amino acids are detectable. However, these are not conserved, and their significance is unclear. A functional replicase is likely expressed by sgRNA transcription [38]. As no known frameshift signal was detectable, a ribosomal slippage mechanism is less likely. In contrast to the tombus-like clade A viruses, H-Tombus-LV-30, -44, -46 also use translation table 6 but cluster close to the root of the tree (Figure S1). Only partial genomes are available of these viruses. The RdRp polyprotein has a length greater than 911 amino acids, and the CP protein is some 400 amino acids long.

(ii) Twelve viruses, nine of which are from Teltow Canal and Havel River, are characterized by a long polyprotein (942-1101 amino acids) comprising methyltransferase (pfam01660) and RdRp (pfam00998, cd23179) domains. A second ORF encodes the CP (pfam00729) except for TC-Tombus-LV-28 (Figure S8D). This virus lacks a jellyroll CP but has two overlapping ORFs in different reading frames that encode hypothetical proteins of 336 and 204 amino acids, respectively. Further, TC-Tombus-LV-13—another methyltransferase-expressing virus—exhibits an ORF, which is located between the polyprotein-encoding ORF 1 and the CP-encoding ORF 3. The hypothetical proteins of TC-Tombus-LV-13 and -28 show no similarity to known proteins.

(iii) The RdRp of tombunodavirus UC1, which is assumed to combine features of the tombusviruses and the nodaviruses clusters with several viruses of the Teltow Canal and the Havel River (Figure 1). These viruses exhibit three ORFs encoding a P33-like replicase domain, a catalytic core domain of the RdRp, and a CP. The functional polymerase is expressed by a readthrough mechanism (Figure S8A).

(iv) Four viruses exhibited a CP similar to peptidase A21 (pfam03566). Similar capsid proteins are found in members of *Alphatetraviridae*, *Carmotetraviridae*, *Permutotetraviridae,* and a few unclassified viruses. A phylogenetic analysis of the CP protein (Figure S2) revealed at least four clades with peptidase A21-like CPs. The four Teltow Canal viruses group with two of these clades. The RdRp sequences of TC-Tombus-LV-9 and -88 are available and indicate that these viruses are members of the *Tolivirales* order, whereas TC-Tombus-LV-303 and -335 lack RdRp sequences and hence are untypeable.

(v) The RdRp of TC-Tombus-LV-80 clusters with the RdRp of luteoviruses. Further, its ORF1 exhibits similarity to the luteovirus P1–P2 protein, and a potential frameshift signal (UUUAAAU) overlaps with the UAA stop codon of ORF1 and the start codon of ORF2. However, ORF3 does not encode a CP but a bromo-like movement protein (pfam01573). The available partial sequence lacks a CP gene (Figure S8B).

For further characterization of tombus-like viruses, we aligned some CP sequences of *Tombusviridae* and *Solemoviridae* reference viruses (pfam00894 or pfam00729) and 81 TC-Tombus-LVs/H-Tombus-LVs including the presumed *Tombusviridae* candidates as well as the tombus-like clade A viruses and viruses with similarity to tombunodavirus UC1 (Figure S3).

The CP alignment once more confirmed the two CP variants, one with a P-domain and one that lacks it. Next, we clipped off the P-domain-encoding sequences of the alignment and conducted ML tree inference with IQ-TREE2. The result is presented in Figure S3. Noteworthy, the clustering of CP sequences does not correlate with the RdRp branching, most obvious for luteoviruses, polemoviruses, and sobemoviruses, but also aureusviruses and gammacarmoviruses show inconsistent grouping. The tombus-like clade A viruses were confirmed as a monophyletic cluster within the *Tombusviridae* members. As previously described [39], tombunodavirus UC1 clustered with the Plasmopara halstedii A virus (compare Figures S1 and S3). The respective clade also contains Sclerophthora macrospora A virus and several TC-Tombus-LVs and H-Tombus-LVs.

#### 3.2.2. *Solemoviridae*

DIAMOND assigned 190 scaffolds/175 contigs of the Teltow Canal sample and 58 scaffolds/47 contigs of the Havel River sample to the *Solemoviridae* family. Viruses of this family have an icosahedral T = 3 capsid with a small, non-segmented +ssRNA genome of 4–4.6 kb [40]. The ORFs encode four or five proteins: (i) an RNA silencing suppressor, (ii) a short polyprotein (P2a) which is autocatalytically processed into a membrane anchor, a serine proteinase, a genome-associated VPg, and an uncharacterized C-terminal protein, (iii) a large polyprotein (P2ab) with identical N-terminal domains of membrane anchor, serine proteinase and VPg fused to a C-terminal RdRp catalytic core domain by a -1 ribosomal frameshift, and (iv) a capsid protein; (v) sobemoviruses have an additional ORF x [41]. Whereas the polyproteins and protein x are expressed by a leaky scanning mechanism, the capsid protein is translated from a sgRNA. The family comprises four genera with altogether 60 species. Whereas all solemoviruses share a similar RdRp, two phylogenetically distinct CPs are found: the CP of sobemoviruses and polemoviruses exhibits similarity to the protein family pfam00729. In contrast, the CP of the recently incorporated enamoviruses and poleroviruses have similarities with the protein family pfam00894. Despite this difference, the CPs of all solemoviruses lack a P domain.

**Figure 3:**
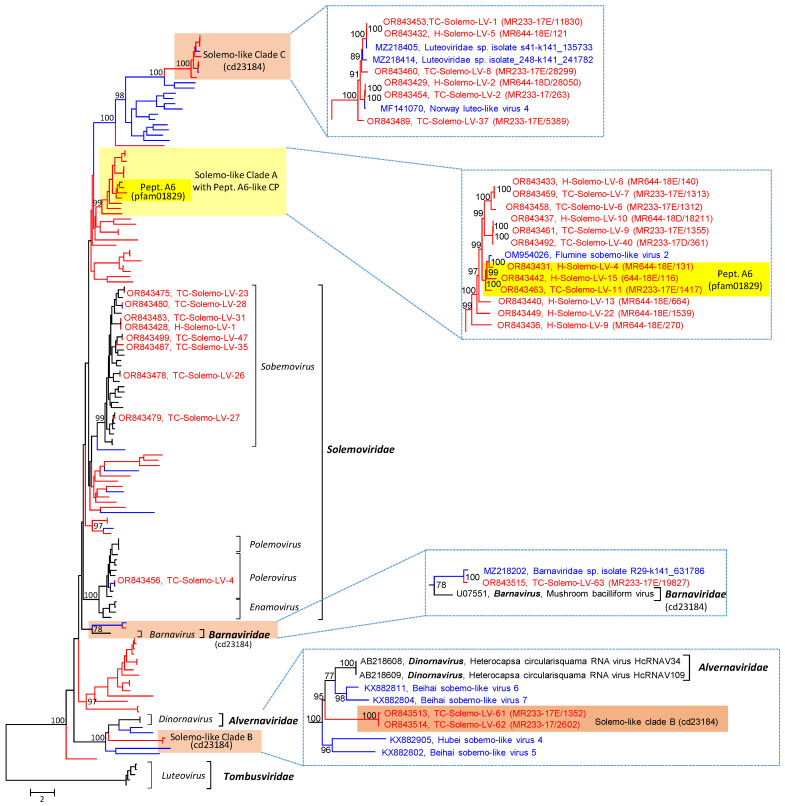
Phylogenetic analysis of solemo-like viruses. The dataset includes 144 RdRp sequences and comprises 72 solemo-like viruses of Teltow Canal and Havel River (red lines), 28 unclassified viruses downloaded from GenBank (blue lines), 43 reference viruses of the *Solemoviridae*, *Tombusviridae*, *Alvernaviridae*, and *Barnaviridae* (black lines). Amino acid sequences were aligned with MEGA and used for tree inference with IQ-TREE 2 (optimal substitution model: VT+F+R9; 10,000 ultrafast replications). The tree was arbitrarily rooted with tombusviruses. The scale bar indicates substitutions per site. Viruses of solemo-like clade A are highlighted, with the dark yellow box marking viruses with presumed peptidase A6 activity of their capsid protein and the light yellow box indicating viruses with similarity to peptidase A6 but lacking proteinase activity. Brown boxes highlight viruses with barna-like RdRp (cd23184), i.e., barnavirus, TC-Solemo-LV-63, and solemo-like clades B and C. Numbers at nodes indicate ultrafast bootstrap support greater than 75%. Details of the tree are presented in Figure S2.

The solemo-like viruses comprise the second largest group in our datasets; 59 scaffolds/68 contigs greater than 1 kb of the Teltow Canal sample and 23 scaffolds/27 contigs of the Havel River sample were further analyzed. The scaffold sizes range from 1.025 to 5.994 kb (mean 3.0436 kb) for the Teltow Canal sample and 1.195 to 6.59 kb (mean 3.6939 kb) for the Havel River sample. The clc contigs of the Teltow Canal sample ranged from 1.011 to 5.547 kb (mean 2.4498 kb), whereas those of the Havel ranged from 1.003 to 6.159 (mean 3.4517 kb). The RdRp tree, which includes reference sequences of the *Solemoviridae*, *Barnaviridae*, and *Alvernaviridae,* reveals eight candidates of the *Solemoviridae* family. The remaining 40 TC-Solemo-LVs and 23 H-Solemo-LVs likely represent novel taxa (Figure 3 and Figure S3). These include viruses of several interesting clades:

(i) Thirteen viruses of solemo-like clade A have larger genomes than the known members of the *Sobelivirales*. The genome layout resembles that of solemoviruses with three ORFs (Figure S8E). ORF 1 encodes a polyprotein of almost 1000 amino acids. This polyprotein has a trypsin-like proteinase domain (pfam13365) with a characteristic Gx**S**Gx_11_GxH motif. ORF2 codes for a solemovirus-like RdRp (cd23180). It has a catalytic domain with DxxxxD—SG—GDD motifs. The RdRp domain is fused to the polyprotein by a -1 frameshift at a UUUAAAC heptanucleotide/stem-loop structure. A third ORF codes for an alphanoda-like CP. The CP of alphanodaviruses is encoded by RNA 2 of the bipartite genome and has a carboxypeptidase activity. It belongs to the peptidase A6 protein family (pfam 01829), which is characterized by a single active site aspartic acid residue (D_86_ of paradigmatic flock house virus CP) [42]. This peptidase activity autocatalytically releases the β- and γ-polypeptides by cleavage of the CP precursor (α) at asparagine 363 (flock house virus numbering), which is necessary for RNA release upon endocytosis. Pairwise nucleotide p-distances between the CPs of flock house virus and the solemo-like clade A viruses range from 56.3 to 71.8%. A total of 3 of 13 solemo-like clade A viruses exhibit peptidase A6 features, i.e., a conserved active site aspartic acid corresponding to flock house virus CP D_86_ and the conserved N_363_ cleavage site. The remaining solemo-like clade A viruses with available CP sequences lack these features but still have significant similarities to the alphanodavirus CP (Figures S2 and S3). Whether solemo-like clade A viruses express an RNA silencing suppressor or a protein x is unclear.

(ii) The majority of the solemo-like viruses identified in this study have an RdRp type 4 of the cd23180 protein family except for solemo-like clades B and C with barnavirus-like RdRp (cd23184). As shown in Figure 3 and Figure S4, viruses of the solemo-like clade B group are close to the alvernaviruses but differ from these viruses by a long polyprotein 1 with a C-terminal RdRp domain and two ORFs encoding unknown proteins. Both proteins are highly divergent compared to the corresponding proteins of the *Sobelivirales*. Solemo-like clade C comprises 11 highly divergent viruses with genome lengths greater than 6 kb. These viruses express three proteins: a polyprotein with trypsin-like proteinase and RdRp domains, a hypothetical protein with weak similarity to the parvovirus CP, and a third protein with strong similarity to viral CPs with S-domain (pfam00729). Solemo-like clades B and C likely represent novel higher-order taxa of the *Sobelivirales* (Figure S8E).

#### 3.2.3. *Virgaviridae*

DIAMOND identified 498 scaffolds/232 contigs of the Teltow Canal sample and 108 scaffolds/78 contigs of the Havel River sample belonging to the *Virgaviridae* of the *Martellivirales* order (Table 1). Less than 10% of these sequences had lengths greater than 1 kb and were further analyzed. Virgaviruses have rod-shaped virions with +ssRNA genomes of 6.3 to 13 kb. The family comprises 7 genera with 60 species [43]. Members of the *Tobamovirus* genus have monopartite genomes, whereas the remaining virgaviruses have two or three genome segments. All virgaviruses infect plants. Their transmission routes include insects, nematodes, pollen/seeds, or mechanical mechanisms without vectors.

**Figure 4:**
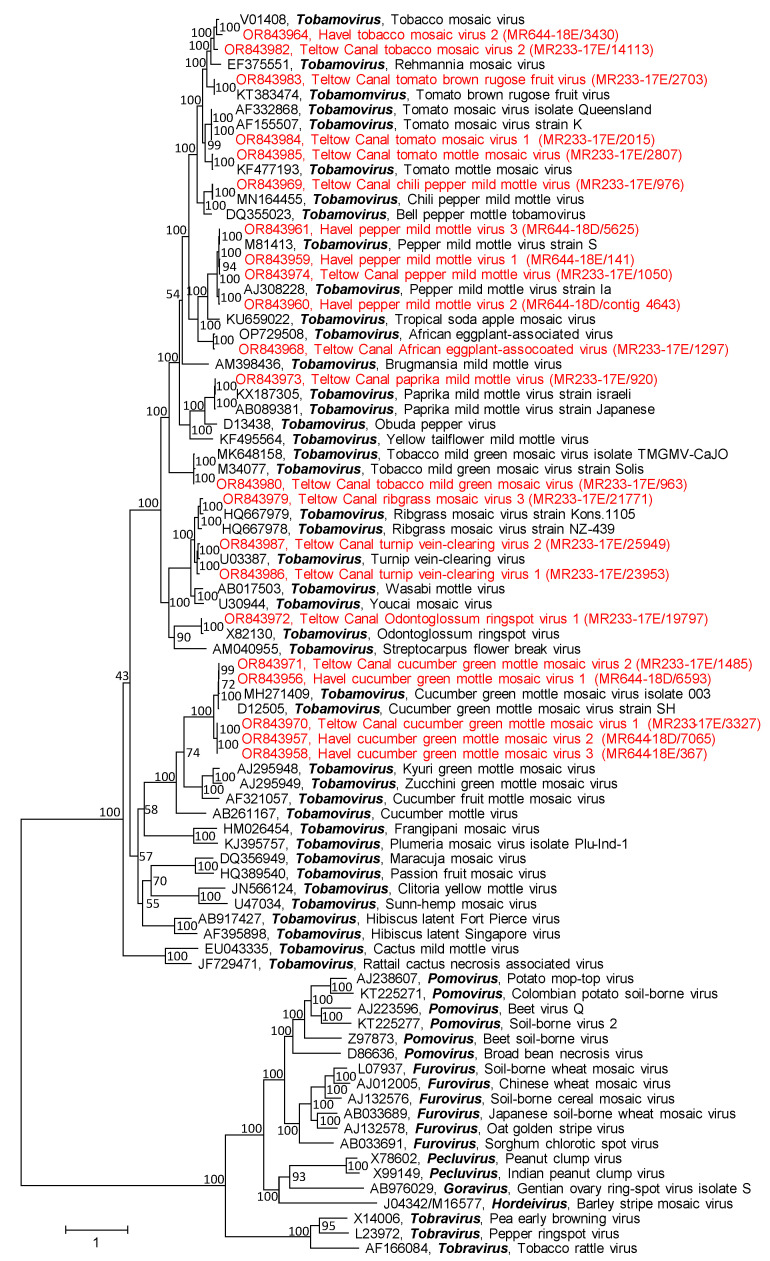
Phylogenetic analysis of virgavirus RdRp. The dataset includes RdRp sequences of 59 reference viruses (printed in black) representing all genera of the *Virgaviridae,* plus 22 sequences from Teltow Canal and Havel River virgaviruses (printed in red). Amino acid sequences were aligned with MEGA and used for tree inference with IQTREE 2 (optimal substitution model: Q.yeast+F+I+G4; 1000 replications). The scale bar indicates substitutions per site. Presented are GenBank accession numbers, virus names, and genus names. Numbers at nodes indicate bootstrap support.

In contrast to the tombus-like viruses and solemo-like viruses, all investigated virgavirus sequences with lengths greater than 1 kb were easily assigned. They belong to the *Tobamovirus* genus (Figure 4).

#### 3.2.4. *Endornaviridae*

**Figure 5:**
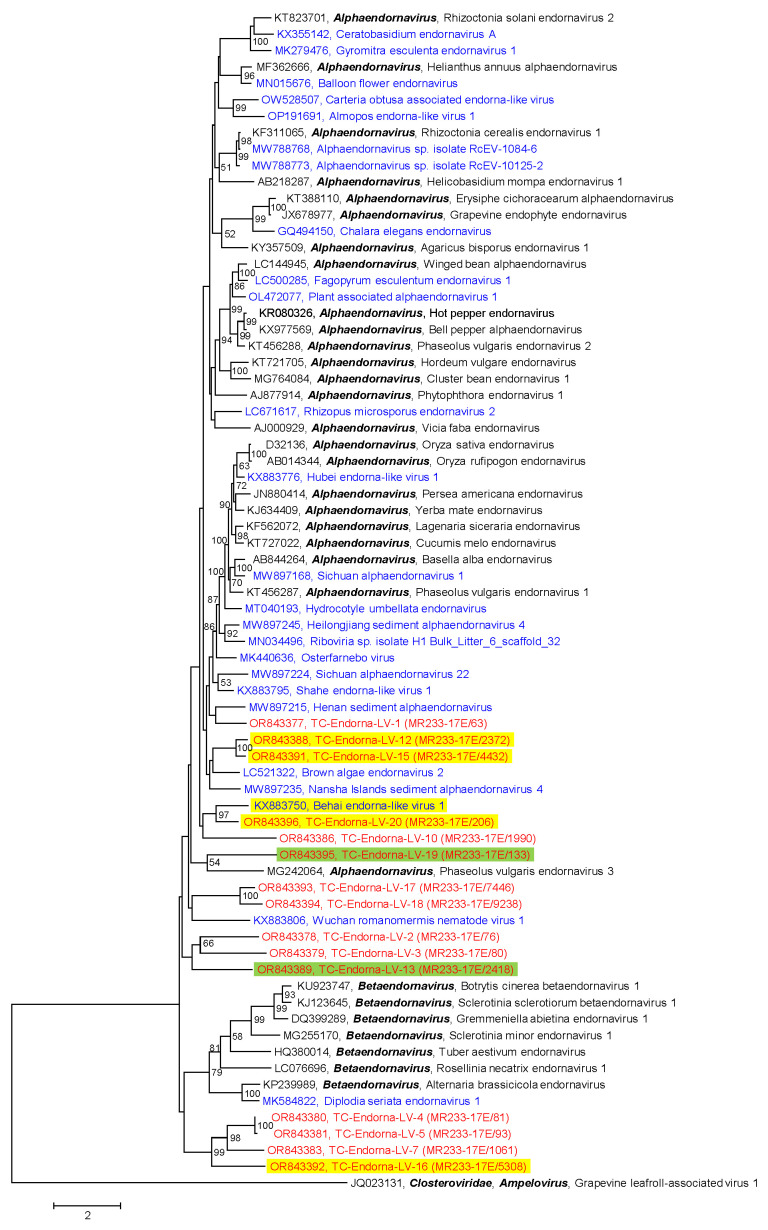
Phylogenetic analysis of endorna-like viruses. The dataset includes 15 RdRp sequences of endorna-like viruses from Teltow Canal (TC-Endorna-LVs) and Havel River (H-Endorna-LVs) (printed in red), 32 sequences of reference viruses (printed in black), and 25 sequences of unclassified endorna-like viruses downloaded from GenBank (printed in blue). Amino acid sequences were aligned with MEGA and used for tree inference with IQTREE 2 (optimal substitution model: Q.pfam+F+R6; 1000 replications). The tree was rooted with grapevine leafroll-associated virus 1 (*Closteroviridae*). Numbers at nodes indicate bootstrap support greater than 50%. The scale bar indicates substitutions per site. Viruses that suppress UAG and UGA termination codons are highlighted in yellow; two viruses that suppress all three termination codons are highlighted in green.

Another family of the *Martellivirales* order that was present in our samples of the Teltow Canal and Havel River is the *Endornaviridae* family. Endornaviruses infect plants, fungi, and oomycetes. These viruses lack capsids but have been associated with cytoplasmic membrane vesicles [44]. The large +ssRNA genome (9.7–17.6 kb) encodes a single polyprotein with helicase, capsular polysaccharide synthase, glycosyltransferase, and RdRp domains. The two genera of this family are distinguished by genome lengths and phylogenetic clustering of the RdRp. We identified 20 endornavirus sequences with lengths from 2030 nt to 15,160 nt in the Teltow Canal sample but no sequence greater than 1 kb in the Havel River sample. Phylogenetic analysis of the RdRp revealed that all Teltow Canal endorna-like viruses (TC-Endorna-LV-1 to -20) are novel and likely belong to the alphaendornaviruses (Figure 5). Nine of these viruses use an alternative genetic code that suppresses UAG and UAA termination codons. Two of the nine viruses with alternative genetic codes, TC-Endorna-LV-13 and -19, additionally suppress the UGA stop codon. The available, partial genome of TC-EndornaV-19 (11,565 nt, 3855 amino acids) exhibits altogether 200 UAA and UAG stop codons (5.8%) plus 48 in-frame UGA stop codons (1.2%), which are presumably suppressed by an alternative genetic code. Likewise, the TC-EndornaV-13 genome (4059 nt, 1353 amino acids) possesses 124 UAA and UAG codons (9.2%) plus 13 UGA codons (1%). In both viruses, termination codons are represented excessively. The use of an alternative translation code suggests unicellular algae or protist hosts of these endorna-like viruses. TC-Endorna-LV-1 exhibits two ORFs. The first ORF encodes a protein of 1919 amino acids with an endorna-like RdRp domain (1.26 × 10^−101^) at its C-terminus. This protein is apparently incomplete and lacks its N-terminus. The second ORF is 3′-truncated and encodes a protein of greater than 3107 amino acids with no similarity to known proteins of the GenBank.

#### 3.2.5. Poty-Like Viruses

DIAMOND assigned 17 scaffolds and 22 contigs greater than 1 kb of the Teltow Canal sample to the *Potyviridae* and none of the Havel River sample (Table 1). The *Potyviridae* family is the single member of the *Patatavirales* order. These viruses have flexuous, rod-like virions and +ssRNAs with lengths of 8–11 kb. Eleven of the 12 genera have monopartite genomes encoding one long polyprotein; only bymoviruses have a bipartite genome. All viruses in this family infect plants. They are transmitted by arthropods except for the bymoviruses, which are passed on by *Polymyxa graminis*, a plasmodiophorid [45]. In addition to the acknowledged members of the family, poty-like viruses have been described on the basis of RdRp sequence similarities but with little information on virion structure, hosts, replication mechanisms, pathogenesis, etc. These viruses have been named plastroviruses, protopotyviruses, flumine astroviruses, and bufivirus (see [25,46,47] and GenBank acc. no. KF510032, Greninger et al. unpublished). Available sequence data suggest that poty-like viruses have smaller genomes (less than 7 kb) than their eponyms with various genome layouts. Their highly divergent sequences span the gap between the *Astroviridae* and *Potyviridae* families.

**Figure 6:**
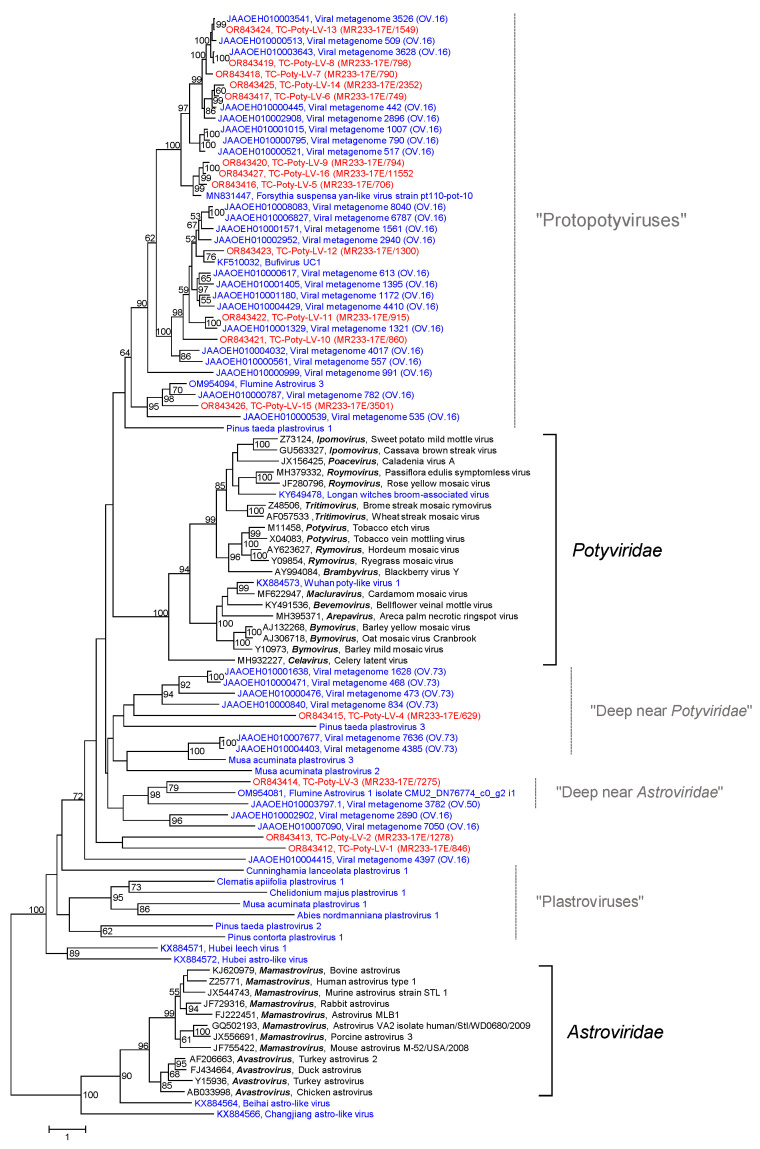
Phylogenetic analysis of poty-like viruses. The dataset includes 100 RdRp sequences comprising 16 poty-like viruses of Teltow Canal and Havel River (printed in red), 19 reference viruses of the *Potyviridae* (printed in black), 12 reference viruses of the *Astroviridae* (printed in black), and 53 sequences of unclassified poty-like viruses downloaded from GenBank or other sources (printed in blue). Amino acid sequences were aligned with MEGA and used for tree inference with IQTREE 2 (optimal substitution model: Q.pfam+F+R7; 1000 replications). The tree was rooted with astroviruses. Numbers at nodes indicate bootstrap support greater than 50%. The scale bar indicates substitutions per site. Square brackets indicate the reference viruses. Thin lines mark protopotyviruses, plastroviruses, and virus groups denoted “deep near *Potyviridae*” and “deep near *Astroviridae*”.

A BLAST search with each of the Teltow Canal potyvirus candidate sequences revealed only weak similarity to the proper potyviruses but stronger similarity to bufivirus UC1 (acc. no. KF510032), some flumine astroviruses and the Forsythia suspensa yan-like virus pt110-pot-10 (acc. no. MN831447). Therefore, we conducted a phylogenetic analysis including reference sequences of the *Potyviridae* (*n* = 21) and *Astroviridae* (*n* = 12) plus a number of unassigned poty-like or astro-like viruses obtained from GenBank (*n* = 40) as well as sequences of 11 plastroviruses (sequence data have not been deposited in public databases but are available from the supplement of [46]). Sixteen sequences of Teltow Canal poty-like viruses (TC-Poty-LVs) completed this dataset. The phylogenetic tree confirmed the BLAST result: all 16 TC-Poty-LVs clustered with poty-like viruses but not with acknowledged members of the *Potyviridae* and *Astroviridae* (Figure 6). The tree topology, however, has to be treated cautiously. Due to the high sequence divergence, some branches have long ML distances, and several nodes lack significant bootstrap support. One major clade comprises 22 protopotyviruses of the OV.16 lineage [25], Forsythia suspensa yan-like virus pt110-pot-10 [48], bufivirus UC1 (acc. no. KF510032) and 12 TC-Poty-LVs. These viruses have two ORFs, each encoding a polyprotein. The C-terminus of polyprotein 1 (>1200 amino acids) shares some similarities with the potyvirus nuclear inclusion protein A, which might indicate a proteinase activity. The significance, however, is unclear as the conserved active site sequence motif CxSG is modified to CxxSG. The second polyprotein (ca. 950–1000 amino acids) has an RdRp (cd23167) and a CP domain with jellyroll fold (pfam00729), suggesting icosahedral rather than rod-like virions. Other viruses of the OV.16, OV.73 (“deep near *Potyviridae*”), and OV.50 lineages (“deep near *Astroviridae*”) [25], the plastroviruses [46] and few viruses of other studies have various genome layouts indicating taxonomic diversity of these viruses.

#### 3.2.6. *Partitiviridae*

DIAMOND assigned 14 scaffolds/9 contigs >1 kb of the Teltow Canal sample and 1 scaffold/1 contig of the Havel River sample to the *Partitiviridae* (Table 1). Our BLAST search revealed 12 RNA 1 sequences and three RNA 2 sequences. Members of the *Partitiviridae* have bisegmented double-stranded RNA (dsRNA) genomes. The genome segments have a length of 1.4 to 3 kb each and are separately packed in virions. RNA 1 encodes the RdRp, whereas RNA 2 codes for a capsid protein. The icosahedral capsids are assembled from 60 CP dimers (T = 1 symmetry). They contain one RNA molecule packed together with one or two RdRp molecules. RdRp molecules are capsid-associated; hence, RNA synthesis occurs inside the particle. Within their persistently infected hosts, partitiviruses are transmitted by cell division. In addition, vertical transmission occurs from the ovule to the seed embryo in plants and during sporogenesis in fungi. There are five genera with 45 species plus 15 species which are unassigned to a genus. The viruses of four of the five genera infect plants and fungi, Cryptosporidium parvum virus 1, and the single member of the fifth genus, *Cryspovirus*, infects *Cryptosporidium* protozoa.

Typing the TC-Partiti-LVs on the basis of the RdRp sequence proved difficult (Figure S6). Two viruses, TC-Partiti-LV-3 and -4, clustered with gammapartitiviruses and TC-Partiti-LV-11 showed some similarity with *Cryptosporidium parvum virus 1* and may be considered a new cryspovirus, but the remaining TC-Partiti-LVs grouped distinctly with unclassified partiti-like viruses. Four clades of unclassified partiti-like viruses were observed, plus a branch with a single virus, TC-Partiti-LV-12 (Figure S6A). All three RNA 2 segments could be typed. One sequence grouped with fungi-infecting betapartitiviruses (Havel partiti-like virus 1, Figure S6B), another one grouped with *Penicillium stoloniferum virus F* and is likely the second segment of TC-Partiti-LV-4 (Figure S6C); the third one showed 97.3% amino acid identity to Wuhan insect virus 23 and hence is presumable RNA 2 of TC-Partiti-LV-6 (Figure S6D).

#### 3.2.7. Albeto-Like Viruses

Three genera of plant-infecting satellites are not assigned to higher-order taxa. In our Teltow Canal and Havel River samples, sequences with similarity to two of these three genera were detected, *Albetovirus* and *Aumaivirus*. The viruses of both genera are satellites with short +ssRNAs encoding only a CP with jellyroll fold. Genomic RNA is replicated by the RdRp of their corresponding helper viruses of the *Tombusviridae* [49].

**Figure 7:**
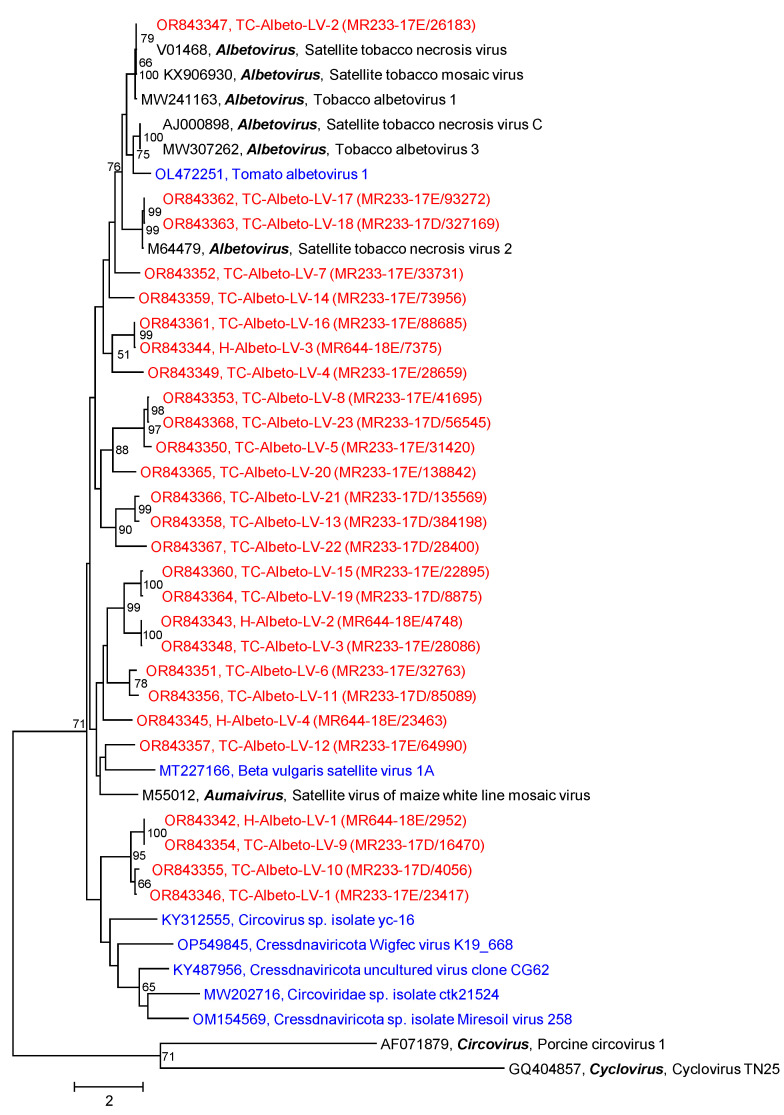
Phylogenetic analysis of albeto-like viruses. The dataset includes 43 sequences of the CP of 27 albeto-like viruses of Teltow Canal and Havel River (printed in red), 9 reference viruses (6 albetoviruses, 1 aumaivirus, 1 circovirus, 1 cyclovirus; printed in black), 2 unclassified albeto-like viruses and 5 CAP protein sequences of unclassified single-stranded DNA viruses (printed in blue). Amino acid sequences were aligned with MEGA and used for tree inference with IQTREE 2 (optimal substitution model: Q.pfam+F+R3; 1000 replications). Numbers at nodes indicate bootstrap support greater than 50%. The scale bar indicates substitutions per site. This is an unrooted tree.

DIAMOND suggested 8 scaffolds/4 contigs greater than 1 kb of the Teltow Canal sample and 3 scaffolds/1 contig of the Havel River sample to *Albetovirus* (Table 1). As the CP of albetoviruses and aumaiviruses has a length of ca. 200–270 amino acids, sequences smaller than 1 kb were also checked for (almost) complete CP genes. Altogether, 23 Teltow Canal albeto-like viruses (TC-Albeto-LVs) and four Havel albeto-like viruses (H-Albeto-LVs) were investigated finally. In addition to albetoviruses and the satellite virus of maize white line mosaic virus (*Aumaivirus*), BLAST also suggested ssDNA viruses (circoviruses and unclassified candidates of the *Cressdnaviricota*) which have similar structural proteins. Therefore, Cap sequences of five ssDNA viruses plus two reference viruses representing the genera *Circovirus* and *Cyclovirus* were included in the CP alignment. The CP phylogenetic tree (Figure 7) revealed TC-Albeto-LV-2 being most closely related to satellite tobacco mosaic virus and satellite tobacco necrosis virus (99.5% amino acid identity). For the remaining TC-Albeto-LVs and H-Albeto-LVs, the CP amino acid identities in comparison to the three acknowledged albetoviruses and the satellite virus of maize white line mosaic virus range from 25–95% which makes an assignment to either genus difficult. The amino acid identities to the unclassified ssDNA viruses are smaller (22–28%) and minimal for circovirus and cyclovirus (10–14%).

#### 3.2.8. *Aspiviridae*

Aspiviruses are segmented, negative-stranded RNA (–ssRNA) viruses with flexuous, filamentous, non-enveloped virions. The helical nucleocapsids consist of a single type of CP and one of the three or four genome segments. As yet, the replication mechanism has not been characterized. The single genus, *Ophiovirus*, comprises seven species. The complete genome sequences of five species are available. Plants serve as hosts, but numerous aspivirus-like sequences have been associated with fungi, oomycetes, and insects [50].

**Figure 8:**
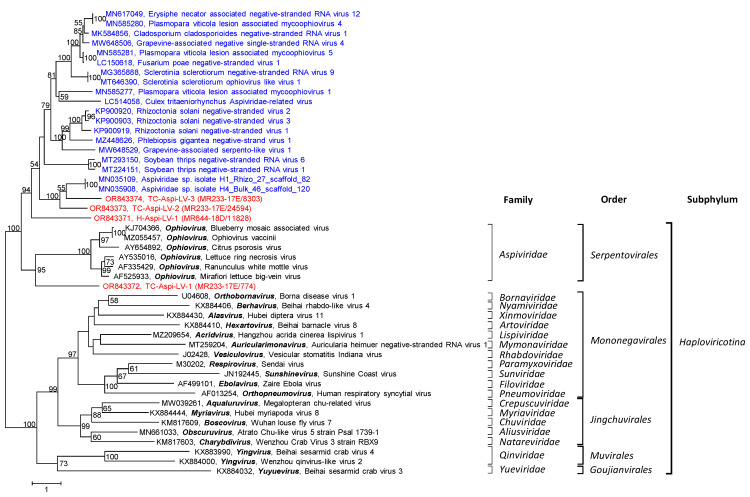
Phylogenetic analysis of aspi-like viruses. RdRp sequences of four aspi-like viruses from Teltow Canal and Havel (printed in red), 25 reference viruses of the orders *Serpentovirales*, *Mononegavirales*, *Jingchuvirales*, *Muvirales* and *Goujianvirales* (printed in black), and 19 unclassified viruses (printed in blue) were aligned with MEGA and used for tree inference with IQTREE2 (optimal substitution model: Q.pfam+F+R6, 1000 replications). The tree was arbitrarily rooted. Presented are GenBank acc. nos., virus names, and genus designations. Square brackets indicate the families, orders, and the subphylum. Numbers at nodes indicate bootstrap support greater than 50%. The scale bar indicates substitutions per site.

Diamond identified 3 scaffolds/2 contigs >1 kb in the Teltow Canal sample and 1 scaffold/1 contig in the Havel River sample (Table 1). All were confirmed by BLAST. Of these sequences, two complete and two partial RdRps were investigated here. These were named Havel aspi-like virus 1 (H-Aspi-LV-1) and Teltow Canal aspi-like virus 1 to 3 (TC-Aspi-LV-1 to -3). For phylogenetic analysis, the conserved RdRp core modules of 5 ophioviruses, 19 unclassified aspi-like viruses, and 19 reference viruses of the *Mononegavirales*, *Jingchuvirales*, *Muvirales,* and *Goujianvirales* were aligned with H-Aspi-LV-1 and three TC-Aspi-LVs. The result indicates highly divergent aspi-like viruses from the Teltow Canal, Havel River, and other sources (Figure 8).

#### 3.2.9. *Alphaflexiviridae*

Viruses of the *Alphaflexiviridae* have a flexuous, filamentous virion with a +ssRNA of 5.5–9 kb. The family comprises 6 genera with 65 species [51]. The host range includes plants and fungi. Two scaffolds greater than 1 kb of the Teltow Canal sample were assigned to *Potexvirus* and confirmed by BLAST (Table 1). One sequence corresponded to the helicase-encoding gene region, and the other included an almost complete genome with only some 65 nucleotides at the 3′-end missing. Both sequences were aligned with the corresponding reference sequences of all *Tymovirales* families. The RdRp and helicase trees confirmed the BLAST results, two strains of the pepino mosaic virus, genus *Potexvirus* (Figure S7A,B). Ten short scaffolds and eight contigs with a total length of 3.3 kb revealed the presence of cactus virus X in the Teltow Canal sample. Another short scaffold was assigned to Schlumbergera virus X. A single, short scaffold (260 nt) of cactus virus X in the Havel River sample was the only proof of an alphaflexivirus in this waterbody.

#### 3.2.10. *Bromoviridae*

The members of the *Bromoviridae* have tripartite +ssRNA genomes with a total length of about 8 kb. RNA segment 1 encodes a polyprotein with methyltransferase and helicase domains, segment 2 codes for the RdRp, and segment 3 for a movement protein (ORF 3a) and the CP (ORF 3b). Some viruses have a segment 2 with a second ORF encoding a protein involved in cell-to-cell movement, suppression of post-transcriptional gene silencing, and symptom induction. RNA segments have a 5′-cap and act as mRNA, though not polyadenylated. They are packed separately in icosahedral, spherical, or bacilliform capsids, sometimes together with sgRNA, defective interfering RNAs, or satellite RNAs [52].

**Figure 9:**
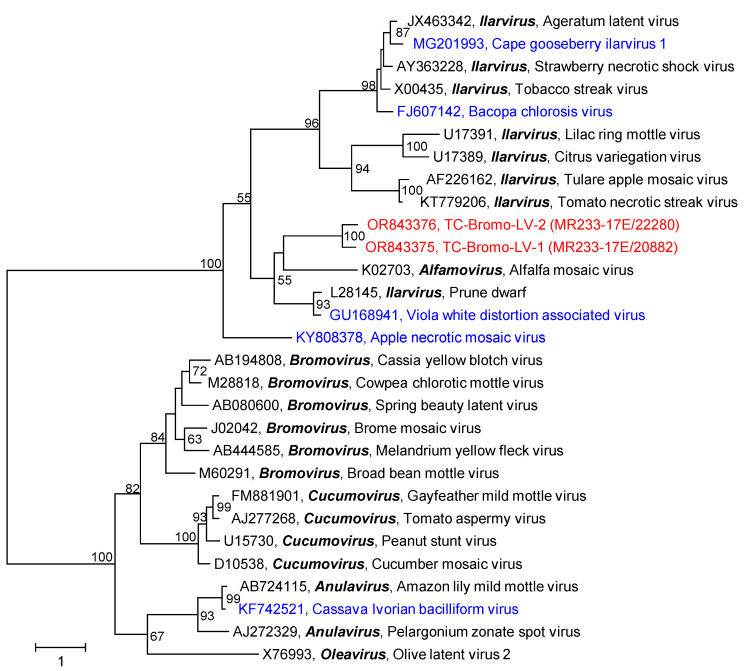
Phylogenetic analysis of bromoviruses. The dataset includes movement protein sequences of two bromoviruses from the Teltow Canal (printed in red), 22 reference viruses of the *Bromoviridae* (printed in black), and 5 unclassified bromoviruses (printed in blue). The sequences were aligned with MEGA and used for tree inference with IQTREE2 (optimal substitution model: Q.pfam+F+R3, 1000 replications). Presented are GenBank acc. nos., virus names, and genus designations. Numbers at nodes indicate bootstrap support greater than 50%. The scale bar indicates substitutions per site. This is an unrooted tree.

Only 3 scaffolds/1 contig greater than 1 kb plus 21 scaffolds/18 contigs smaller than 1 kb were identified in both samples (Table 1). The small sequences represented all three RNA segments of the cucumber mosaic virus (genus *Cucumovirus*), whereas the larger sequences encoded the movement protein (RNA 3) of two novel viruses with similarity to ilarviruses or alfamoviruses (Figure 9). The Havel River sample had no sequences greater than 1 Kb of the *Bromoviridae* family.

## 4. Discussion

In order to investigate the viromes of two freshwater bodies, we collected 50 L samples from the Teltow Canal and the Havel River in the metropolitan area of Berlin, Germany. The Havel River has a near-natural river course with extensive recreational use. By contrast, the Teltow Canal, which connects the rivers Spree and Havel, runs through densely populated districts in the Southwest of Berlin (Figure 1) and receives the discharge of a wastewater treatment plant and drain water after heavy rainfalls. Along both waterbodies, green areas and horticulture may further influence the virome composition of soil and water. Whereas human pathogenic viruses such as enteroviruses, noroviruses, adenoviruses, and hepatitis E viruses are detectable with sensitive PCR protocols only at low levels [53], our previous studies gave no indication of the presence of such viruses in our metagenomes but revealed numerous novel picorna-like viruses and hepeliviruses [27,28]. In the present study, we focused on plant-associated viruses and were able to identify 647 viruses related to 10 of the 30 virus families or floating genera known to comprise plant-infecting viruses. Due to our approach searching the datasets with DIAMOND and BLAST, conserved protein sequences of RdRp, helicase, proteinases, and capsid proteins with jellyroll fold were preferentially detected, whereas highly divergent sequences encoding other proteins may have been missed. In this sense, our set of 647 sequences may be biased, and future re-analyses of our datasets may identify scaffolds/contigs that complete our partial sequences. For the identification of scaffolds/contigs that represent virus sequences, we used DIAMOND in the first step. DIAMOND assigned a plentitude of scaffolds/contigs to viruses (Table 1), but our previous experiences indicated a high proportion of misassignments. Therefore, all sequences greater than 1 kb that had been assigned to plant-infecting viruses were verified by BLAST searches. Again, numerous DIAMOND hits were shown to be wrong at the taxonomic levels of species, genus, and family. We observed yet again the concurrent detection of viruses in both Berlin waterbodies despite the different sampling times (Table 2). Moreover, many of our virus sequences are novel, but some exhibit significant similarity to unclassified viruses from other metagenomic studies—sometimes up to 100%. Examples are Wuhan insect virus 23, Forsythia suspensa yan-like virus, Flumine sobemo-like virus 39, Xufa yellow dwarf virus, Wuhan house centipede virus 6, Poaceae Liege sobemovirus, signal crayfish associated tombus-like virus 2, Changjiang tombus-like virus 2, Sanxia tombus-like virus 2, or Beihai tombus-like virus 13. These unclassified viruses were from environmental samples from coastal water [25], wastewater [39], soil [26], and sediment [47], as well as tissue samples of plants [48,54] and invertebrate animals [55] collected in various parts of the world. Most astonishing is the global occurrence of many (very) similar viruses in quite diverse environments. The ecological function of plant-infecting viruses in the environment is largely obscure, but one may speculate that rivers augment virus distribution and contribute to a wide prevalence. Hence, it will be important progress to identify the hosts and to understand the composition of the various viromes, their common and unique features, and to recognize the factors that govern them. Similar to other previous viromics studies (e.g., [25,55]), the present investigation provides further evidence of an assortment of lineages with gradually similar RdRp sequences. It appears that virus classes and orders are linked by numerous as yet undefined related higher-order taxa. Examples are the albeto-like, poty-like, solemo-like, and tombus-like viruses of our study. Further studies are needed to support this hypothesis and close the knowledge gap with additional sequence data that will enable the development of demarcation criteria for robust virus classification and accommodation of yet unclassified viruses.

Whereas all virgaviruses of both investigated waterbodies belonged to the genus *Tobamovirus* (Figure 4), few of the remaining viruses could be assigned to known genera of the *Alphaflexiviridae*, *Partitiviridae*, *Solemoviridae*, *Tombusviridae* and *Albetovirus*. All other viruses—more than 500—were novel and allowed only a provisional assignment to virus families or orders. Their genetic diversity and their genome layouts indicate that they belong to numerous novel virus taxa. Once more, this prompts the assumption of the existence of a vast dark virus matter and a plethora of dark virus taxa to be investigated in the future.

The coverages of our sequences varied considerably. The highest coverage we observed was that of the Teltow Canal tombus-like virus 172 with 68,815.7 reads/site. Other viruses with coverages greater than 1000 reads/site are compiled in Table 3. The abundance of pepper mild mottle virus (*Virgaviridae*) is not surprising as this virus is a well-known constituent of human feces [56]. Its extremely stable virion retains infectivity for plants after the human gut passage, and a virus is shed by healthy and infected people equally—unlike human pathogens, which are shed only by infected individuals. Hence, this virus has been proposed as an indicator of fecal pollution [57]. The high titer of the two Teltow Canal pepper mild mottle viruses is compatible with the sampling site in an urban area, which is influenced by adjacent horticulture, the discharge of a wastewater plant, and a dense human population in southwest Berlin. It is likely that these viruses withstand wastewater treatment as well as moderate temperatures and the intensity of UV irradiation in the summer months. Likewise, five other virgaviruses had coverages >100 reads/site (compare Table S1). The high prevalence of many other tombus-like, solemo-like, and poty-like sequences is remarkable and may suggest a high abundance of these viruses.

The sequence of TC-Endorna-LV-1 with its two ORFs is remarkable. The significance of the second ORF is unclear as no endorna-like virus with two ORFs has been described as yet, but we have no indication of an assembly artifact. The encoded protein is huge and has no domains with similarity to any known protein.

**Figure 10:**
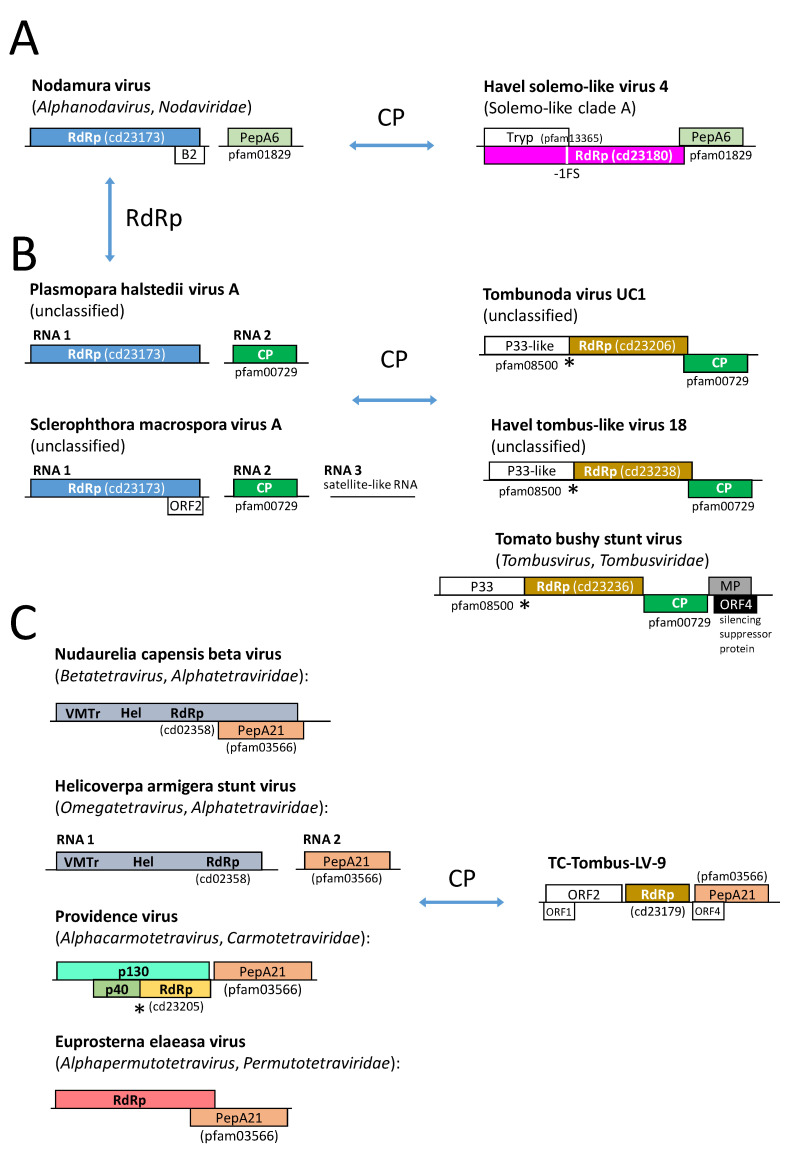
Possible horizontal gene transfer. (**A**) Alphanodavirus is a possible source of peptidase A6-like CP of solemo-like clade A viruses. (**B**) Plasmopara halstedii virus A and Sclerophthora macrospora virus A but not tombunodavirus UC1 exhibit alphanoda-like RdRp and tombus-like CP. (**C**) TC-Tombus-LV-9 with peptidase A21-like CP, which is characteristic of *Alphatetraviridae*, *Carmotetraviridae,* and *Permutotetraviridae*. Boxes represent open reading frames and are denoted with encoded protein names. Colors indicate the similarity of the various ORFs. Arrows indicate possible horizontal gene transfer. The asterisk (*) indicates a translational readthrough mechanism. Conserved protein domain families (cd, pfam) of replicase and structural proteins are presented. Note: ORF4 of tomato bushy stunt virus encodes a silencing suppressor protein; cd23206, cd23236, and cd23238 are subfamilies of cd23205. Abbreviations: RdRp, RNA-dependent RNA polymerase; CP, capsid protein; PepA6, peptidase A6 domain; PepA21, peptidase A21 domain; MP, movement protein, Trp2, trypsin-like peptidase family 2; P33, 32–36 kDa component of functional replicase complex; Hel, helicase domain; VMTr, viral methyltransferase domain.

Tombus-like viruses dominated the investigated scaffolds/contigs of plant-associated viruses in both viromes but only ca. 30 viruses of which may be considered bona fide members of the *Tombusviridae* on the basis of their similarity to the RdRp and their genome layout (see Figure S8A). Several viruses that cluster with luteoviruses display different genome layouts (Figure S8B), but the most tombus-like viruses have highly divergent replicases. Thirty-two sequences cluster more closely with reference sequences of the *Nodamuvirales* than with *Tolivirales* despite the CDD search, which suggested an RdRp of the *Tolivirales* (cd23179) for several of them. These viruses and the many remaining novel tombus-like viruses may be considered to belong to sister clades, which enrich the *Kitrinoviricota* phylum of +ssRNA viruses. Three virus groups deserve closer consideration: viruses using an alternative genetic code, the tombunoda-like viruses, and viruses that encode a capping enzyme:

(i) The use of an alternative genetic code (Figure 2 and Figure S1), presumably translation table 6, which suppresses UAG and UAA termination codons, suggests hosts such as unicellular green algae of the Dasycladaceae family or certain protists such as ciliates and parasitic *Hexamita* diplomonads. Such hosts are compatible with our environmental samples. Several endorna-like viruses also make use of this translation table (compare Figure 5). Furthermore, two endorna-like viruses additionally suppress UGA codons, which is quite peculiar. These viruses exhibit up to 10% in-frame termination codons, which is a remarkable overrepresentation and evidence of both the incorporation of an as-yet unspecified amino acid and the use of an unknown translation termination mechanism. Elucidation of the molecular mechanism of this exceptional process is a worthwhile proposition but requires the isolation of the virus and the identification of its host.

(ii) Tombunodavirus UC1 (see Figure 2 and Figure S1) was described as a virus “that by BLASTx aligned 35% at the amino acid level to the RNA-dependent RNA polymerase of the tombusviruses olive latent virus 1 and tobacco necrosis virus and 57% at the amino acid level to the coat protein of the nodaviruslike Plasmopara halstedii virus A” [39]. This characterization could be a misleading description. As shown in Figures S2 and S4, the RdRp of tombunodavirus UC1 is tombus-like (cd23206), whereas the RdRp of Plasmopara halstedii virus A and Sclerophthora macrospora virus A shares similarities with the nodavirus RdRp (cd23173). In contrast, the CP of tombunodavirus, Plasmopara halstedii virus A, and Sclerophthora macrospora virus A are clearly tombus-like (pfam00729) and clusters in the same clade together with several viruses of Teltow Canal and Havel River. The CP of alphanodaviruses, however, is a member of the peptidase A6 protein family (pfam01829), and that of betanodaviruses belongs to the protein family pfam11729. Hence, both RdRp and CP of tombunodavirus UC1 are, in fact, tombus-like, whereas Plasmopara halstedii virus A and Sclerophthora macrospora virus A combine the features of tombusviruses and nodaviruses (Figure 10B).

A group of tombus-like viruses with a capping enzyme has a monopartite genome and encodes a large polyprotein with a methyltransferase (pfam01660) and a *Tolivirales*-like RdRp (cd23179). However, the viruses of this group are not monophyletic in our RdRp tree—a finding that may not be significant due to low bootstrap support (Figure 2 and Figure S1). Sinhaliviruses also express methyltransferase and RdRp domains, but from two ORFs, and both proteins belong to different protein families (pfam19223 and cd23174).

The second largest group of plant-associated viruses in our freshwater viromes was solemo-like, with eight viruses likely being members of the *Sobemovirus* genus; another one, TC-Solemo-LV-4, is a polerovirus. Further, twelve viruses are candidates of three or four new genera of the *Solemoviridae* (Figure S4). The remaining solemo-like viruses are highly diverse and cannot be assigned to the *Solemoviridae* family. Solemo-like clade A viruses have an alphanoda-like CP (Figure 8A), which is remarkable as the RdRp of the solemoviruses (cd23180, pfam02123, RdRp 4) and alphanodaviruses (cd23173, pfam00680, RdRp 1) belong to different protein families. Moreover, the solemovirus RdRp clusters in branch 2 of the global RdRp phylogenetic tree and the nodavirus RdRp in branch 3 [7]. Horizontal gene transfer [3] may explain the shuffling of protein domains. In this context, it is noteworthy to mention two clades, the solemo-like clades B and C, which have a barna-like RdRp (cd23184), but neither clade clusters with barnaviruses in the RdRp tree (Figure 3 and Figure S4), nor exhibits a barna-like genome layout.

## 5. Conclusions

A remarkable diversity of viruses related to plant-infecting viruses in two environmental water samples was observed. Their sequence data substantially expand the sequence space of known tombus-like, solemo-like, endorna-like, poty-like and albeto-like viruses. A continuous assembly of new lineages in our phylogenetic trees blurs the sometimes arbitrary lines of demarcation between genera and families. Clues to horizontal gene transfer accumulate and may even suggest an evolutionary link between the +ssRNA satellites and circular ssDNA viruses. The suppression of UAG and UAA termination codons may suggest some unspecified non-plant hosts of the respective viruses which are compatible with our environmental samples. The suppression of all three stop codons, however, may indicate an as-yet-undiscovered alternative genetic code. In view of the many novel viruses described here, the viromes of riverine ecosystems are still underexplored—despite so many viromics studies that have been published in the past. An appraisal that values the contribution of virus communities to the stability of fluvial ecosystems is still lacking. Despite all progress in the analysis of novel viruses, unknown hosts are still an unsolved obstacle in our perception of the role viruses play in ecosystems.

## Figures and Tables

**Table 1:** Scaffolds/contigs of plant-associated RNA viruses as detected by DIAMOND in environmental water samples.

Assignment by DIAMOND to		Teltow Canal (Sample MR233-17)				Havel River (Sample MR644-18)			
Order	Family or Genus	No. of Scaffolds		No. of Contigs		No. of Scaffolds		No. of Contigs	
		Total No.	>1 kb	Total No.	>1 kb	Total No.	>1 kb	Total No.	>1 kb
-	*Albetovirus*	30	8	19	4	8	3	8	1
*Tymovirales*	*Alphaflexiviridae*	26	2	15	2	2	-	-	-
*Durnavirales*	*Amalgaviridae*	2	2 *	-	-	-	-	-	-
*Serpentovirales*	*Aspiviridae*	4	3	4	2	2	1	2	1
-	*Aumaivirus*	-	-	-	-	-	-	-	-
*Hepelivirales*	*Benyviridae*	2	-	-	-	-	-	-	-
*Tymovirales*	*Betaflexiviridae*	9	-	-	-	-	-	-	-
*Ourlivirales*	*Botourmiaviridae*	224	142 *	-	-	-	-	-	-
*Martellivirales*	*Bromoviridae*	19	2	16	1	2	1	2	-
*Martellivirales*	*Closteroviridae*	25	-	-	-	-	-	-	-
*Tymovirales*	*Deltaflexiviridae*	-	-	-	-	-	-	-	-
*Martellivirales*	*Endornaviridae*	119	42	106	31	12	-	8	-
*Bunyavirales*	*Fimoviridae*	-	-	-	-	-	-	-	-
*Tymovirales*	*Gammaflexiviridae*	-	-	-	-	-	-	-	-
*Martellivirales*	*Kitaviridae*	1	1 *	-	-	-	-	-	-
*Martellivirales*	*Mayoviridae*	-	-	-	-	-	-	-	-
*Mononegavirales*	*Mymonaviridae*	1	-	-	-	-	-	-	-
-	*Papanivirus*	-	-	-	-	-	-	-	-
*Durnavirales*	*Partitiviridae*	95	14	61	9	23	1	11	1
*Patatavirales*	*Potyviridae*	34	17	29	22	4	-	2	-
*Mononegavirales*	*Rhabdoviridae*	3 *	1 *	-	-	-	-	-	-
*Picornavirales*	*Secoviridae*	25	5 *	-	-	-	-	-	-
*Reovirales*	*Sedoreoviridae*	-	-	-	-	-	-	-	-
*Sobelivirales*	*Solemoviridae*	190	59	175	68	58	23	47	27
*Reovirales*	*Spinareoviridae*	-	-	-	-	-	-	-	-
*Tolivirales*	*Tombusviridae*	1710	356	1174	295	603	98	395	100
*Bunyavirales*	*Tospoviridae*	-	-	-	-	-	-	-	-
*Tymovirales*	*Tymoviridae*	7	-	-	-	-	-	-	-
*Martellivirales*	*Virgaviridae*	498	30	232	23	108	10	78	7
*-*	*Virtovirus*	-	-	-	-	-	-	-	-
		∑ 2725	∑ 533	∑ 1831	∑ 457	∑ 822	∑ 137	∑ 553	∑ 137

* no plant viruses.

**Table 2:** HISAT2 mapping results.

Reference Viruses of this Study	Teltow Canal Samples				Havel River Sample	
	MR137-16	20161295	MR233-17		MR644-18	
	CLC Contigs	CLC Contigs	CLC Contigs	metaSPAdes Scaffolds	CLC Contigs	metaSPAdes Scaffolds
Havel albeto-like viruses (*n* = 4)	-	-	2	6	5	4
Havel aspi-like virus (*n* = 1)	-	-	-	-	1	1
Havel partiti-like virus (*n* = 1)	-	-	-	-	1	2
Havel solemo-like viruses (*n* = 25)	13	14	64	68	52	35
Havel tombus-like viruses (*n* = 106)	37	55	181	324	206	132
Havel virga-like viruses (*n* = 13)	33	36	27	233	30	25
Teltow Canal albeto-like viruses (*n* = 23)	-	1	28	27	6	8
Teltow Canal alphaflexi-like viruses (*n* = 2)	-	1	4	2	-	-
Teltow Canal aspi-like viruses (*n* = 3)	-	-	5	3	-	-
Teltow Canal bromo-like viruses (*n* = 2)	-	1	3	2	-	1
Teltow Canal endorna-like viruses (*n* = 20)	-	-	57	20	19	31
Teltow Canal partiti-like viruses (*n* = 14)	-	-	28	15	4	9
Teltow Canal poty-like viruses (*n* = 16)	1	19	48	31	48	72
Teltow Canal solemo-like viruses (*n* = 63)	24	67	161	78	60	69
Teltow Canal tombus-like viruses (*n* = 335)	117	319	815	847	274	362
Teltow Canal virga-like viruses (*n* = 21)	36	64	78	105	40	70

**Table 3:** Virus sequences with high coverages.

Virus	Coverage *
TC-Tombus-LV-172	68,815.7
TC-Solemo-LV-9	29,928.8
TC-Tombus-LV-57	20,918.6
Teltow Canal cucumber green mottle mosaic virus 1 (*Virgaviridae*)	12,382.4
Teltow Canal cucumber green mottle mosaic virus 2 (*Virgaviridae*)	9145.23
TC-Tombus-LV-114	5535.93
Teltow Canal pepper mild mottle virus (*Virgaviridae*)	5325.65
TC-Tombus-LV-40	5098.56
TC-Tombus-LV-102	4143.25
H-Tombus-LV-2	3620.59

* mean number of mapping reads per nucleotide site of a sequence.

## Data Availability

BioProject ID: PRJNA1031393; Biosamples: SAMN37935307, SAMN37935308; Short Read Archive: SRR26492743, SRR26492744; GenBank accession numbers: OR843342-OR843988.

## Supplementary Materials

The following supporting information can be downloaded at https://www.mdpi.com/article/10.3390/pathogens12121458/s1. Figure S1: Phylogenetic analysis of tombus-like viruses; Figure S2: Phylogenetic analysis of capsid proteins with similarity to peptidase A21; Figure S3: Phylogenetic analysis of 165 capsid protein sequences; Figure S4: Phylogenetic analysis of solemo-like viruses; Figure S5: Phylogenetic analysis of solemo-like clade A capsid proteins; Figure S6: Phylogenetic analysis of partiti-like viruses; Figure S7: Phylogenetic analysis of alphaflexi-like viruses; Figure S8: Genome layouts; Table S1: Sequence information.
