# Supplementary material for: Exploring the Diversity of Plant-Associated Viruses and Related Viruses in Riverine Freshwater Samples Collected in Berlin, Germany
Source: Pathogens. 2023 Dec 15;12(12):1458. doi: 10.3390/pathogens12121458 (PMC10745976; doi:10.3390/pathogens12121458)
Supplement: Supplementary file 1 [file pathogens-12-01458-s001.zip › pathogens-2753601-supplementary.pdf]

Supplementary material

Exploring the diversity of plant-associated viruses and related viruses in riverine freshwater samples collected in Berlin, Germany

Roland Zell[1], Marco Groth[2], Lukas Selinka[1], Hans-Christoph Selinka[3]

Legends to Supplementary Figures:

Supplementary Figure S1: Phylogenetic analysis of tombus-like viruses. The dataset includes 518 RdRp sequences and is comprised of 282 Teltow Canal tombus-like viruses (TC-Tombus-LV, printed in red), 75 Havel River tombus-like viruses (H-Tombus-LV, printed in red), 99 unclassified tombus-like viruses downloaded from GenBank (printed in blue), 57 reference viruses of the *Tolivirales* and 5 reference viruses of the *Nodamuvirales* (printed in black). Amino acid sequences were aligned with MEGA and used for tree inference with IQTREE 2 (optimal substitution model: VT+F+R10; 10,000 ultrafast replications). The tree was arbitrarily rooted with nodaviruses. The scale bar indicates substitutions per site. Presented are GenBank accession numbers, virus names, and – if available – genus names, (sub-)family names, order names. Yellow and ochre boxes identify viruses which use translation table 6 and express a viral methyltransferase, respectively. Numbers at nodes indicate ultrafast bootstrap support greater 75%.

Supplementary Figure S2: Phylogenetic analysis of capsid proteins with similarity to peptidase A 21. CP sequences of 11 reference viruses of the *Alphatetraviridae*, *Carmotetraviridae*, and *Permutotetraviridae* families (printed in black) were aligned with CP sequences of this study (printed in red) and unclassified viruses retrieved from GenBank (printed in blue). Amino acid sequences were aligned with MEGA and used for tree inference with IQTREE 2 (optimal substitution model: Q-pfam+F+G4; 1000 replications). Numbers at nodes indicate bootstrap support. The scale bar indicates substitutions per site. Presented are GenBank accession numbers, family names (in bold letters), genus names, virus names and strain designations if two or three viruses of a species were included in the alignment.

Supplementary Figure S3: Phylogenetic analysis of 165 capsid protein sequences. The alignment included 54 amino acid sequences of Teltow Canal tombus-like viruses (TC-Tombus-LV, printed in red), 27 sequences of Havel tombus-like viruses (H-Tombus-LV, printed in red), 68 reference viruses of the *Tombusviridae* and *Solemoviridae* (printed in black) and 16 sequences of unclassified viruses (printed in blue). IQTREE2 was used for tree inference (optimal substitution model: Q.pfam+F+R5; 1000 bootstrap replications). The tree was arbitrarily rooted with sequences of the pfam00894 cluster. The scale bar indicates substitutions per site. Presented are GenBank accession numbers, virus names, and – if available – genus names and family names. Numbers at nodes indicate bootstrap support greater 50%. Square brackets indicate the protein family (pfam) annotation. Yellow boxes highlight viruses which use translation table 6. Numbers at nodes indicate ultrafast bootstrap support greater 75%.

Supplementary Figure S4: Phylogenetic analysis of solemo-like viruses. The dataset includes 144 RdRp sequences and is comprised of 48 Teltow Canal solemo-like viruses (TC-solemo-LV, printed in red), 24 Havel River solemo-like viruses (H-Solemo-LV, printed in red), 28 unclassified viruses downloaded from GenBank (printed in blue), 35 reference viruses of the *Solemoviridae* (printed in black), 5 reference viruses of the *Tombusviridae* (printed in black), 2 reference viruses of the *Alvernaviridae* (printed in black), and 1 reference virus of the *Barnaviridae* (printed in black). Amino acid sequences were aligned with MEGA and used for tree inference with IQTREE 2 (optimal substitution model: VT+F+R9; 10,000 ultrafast replications). The tree was arbitrarily rooted with tombusviruses. The scale bar indicates substitutions per site. Presented are GenBank accession numbers, virus names, and – if available – genus names, family names, order names. Yellow boxes highlight viruses of solemo-like clade A. The strong yellow box marks viruses with presumed proteinase activity of their capsid protein, the light yellow box distinguishes viruses with similarity to

peptidase A6 but lacking proteinase activity. Brown boxes highlight viruses with barna-like RdRp (cd23184), i.e., barnavirus, Tk-Solemo-LV-63, and solemo-like clades B and C. Numbers at nodes indicate ultrafast bootstrap support greater 75%.

Supplementary Figure S5: Phylogenetic analysis of solemo-like clade A capsid proteins. CP sequences of 10 solemo-like clade A viruses (printed in red) were aligned with CP sequences of five alphanodaviruses (printed in black). The yellow box indicates three viruses with presumed peptidase A6 activity as judged from the presence of the active site aspartic acid (D_{86} according to flock house virus numbering) and the cleavage site at asparagine 363 (according to flock house virus numbering). Tree inference was conducted with IQTREE2 (substitution model: Q.pfam+F+I+G4, 1000 replications). The tree was arbitrarily rooted with nodaviruses. Numbers at nodes indicate bootstrap support greater 50%. Abbreviations: H-Solemo-LV, Havel River solemo-like virus; TC-Solemo-LV, Teltow Canal solemo-like virus.

Supplementary Figure S6: Phylogenetic analysis of partiti-like viruses. (A) RdRp analysis. The dataset includes 90 RdRp sequences comprised of 12 partiti-like viruses of Teltow Canal, 38 reference viruses of the *Partitiviridae*, and 40 sequences of unclassified partiti-like virused downloaded from GenBank. Amino acid sequences were aligned with MEGA and used for tree inference with IQTREE 2 (optimal substitution model: VT+F+R6; 1000 replications). The tree was arbitrarily rooted with betapartitiviruses. Sqare brackets indicate the members of the five partitivirus genera. **(B) - (D) Capsid protein analysis.** CP sequences of 18 betapartitivirus reference strains, 4 unclassified viruses and Havel partiti-like virus 1 **(B)**, 9 gammapartitiviruses, 4 unclassified viruses and Teltow Canal partiti-like virus 4 **(C)**, and 5 deltapartitiviruses, 5 unclassified viruses and Teltow Canal partiti-like virus 6 **(D)** were aligned with MEGA and used for tree inference with IQTREE2 (optimal substitution models: Q.pfam+F+R4 in **(B)**, LG+F+G4 **(C)**, Q.pfam+F+G4 **(D)**; 1000 replications). Presented are unrooted trees.
Numbers at nodes indicate bootstrap support greater 50%. The scale bar indicates substitutions per site. Color code: red, partiti-like viruses from Teltow Canal and Havel River; black, reference viruses; blue, unclassified viruses from GenBank.

Supplementary Figure S7: Phylogenetic analysis of alphaflexi-like viruses. RdRp **(A)** and helicase sequences **(B)** of Teltow Canal pepino mosaic viruses (printed in red) and 34 reference viruses (*Alphaflexiviridae*, *Betaflexiviridae*, *Deltaflexiviridae*, *Gammaflexiviridae*, and *Tymoviridae*; printed in black) were aligned with MEGA and used for tree inference with IQTREE2 (optimal substitution model: Q.pfam+F+I+G4 **(A)**, Q.pfam+I+G4 **(B)**; 1000 replications). The tree was arbitrarily rooted with the deltaflexiviruses. Presented are GenBank acc. nos., virus names and genus designations. Square brackets indicate families. Numbers at nodes indicate bootstrap support greater 50%. The scale bar indicates substitutions per site.

Supplementary Figure S8: Genome layouts. The genomes of *Procedovirinae*/*Tombusviridae* **(A)**, *Regressovirinae*/*Tombusviridae* **(B)**, and viruses of tombus-like clade A **(C)**, tombus-like viruses with methyltransferase **(D)**, and solemo-like clades A, B and C **(E)** of Teltow Canal and Havel River are presented with the aid of selected sequences. In (**A**), **(B)** and **(E)**, the genome organization of reference viruses is included. Thick lines represent the genomes with boxes indicating open reading frames. The lengths of genomes is given. Short arrows and vertical lines indicate the position of readthrough termination codons of *Procedovirinae* members. -1FS and vertical lines indicate the position of -1-frameshift signals of *Regressovirinae* members. Abbreviations: CP, capsid protein; MP, movement protein; ORF, open reading frame; pept, peptidase; PLA2, phospholipase A2; RdRp, RNA-dependent RNA polymerase; Trp2, trypsin-like peptidase family 2; VMeTr, viral methyltransferase.

Figure S1

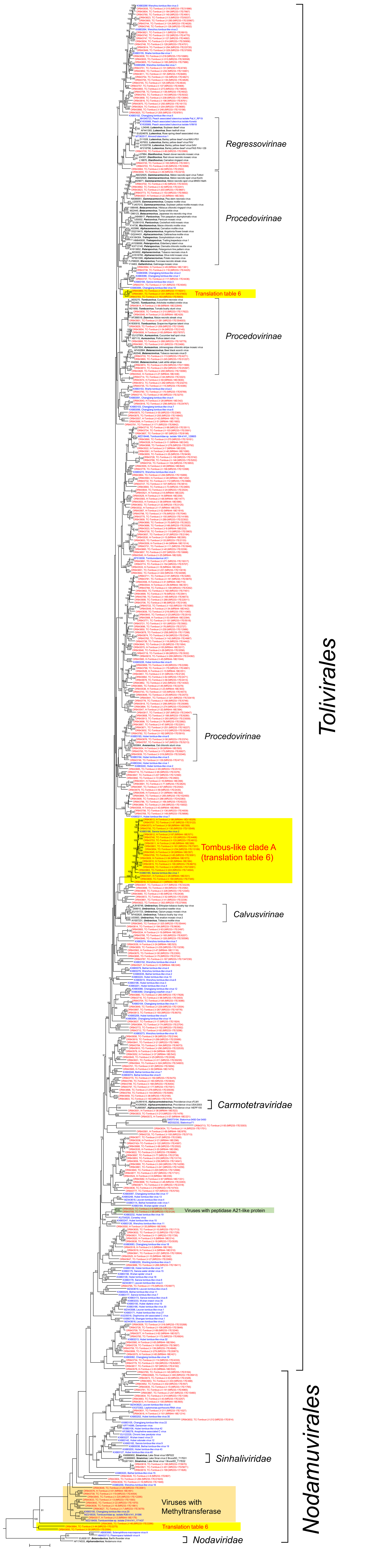

Regressovirinae
Procedovirinae
Translation table 6
Procedovirinae
Tolivirales
Procedovirinae
Tombus-like clade A
(translation table 6)
Calvusvirinae
Carmotetraviridae
Viruses with peptidase A21-like protein
Nodamuvirales
Sinhaliviridae
Viruses with
Methyltransferase
Translation table 6
Nodaviridae

Figure S2

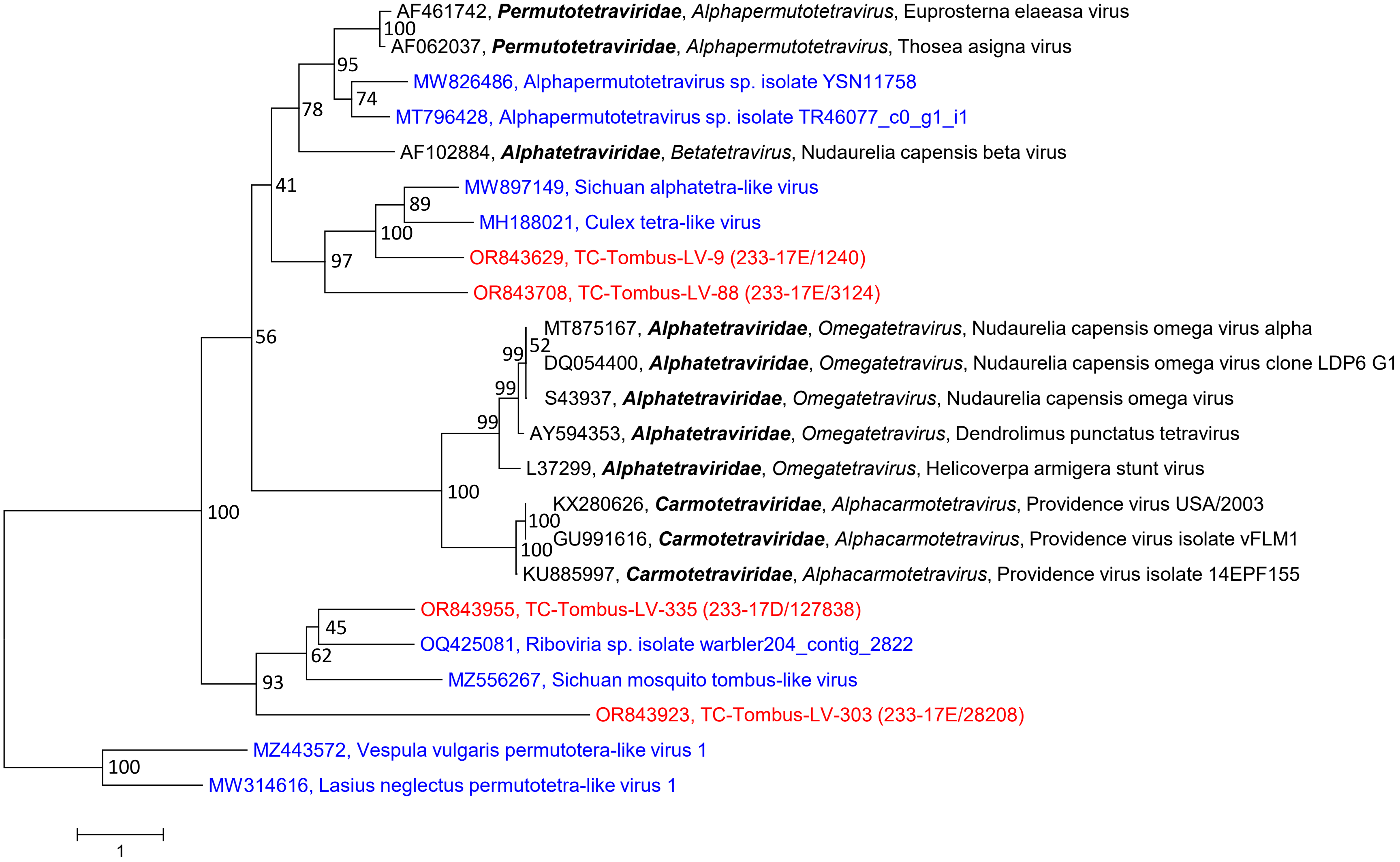

AF461742, **Permutotetraviridae**, *Alphapermutotetravirus*, Euprosterna elaeasa virus
100
AF062037, **Permutotetraviridae**, *Alphapermutotetravirus*, Thosea asigna virus
95
MW826486, Alphapermutotetravirus sp. isolate YSN11758
74
78
MT796428, Alphapermutotetravirus sp. isolate TR46077_c0_g1_i1
AF102884, **Alphatetraviridae**, *Betatetravirus*, Nudaurelia capensis beta virus
41
MW897149, Sichuan alphatetra-like virus
89
MH188021, Culex tetra-like virus
100
OR843629, TC-Tombus-LV-9 (233-17E/1240)
97
OR843708, TC-Tombus-LV-88 (233-17E/3124)
MT875167, **Alphatetraviridae**, *Omegatetravirus*, Nudaurelia capensis omega virus alpha
56
52
99
DQ054400, **Alphatetraviridae**, *Omegatetravirus*, Nudaurelia capensis omega virus clone LDP6 G1
99
S43937, **Alphatetraviridae**, *Omegatetravirus*, Nudaurelia capensis omega virus
99
AY594353, **Alphatetraviridae**, *Omegatetravirus*, Dendrolimus punctatus tetravirus
L37299, **Alphatetraviridae**, *Omegatetravirus*, Helicoverpa armigera stunt virus
100
KX280626, **Carmotetraviridae**, *Alphacarmotetravirus*, Providence virus USA/2003
100
100
100
GU991616, **Carmotetraviridae**, *Alphacarmotetravirus*, Providence virus isolate vFLM1
KU885997, **Carmotetraviridae**, *Alphacarmotetravirus*, Providence virus isolate 14EPF155
OR843955, TC-Tombus-LV-335 (233-17D/127838)
45
OQ425081, Riboviria sp. isolate warbler204_contig_2822
62
MZ556267, Sichuan mosquito tombus-like virus
93
OR843923, TC-Tombus-LV-303 (233-17E/28208)
MZ443572, Vespula vulgaris permutotera-like virus 1
100
MW314616, Lasius neglectus permutotetra-like virus 1
1

Figure S3

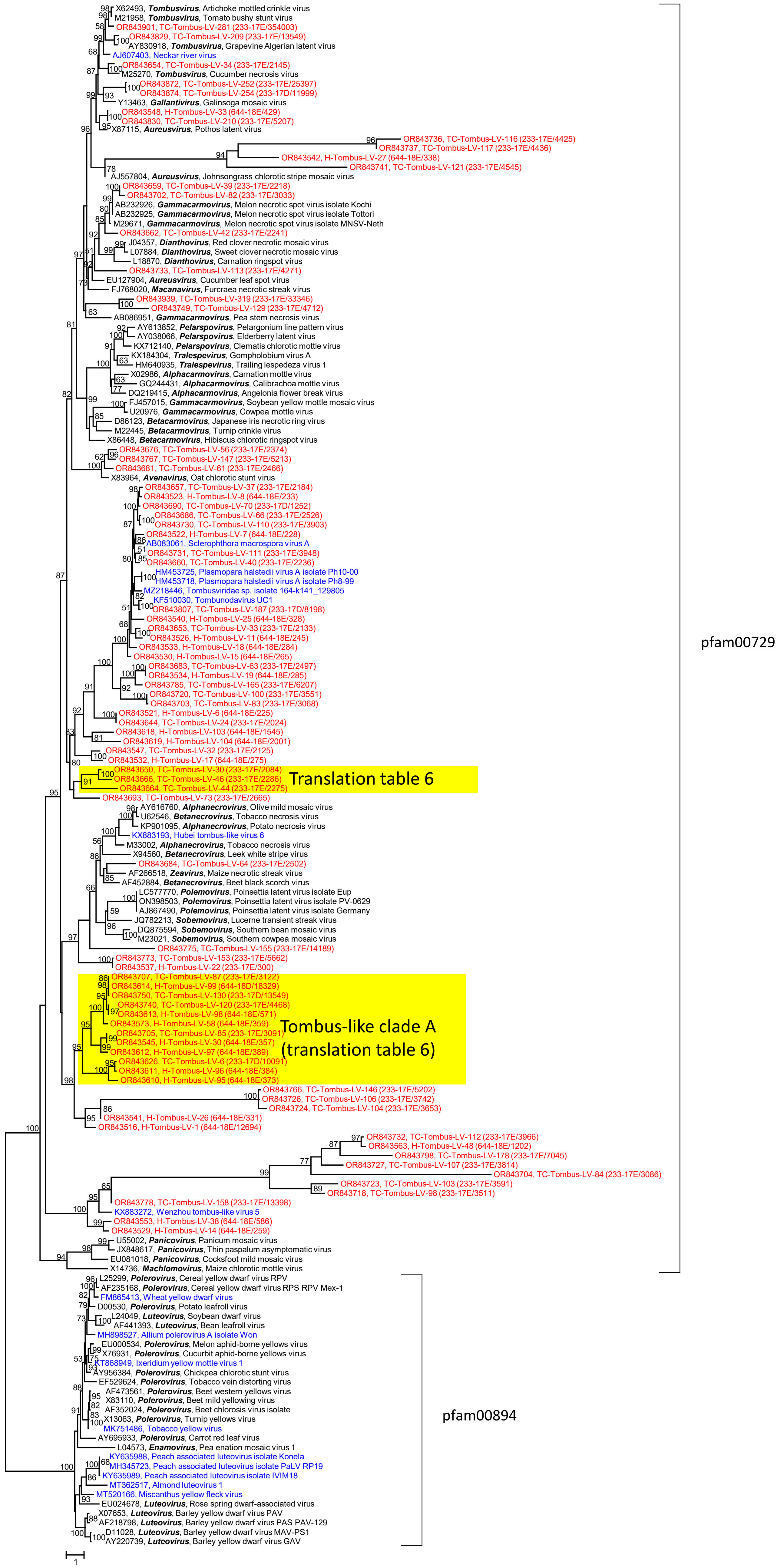

X62493, Tombusvirus, Artichoke mottled crinkle virus
M21958, Tombusvirus, Tomato bushy stunt virus
OR843901, TC-Tombus-LV-281 (233-17E/354003)
OR843829, TC-Tombus-LV-209 (233-17E/13549)
AY830918, Tombusvirus, Grapevine Algerian latent virus
AJ607403, Neckar river virus
OR843654, TC-Tombus-LV-34 (233-17E/2145)
M25270, Tombusvirus, Cucumber necrosis virus
OR843872, TC-Tombus-LV-252 (233-17E/25397)
OR843874, TC-Tombus-LV-254 (233-17D/11999)
Y13463, Gallantivirus, Galinsoga mosaic virus
OR843548, H-Tombus-LV-33 (644-18E/429)
OR843830, TC-Tombus-LV-210 (233-17E/5207)
X87115, Aureusvirus, Pothos latent virus
OR843736, TC-Tombus-LV-116 (233-17E/4425)
OR843737, TC-Tombus-LV-117 (233-17E/4436)
OR843542, H-Tombus-LV-27 (644-18E/338)
OR843741, TC-Tombus-LV-121 (233-17E/4545)
AJ557804, Aureusvirus, Johnsongrass chlorotic stripe mosaic virus
OR843659, TC-Tombus-LV-39 (233-17E/2218)
OR843702, TC-Tombus-LV-82 (233-17E/3033)
AB232926, Gammacarmovirus, Melon necrotic spot virus isolate Kochi
AB232925, Gammacarmovirus, Melon necrotic spot virus isolate Tottori
M29671, Gammacarmovirus, Melon necrotic spot virus isolate MNSV-Neth
OR843662, TC-Tombus-LV-42 (233-17E/2241)
J04357, Dianthovirus, Red clover necrotic mosaic virus
L07884, Dianthovirus, Sweet clover necrotic mosaic virus
L18870, Dianthovirus, Carnation ringspot virus
OR843733, TC-Tombus-LV-113 (233-17E/4271)
EU127904, Aureusvirus, Cucumber leaf spot virus
FJ768020, Macanavirus, Furcraea necrotic streak virus
OR843939, TC-Tombus-LV-319 (233-17E/33346)
OR843749, TC-Tombus-LV-129 (233-17E/4712)
AB086951, Gammacarmovirus, Pea stem necrosis virus
AY613852, Pelarspovirus, Pelargonium line pattern virus
AY038066, Pelarspovirus, Elderberry latent virus
KX712140, Pelarspovirus, Clematis chlorotic mottle virus
KX184304, Tralespevirus, Gompholobium virus A
HM640935, Tralespevirus, Trailing lespedeza virus 1
X02986, Alphacarmovirus, Carnation mottle virus
GQ244431, Alphacarmovirus, Calibrachoa mottle virus
DQ219415, Alphacarmovirus, Angelonia flower break virus
FJ457015, Gammacarmovirus, Soybean yellow mottle mosaic virus
U20976, Gammacarmovirus, Cowpea mottle virus
D86123, Betacarmovirus, Japanese iris necrotic ring virus
M22445, Betacarmovirus, Turnip crinkle virus
X86448, Betacarmovirus, Hibiscus chlorotic ringspot virus
OR843676, TC-Tombus-LV-56 (233-17E/2374)
OR843767, TC-Tombus-LV-147 (233-17E/5213)
OR843681, TC-Tombus-LV-61 (233-17E/2466)
X83964, Avenavirus, Oat chlorotic stunt virus
OR843657, TC-Tombus-LV-37 (233-17E/2184)
OR843523, H-Tombus-LV-8 (644-18E/233)
OR843690, TC-Tombus-LV-70 (233-17D/1252)
OR843686, TC-Tombus-LV-66 (233-17E/2526)
OR843730, TC-Tombus-LV-110 (233-17E/3903)
OR843522, H-Tombus-LV-7 (644-18E/228)
AB083061, Sclerophthora macrospora virus A
OR843731, TC-Tombus-LV-111 (233-17E/3948)
OR843660, TC-Tombus-LV-40 (233-17E/2236)
HM453725, Plasmopara halstedii virus A isolate Ph10-00
HM453718, Plasmopara halstedii virus A isolate Ph8-99
MZ218446, Tombusviridae sp. isolate 164-k141_129805
KF510030, Tombunodavirus UC1
OR843807, TC-Tombus-LV-187 (233-17D/8198)
OR843540, H-Tombus-LV-25 (644-18E/328)
OR843653, TC-Tombus-LV-33 (233-17E/2133)
OR843526, H-Tombus-LV-11 (644-18E/245)
OR843533, H-Tombus-LV-18 (644-18E/284)
OR843530, H-Tombus-LV-15 (644-18E/265)
OR843683, TC-Tombus-LV-63 (233-17E/2497)
OR843534, H-Tombus-LV-19 (644-18E/285)
OR843785, TC-Tombus-LV-165 (233-17E/6207)
OR843720, TC-Tombus-LV-100 (233-17E/3551)
OR843703, TC-Tombus-LV-83 (233-17E/3068)
OR843521, H-Tombus-LV-6 (644-18E/225)
OR843644, TC-Tombus-LV-24 (233-17E/2024)
OR843618, H-Tombus-LV-103 (644-18E/1545)
OR843619, H-Tombus-LV-104 (644-18E/2001)
OR843547, TC-Tombus-LV-32 (233-17E/2125)
OR843532, H-Tombus-LV-17 (644-18E/275)
OR843650, TC-Tombus-LV-30 (233-17E/2084)
OR843666, TC-Tombus-LV-46 (233-17E/2286)
OR843664, TC-Tombus-LV-44 (233-17E/2275)
Translation table 6
OR843693, TC-Tombus-LV-73 (233-17E/2665)
AY616760, Alphanecrovirus, Olive mild mosaic virus
U62546, Betanecrovirus, Tobacco necrosis virus
KP901095, Alphanecrovirus, Potato necrosis virus
KX883193, Hubei tombus-like virus 6
M33002, Alphanecrovirus, Tobacco necrosis virus
X94560, Betanecrovirus, Leek white stripe virus
OR843684, TC-Tombus-LV-64 (233-17E/2502)
AF266518, Zeavirus, Maize necrotic streak virus
AF452884, Betanecrovirus, Beet black scorch virus
LC577770, Polemovirus, Poinsettia latent virus isolate Eup
ON398503, Polemovirus, Poinsettia latent virus isolate PV-0629
AJ867490, Polemovirus, Poinsettia latent virus isolate Germany
JQ782213, Sobemovirus, Lucerne transient streak virus
DQ875594, Sobemovirus, Southern bean mosaic virus
M23021, Sobemovirus, Southern cowpea mosaic virus
OR843775, TC-Tombus-LV-155 (233-17E/14189)
OR843773, TC-Tombus-LV-153 (233-17E/5662)
OR843537, H-Tombus-LV-22 (233-17E/300)
OR843707, TC-Tombus-LV-87 (233-17E/3122)
OR843614, H-Tombus-LV-99 (644-18D/18329)
OR843750, TC-Tombus-LV-130 (233-17D/13549)
OR843740, TC-Tombus-LV-120 (233-17E/4468)
OR843613, H-Tombus-LV-98 (644-18E/571)
OR843573, H-Tombus-LV-58 (644-18E/359)
OR843705, TC-Tombus-LV-85 (233-17E/3091)
OR843545, H-Tombus-LV-30 (644-18E/357)
OR843612, H-Tombus-LV-97 (644-18E/389)
OR843626, TC-Tombus-LV-6 (233-17D/10091)
OR843611, H-Tombus-LV-96 (644-18E/384)
OR843610, H-Tombus-LV-95 (644-18E/373)
Tombus-like clade A
(translation table 6)
OR843766, TC-Tombus-LV-146 (233-17E/5202)
OR843726, TC-Tombus-LV-106 (233-17E/3742)
OR843724, TC-Tombus-LV-104 (233-17E/3653)
OR843541, H-Tombus-LV-26 (644-18E/331)
OR843516, H-Tombus-LV-1 (644-18E/12694)
OR843732, TC-Tombus-LV-112 (233-17E/3966)
OR843563, H-Tombus-LV-48 (644-18E/1202)
OR843798, TC-Tombus-LV-178 (233-17E/7045)
OR843727, TC-Tombus-LV-107 (233-17E/3814)
OR843704, TC-Tombus-LV-84 (233-17E/3086)
OR843723, TC-Tombus-LV-103 (233-17E/3591)
OR843718, TC-Tombus-LV-98 (233-17E/3511)
OR843778, TC-Tombus-LV-158 (233-17E/13398)
KX883272, Wenzhou tombus-like virus 5
OR843553, H-Tombus-LV-38 (644-18E/586)
OR843529, H-Tombus-LV-14 (644-18E/259)
U55002, Panicovirus, Panicum mosaic virus
JX848617, Panicovirus, Thin paspalum asymptomatic virus
EU081018, Panicovirus, Cocksfoot mild mosaic virus
X14736, Machlomovirus, Maize chlorotic mottle virus
pfam00729
L25299, Polerovirus, Cereal yellow dwarf virus RPV
AF235168, Polerovirus, Cereal yellow dwarf virus RPS RPV Mex-1
FM865413, Wheat yellow dwarf virus
D00530, Polerovirus, Potato leafroll virus
L24049, Luteovirus, Soybean dwarf virus
AF441393, Luteovirus, Bean leafroll virus
MH898527, Allium polerovirus A isolate Won
EU000534, Polerovirus, Melon aphid-borne yellows virus
X76931, Polerovirus, Cucurbit aphid-borne yellows virus
KT868949, Ixeridium yellow mottle virus 1
AY956384, Polerovirus, Chickpea chlorotic stunt virus
EF529624, Polerovirus, Tobacco vein distorting virus
AF473561, Polerovirus, Beet western yellows virus
X83110, Polerovirus, Beet mild yellowing virus
AF352024, Polerovirus, Beet chlorosis virus isolate
X13063, Polerovirus, Turnip yellows virus
MK751486, Tobacco yellow virus
AY695933, Polerovirus, Carrot red leaf virus
L04573, Enamovirus, Pea enation mosaic virus 1
KY635988, Peach associated luteovirus isolate Konela
MH345723, Peach associated luteovirus isolate PaLV RP19
KY635989, Peach associated luteovirus isolate IVIM18
MT362517, Almond luteovirus 1
MT520166, Miscanthus yellow fleck virus
EU024678, Luteovirus, Rose spring dwarf-associated virus
X07653, Luteovirus, Barley yellow dwarf virus PAV
AF218798, Luteovirus, Barley yellow dwarf virus PAS PAV-129
D11028, Luteovirus, Barley yellow dwarf virus MAV-PS1
AY220739, Luteovirus, Barley yellow dwarf virus GAV
pfam00894
1

Figure S4

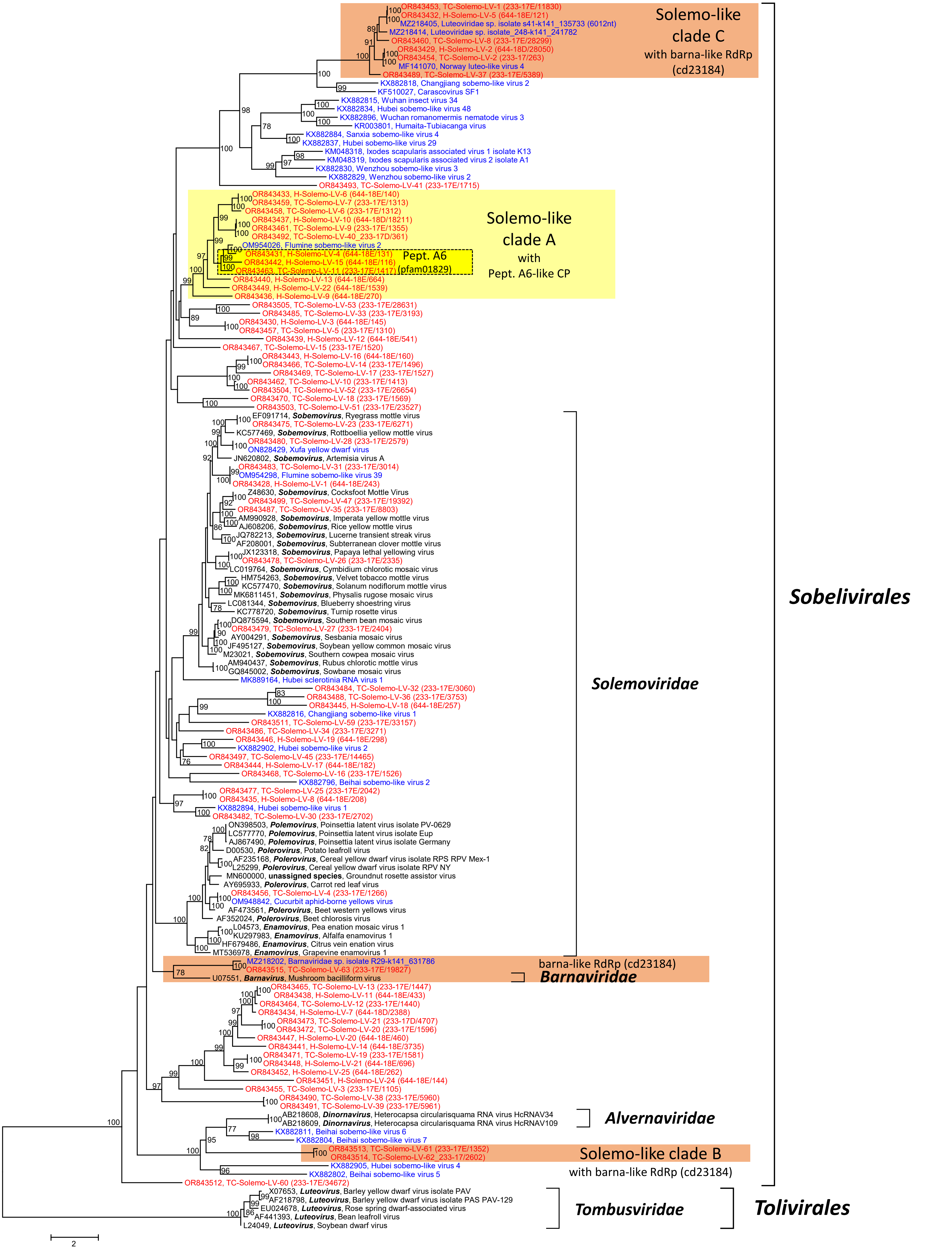
OR843453, TC-Solemo-LV-1 (233-17E/11830)
OR843432, H-Solemo-LV-5 (644-18E/121)
MZ218405, Luteoviridae sp. isolate s41-k141_135733 (6012nt)
MZ218414, Luteoviridae sp. isolate_248-k141_241782
OR843460, TC-Solemo-LV-8 (233-17E/28299)
OR843429, H-Solemo-LV-2 (644-18D/28050)
OR843454, TC-Solemo-LV-2 (233-17/263)
MF141070, Norway luteo-like virus 4
OR843489, TC-Solemo-LV-37 (233-17E/5389)
Solemo-like clade C
with barna-like RdRp (cd23184)
KX882818, Changjiang sobemo-like virus 2
KF510027, Carascovirus SF1
KX882815, Wuhan insect virus 34
KX882834, Hubei sobemo-like virus 48
KX882896, Wuchan romanomermis nematode virus 3
KR003801, Humaita-Tubiacanga virus
KX882884, Sanxia sobemo-like virus 4
KX882837, Hubei sobemo-like virus 29
KM048318, Ixodes scapularis associated virus 1 isolate K13
KM048319, Ixodes scapularis associated virus 2 isolate A1
KX882830, Wenzhou sobemo-like virus 3
KX882829, Wenzhou sobemo-like virus 2
OR843493, TC-Solemo-LV-41 (233-17E/1715)
OR843433, H-Solemo-LV-6 (644-18E/140)
OR843459, TC-Solemo-LV-7 (233-17E/1313)
OR843458, TC-Solemo-LV-6 (233-17E/1312)
OR843437, H-Solemo-LV-10 (644-18D/18211)
OR843461, TC-Solemo-LV-9 (233-17E/1355)
OR843492, TC-Solemo-LV-40_233-17D/361)
OM954026, Flumine sobemo-like virus 2
OR843431, H-Solemo-LV-4 (644-18E/131)
OR843442, H-Solemo-LV-15 (644-18E/116)
OR843463, TC-Solemo-LV-11 (233-17E/1417)
Pept. A6 (pfam01829)
Solemo-like clade A
with Pept. A6-like CP
OR843440, H-Solemo-LV-13 (644-18E/664)
OR843449, H-Solemo-LV-22 (644-18E/1539)
OR843436, H-Solemo-LV-9 (644-18E/270)
OR843505, TC-Solemo-LV-53 (233-17E/28631)
OR843485, TC-Solemo-LV-33 (233-17E/3193)
OR843430, H-Solemo-LV-3 (644-18E/145)
OR843457, TC-Solemo-LV-5 (233-17E/1310)
OR843439, H-Solemo-LV-12 (644-18E/541)
OR843467, TC-Solemo-LV-15 (233-17E/1520)
OR843443, H-Solemo-LV-16 (644-18E/160)
OR843466, TC-Solemo-LV-14 (233-17E/1496)
OR843469, TC-Solemo-LV-17 (233-17E/1527)
OR843462, TC-Solemo-LV-10 (233-17E/1413)
OR843504, TC-Solemo-LV-52 (233-17E/26654)
OR843470, TC-Solemo-LV-18 (233-17E/1569)
OR843503, TC-Solemo-LV-51 (233-17E/23527)
EF091714, Sobemovirus, Ryegrass mottle virus
OR843475, TC-Solemo-LV-23 (233-17E/6271)
KC577469, Sobemovirus, Rottboellia yellow mottle virus
OR843480, TC-Solemo-LV-28 (233-17E/2579)
ON828429, Xufa yellow dwarf virus
JN620802, Sobemovirus, Artemisia virus A
OR843483, TC-Solemo-LV-31 (233-17E/3014)
OM954298, Flumine sobemo-like virus 39
OR843428, H-Solemo-LV-1 (644-18E/243)
Z48630, Sobemovirus, Cocksfoot Mottle Virus
OR843499, TC-Solemo-LV-47 (233-17E/19392)
OR843487, TC-Solemo-LV-35 (233-17E/8803)
AM990928, Sobemovirus, Imperata yellow mottle virus
AJ608206, Sobemovirus, Rice yellow mottle virus
JQ782213, Sobemovirus, Lucerne transient streak virus
AF208001, Sobemovirus, Subterranean clover mottle virus
JX123318, Sobemovirus, Papaya lethal yellowing virus
OR843478, TC-Solemo-LV-26 (233-17E/2335)
LC019764, Sobemovirus, Cymbidium chlorotic mosaic virus
HM754263, Sobemovirus, Velvet tobacco mottle virus
KC577470, Sobemovirus, Solanum nodiflorum mottle virus
MK6811451, Sobemovirus, Physalis rugose mosaic virus
LC081344, Sobemovirus, Blueberry shoestring virus
KC778720, Sobemovirus, Turnip rosette virus
DQ875594, Sobemovirus, Southern bean mosaic virus
OR843479, TC-Solemo-LV-27 (233-17E/2404)
AY004291, Sobemovirus, Sesbania mosaic virus
JF495127, Sobemovirus, Soybean yellow common mosaic virus
M23021, Sobemovirus, Southern cowpea mosaic virus
AM940437, Sobemovirus, Rubus chlorotic mottle virus
GQ845002, Sobemovirus, Sowbane mosaic virus
MK889164, Hubei sclerotinia RNA virus 1
OR843484, TC-Solemo-LV-32 (233-17E/3060)
OR843488, TC-Solemo-LV-36 (233-17E/3753)
OR843445, H-Solemo-LV-18 (644-18E/257)
KX882816, Changjiang sobemo-like virus 1
OR843511, TC-Solemo-LV-59 (233-17E/33157)
OR843486, TC-Solemo-LV-34 (233-17E/3271)
OR843446, H-Solemo-LV-19 (644-18E/298)
KX882902, Hubei sobemo-like virus 2
OR843497, TC-Solemo-LV-45 (233-17E/14465)
OR843444, H-Solemo-LV-17 (644-18E/182)
OR843468, TC-Solemo-LV-16 (233-17E/1526)
KX882796, Beihai sobemo-like virus 2
OR843477, TC-Solemo-LV-25 (233-17E/2042)
OR843435, H-Solemo-LV-8 (644-18E/208)
KX882894, Hubei sobemo-like virus 1
OR843482, TC-Solemo-LV-30 (233-17E/2702)
ON398503, Polemovirus, Poinsettia latent virus isolate PV-0629
LC577770, Polemovirus, Poinsettia latent virus isolate Eup
AJ867490, Polemovirus, Poinsettia latent virus isolate Germany
D00530, Polerovirus, Potato leafroll virus
AF235168, Polerovirus, Cereal yellow dwarf virus isolate RPS RPV Mex-1
L25299, Polerovirus, Cereal yellow dwarf virus isolate RPV NY
MN600000, unassigned species, Groundnut rosette assistor virus
AY695933, Polerovirus, Carrot red leaf virus
OR843456, TC-Solemo-LV-4 (233-17E/1266)
OM948842, Cucurbit aphid-borne yellows virus
AF473561, Polerovirus, Beet western yellows virus
AF352024, Polerovirus, Beet chlorosis virus
L04573, Enamovirus, Pea enation mosaic virus 1
KU297983, Enamovirus, Alfalfa enamovirus 1
HF679486, Enamovirus, Citrus vein enation virus
MT536978, Enamovirus, Grapevine enamovirus 1
Solemoviridae
MZ218202, Barnaviridae sp. isolate R29-k141_631786
OR843515, TC-Solemo-LV-63 (233-17E/19827)
U07551, Barnavirus, Mushroom bacilliform virus
barna-like RdRp (cd23184)
Barnaviridae
OR843465, TC-Solemo-LV-13 (233-17E/1447)
OR843438, H-Solemo-LV-11 (644-18E/433)
OR843464, TC-Solemo-LV-12 (233-17E/1440)
OR843434, H-Solemo-LV-7 (644-18D/2388)
OR843473, TC-Solemo-LV-21 (233-17D/4707)
OR843472, TC-Solemo-LV-20 (233-17E/1596)
OR843447, H-Solemo-LV-20 (644-18E/460)
OR843441, H-Solemo-LV-14 (644-18E/3735)
OR843471, TC-Solemo-LV-19 (233-17E/1581)
OR843448, H-Solemo-LV-21 (644-18E/696)
OR843452, H-Solemo-LV-25 (644-18E/262)
OR843451, H-Solemo-LV-24 (644-18E/144)
OR843455, TC-Solemo-LV-3 (233-17E/1105)
OR843490, TC-Solemo-LV-38 (233-17E/5960)
OR843491, TC-Solemo-LV-39 (233-17E/5961)
AB218608, Dinornavirus, Heterocapsa circularisquama RNA virus HcRNAV34
AB218609, Dinornavirus, Heterocapsa circularisquama RNA virus HcRNAV109
Alvernaviridae
KX882811, Beihai sobemo-like virus 6
KX882804, Beihai sobemo-like virus 7
OR843513, TC-Solemo-LV-61 (233-17E/1352)
OR843514, TC-Solemo-LV-62_233-17/2602)
Solemo-like clade B
with barna-like RdRp (cd23184)
KX882905, Hubei sobemo-like virus 4
KX882802, Beihai sobemo-like virus 5
OR843512, TC-Solemo-LV-60 (233-17E/34672)
Sobelivirales
X07653, Luteovirus, Barley yellow dwarf virus isolate PAV
AF218798, Luteovirus, Barley yellow dwarf virus isolate PAS PAV-129
EU024678, Luteovirus, Rose spring dwarf-associated virus
AF441393, Luteovirus, Bean leafroll virus
L24049, Luteovirus, Soybean dwarf virus
Tombusviridae
Tolivirales
2

Figure S5

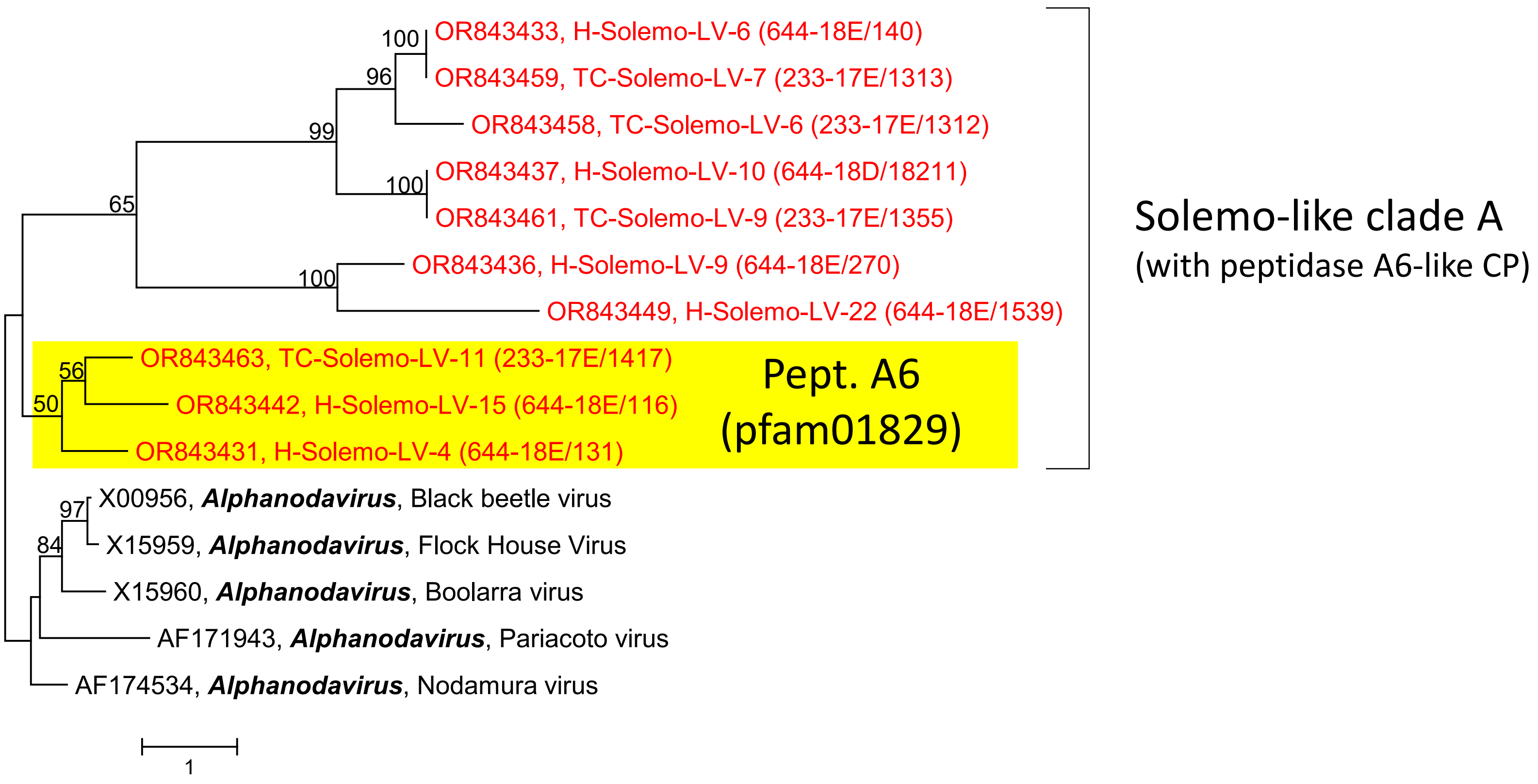

100
96
99
100
65
100
56
50
97
84
OR843433, H-Solemo-LV-6 (644-18E/140)
OR843459, TC-Solemo-LV-7 (233-17E/1313)
OR843458, TC-Solemo-LV-6 (233-17E/1312)
OR843437, H-Solemo-LV-10 (644-18D/18211)
OR843461, TC-Solemo-LV-9 (233-17E/1355)
OR843436, H-Solemo-LV-9 (644-18E/270)
OR843449, H-Solemo-LV-22 (644-18E/1539)
OR843463, TC-Solemo-LV-11 (233-17E/1417)
OR843442, H-Solemo-LV-15 (644-18E/116)
OR843431, H-Solemo-LV-4 (644-18E/131)
Pept. A6
(pfam01829)
Solemo-like clade A
(with peptidase A6-like CP)
X00956, Alphanodavirus, Black beetle virus
X15959, Alphanodavirus, Flock House Virus
X15960, Alphanodavirus, Boolarra virus
AF171943, Alphanodavirus, Pariacoto virus
AF174534, Alphanodavirus, Nodamura virus
1

Figure S6 A

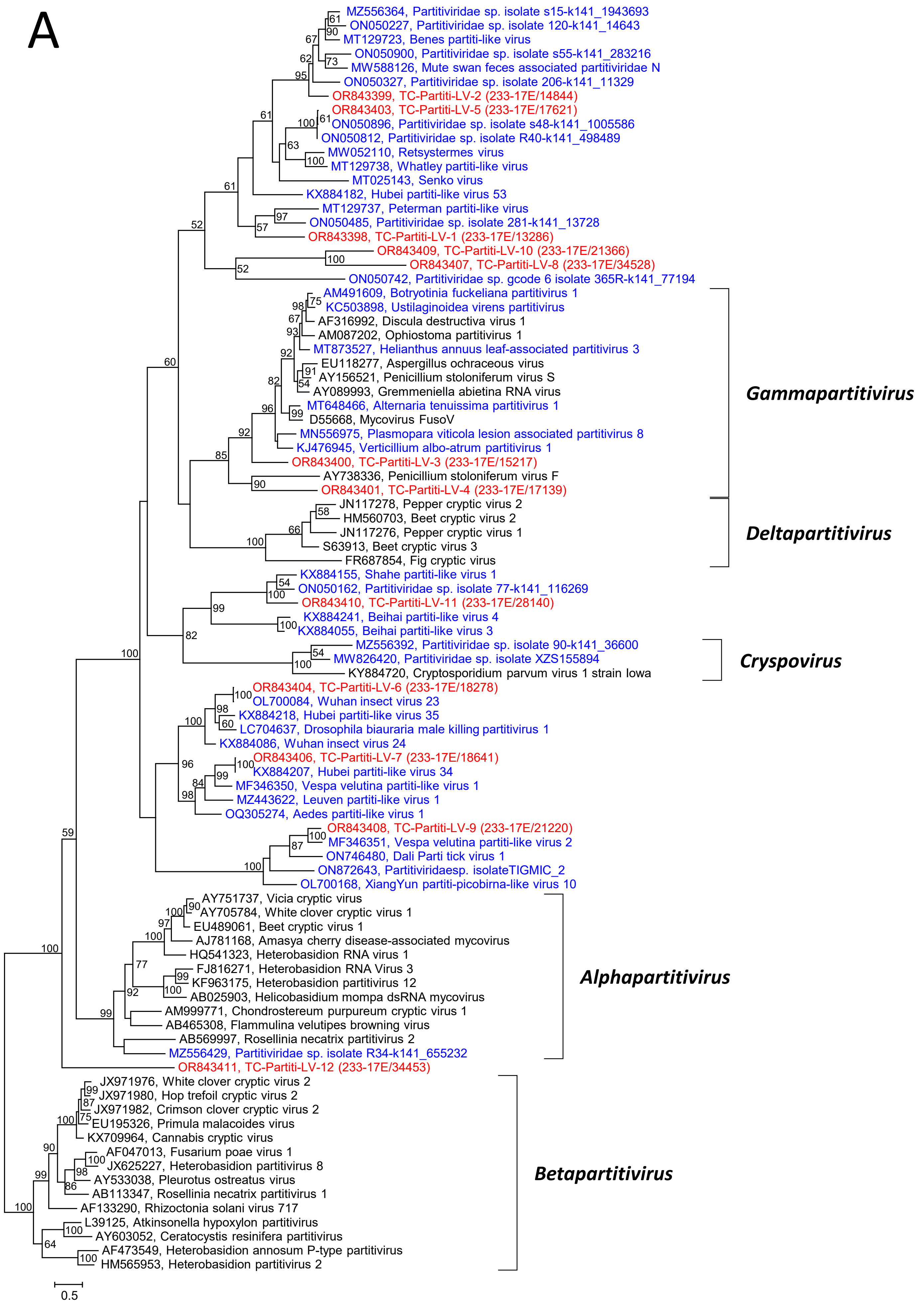
A
MZ556364, Partitiviridae sp. isolate s15-k141_1943693
ON050227, Partitiviridae sp. isolate 120-k141_14643
MT129723, Benes partiti-like virus
ON050900, Partitiviridae sp. isolate s55-k141_283216
MW588126, Mute swan feces associated partitiviridae N
ON050327, Partitiviridae sp. isolate 206-k141_11329
OR843399, TC-Partiti-LV-2 (233-17E/14844)
OR843403, TC-Partiti-LV-5 (233-17E/17621)
ON050896, Partitiviridae sp. isolate s48-k141_1005586
ON050812, Partitiviridae sp. isolate R40-k141_498489
MW052110, Retsystermes virus
MT129738, Whatley partiti-like virus
MT025143, Senko virus
KX884182, Hubei partiti-like virus 53
MT129737, Peterman partiti-like virus
ON050485, Partitiviridae sp. isolate 281-k141_13728
OR843398, TC-Partiti-LV-1 (233-17E/13286)
OR843409, TC-Partiti-LV-10 (233-17E/21366)
OR843407, TC-Partiti-LV-8 (233-17E/34528)
ON050742, Partitiviridae sp. gcode 6 isolate 365R-k141_77194
AM491609, Botryotinia fuckeliana partitivirus 1
KC503898, Ustilaginoidea virens partitivirus
AF316992, Discula destructiva virus 1
AM087202, Ophiostoma partitivirus 1
MT873527, Helianthus annuus leaf-associated partitivirus 3
EU118277, Aspergillus ochraceous virus
AY156521, Penicillium stoloniferum virus S
AY089993, Gremmeniella abietina RNA virus
MT648466, Alternaria tenuissima partitivirus 1
D55668, Mycovirus FusoV
MN556975, Plasmopara viticola lesion associated partitivirus 8
KJ476945, Verticillium albo-atrum partitivirus 1
OR843400, TC-Partiti-LV-3 (233-17E/15217)
AY738336, Penicillium stoloniferum virus F
OR843401, TC-Partiti-LV-4 (233-17E/17139)
Gammapartitivirus
JN117278, Pepper cryptic virus 2
HM560703, Beet cryptic virus 2
JN117276, Pepper cryptic virus 1
S63913, Beet cryptic virus 3
FR687854, Fig cryptic virus
Deltapartitivirus
KX884155, Shahe partiti-like virus 1
ON050162, Partitiviridae sp. isolate 77-k141_116269
OR843410, TC-Partiti-LV-11 (233-17E/28140)
KX884241, Beihai partiti-like virus 4
KX884055, Beihai partiti-like virus 3
MZ556392, Partitiviridae sp. isolate 90-k141_36600
MW826420, Partitiviridae sp. isolate XZS155894
KY884720, Cryptosporidium parvum virus 1 strain Iowa
Cryspovirus
OR843404, TC-Partiti-LV-6 (233-17E/18278)
OL700084, Wuhan insect virus 23
KX884218, Hubei partiti-like virus 35
LC704637, Drosophila biauraria male killing partitivirus 1
KX884086, Wuhan insect virus 24
OR843406, TC-Partiti-LV-7 (233-17E/18641)
KX884207, Hubei partiti-like virus 34
MF346350, Vespa velutina partiti-like virus 1
MZ443622, Leuven partiti-like virus 1
OQ305274, Aedes partiti-like virus 1
OR843408, TC-Partiti-LV-9 (233-17E/21220)
MF346351, Vespa velutina partiti-like virus 2
ON746480, Dali Parti tick virus 1
ON872643, Partitiviridaesp. isolateTIGMIC_2
OL700168, XiangYun partiti-picobirna-like virus 10
AY751737, Vicia cryptic virus
AY705784, White clover cryptic virus 1
EU489061, Beet cryptic virus 1
AJ781168, Amasya cherry disease-associated mycovirus
HQ541323, Heterobasidion RNA virus 1
FJ816271, Heterobasidion RNA Virus 3
KF963175, Heterobasidion partitivirus 12
AB025903, Helicobasidium mompa dsRNA mycovirus
AM999771, Chondrostereum purpureum cryptic virus 1
AB465308, Flammulina velutipes browning virus
AB569997, Rosellinia necatrix partitivirus 2
MZ556429, Partitiviridae sp. isolate R34-k141_655232
Alphapartitivirus
OR843411, TC-Partiti-LV-12 (233-17E/34453)
JX971976, White clover cryptic virus 2
JX971980, Hop trefoil cryptic virus 2
JX971982, Crimson clover cryptic virus 2
EU195326, Primula malacoides virus
KX709964, Cannabis cryptic virus
AF047013, Fusarium poae virus 1
JX625227, Heterobasidion partitivirus 8
AY533038, Pleurotus ostreatus virus
AB113347, Rosellinia necatrix partitivirus 1
AF133290, Rhizoctonia solani virus 717
L39125, Atkinsonella hypoxylon partitivirus
AY603052, Ceratocystis resinifera partitivirus
AF473549, Heterobasidion annosum P-type partitivirus
HM565953, Heterobasidion partitivirus 2
Betapartitivirus
0.5

Figure S6 B-D

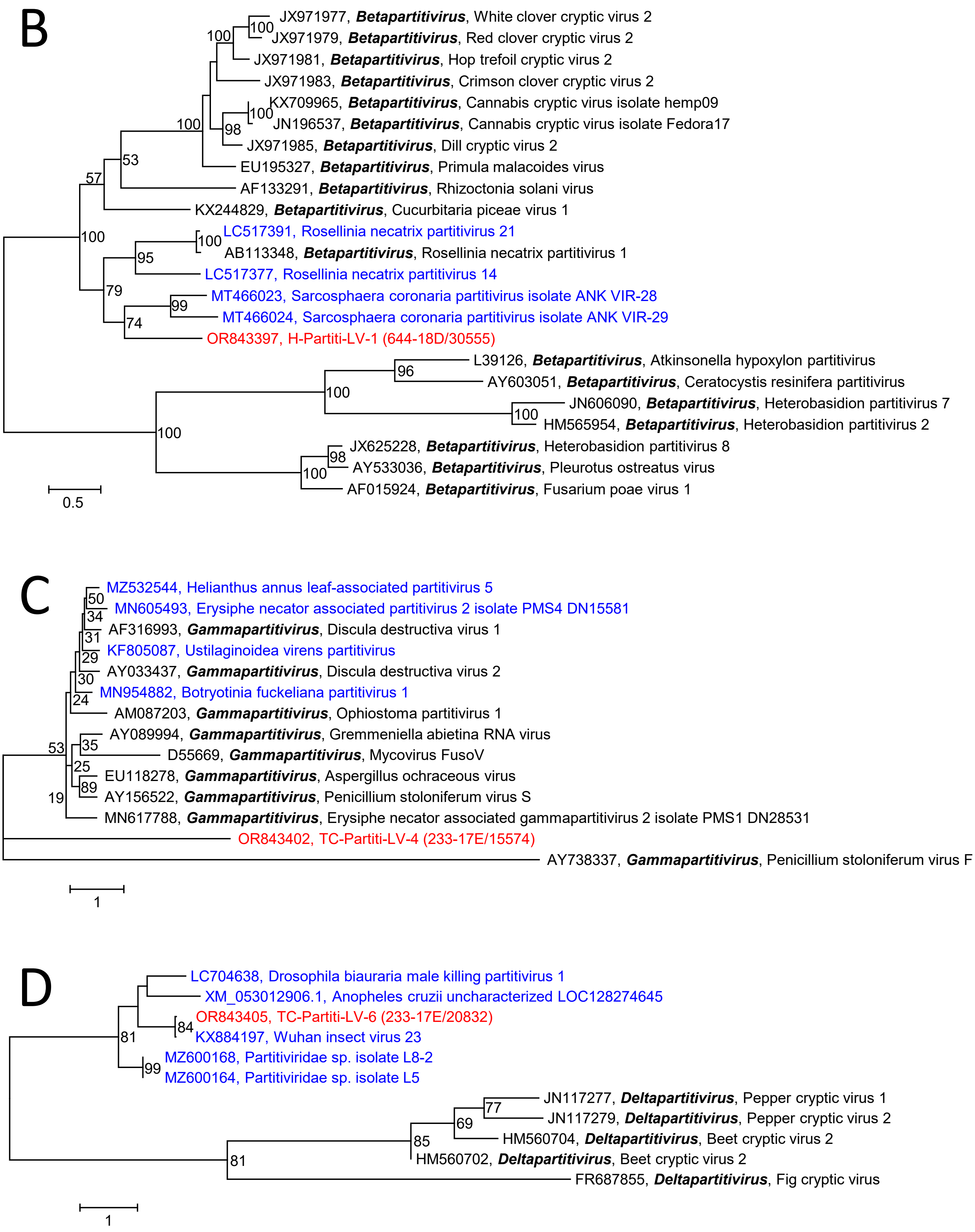
B
JX971977, Betapartitivirus, White clover cryptic virus 2
JX971979, Betapartitivirus, Red clover cryptic virus 2
JX971981, Betapartitivirus, Hop trefoil cryptic virus 2
JX971983, Betapartitivirus, Crimson clover cryptic virus 2
KX709965, Betapartitivirus, Cannabis cryptic virus isolate hemp09
JN196537, Betapartitivirus, Cannabis cryptic virus isolate Fedora17
JX971985, Betapartitivirus, Dill cryptic virus 2
EU195327, Betapartitivirus, Primula malacoides virus
AF133291, Betapartitivirus, Rhizoctonia solani virus
KX244829, Betapartitivirus, Cucurbitaria piceae virus 1
LC517391, Rosellinia necatrix partitivirus 21
AB113348, Betapartitivirus, Rosellinia necatrix partitivirus 1
LC517377, Rosellinia necatrix partitivirus 14
MT466023, Sarcosphaera coronaria partitivirus isolate ANK VIR-28
MT466024, Sarcosphaera coronaria partitivirus isolate ANK VIR-29
OR843397, H-Partiti-LV-1 (644-18D/30555)
L39126, Betapartitivirus, Atkinsonella hypoxylon partitivirus
AY603051, Betapartitivirus, Ceratocystis resinifera partitivirus
JN606090, Betapartitivirus, Heterobasidion partitivirus 7
HM565954, Betapartitivirus, Heterobasidion partitivirus 2
JX625228, Betapartitivirus, Heterobasidion partitivirus 8
AY533036, Betapartitivirus, Pleurotus ostreatus virus
AF015924, Betapartitivirus, Fusarium poae virus 1
0.5
C
MZ532544, Helianthus annus leaf-associated partitivirus 5
MN605493, Erysiphe necator associated partitivirus 2 isolate PMS4 DN15581
AF316993, Gammapartitivirus, Discula destructiva virus 1
KF805087, Ustilaginoidea virens partitivirus
AY033437, Gammapartitivirus, Discula destructiva virus 2
MN954882, Botryotinia fuckeliana partitivirus 1
AM087203, Gammapartitivirus, Ophiostoma partitivirus 1
AY089994, Gammapartitivirus, Gremmeniella abietina RNA virus
D55669, Gammapartitivirus, Mycovirus FusoV
EU118278, Gammapartitivirus, Aspergillus ochraceous virus
AY156522, Gammapartitivirus, Penicillium stoloniferum virus S
MN617788, Gammapartitivirus, Erysiphe necator associated gammapartitivirus 2 isolate PMS1 DN28531
OR843402, TC-Partiti-LV-4 (233-17E/15574)
AY738337, Gammapartitivirus, Penicillium stoloniferum virus F
1
D
LC704638, Drosophila biauraria male killing partitivirus 1
XM_053012906.1, Anopheles cruzii uncharacterized LOC128274645
OR843405, TC-Partiti-LV-6 (233-17E/20832)
KX884197, Wuhan insect virus 23
MZ600168, Partitiviridae sp. isolate L8-2
MZ600164, Partitiviridae sp. isolate L5
JN117277, Deltapartitivirus, Pepper cryptic virus 1
JN117279, Deltapartitivirus, Pepper cryptic virus 2
HM560704, Deltapartitivirus, Beet cryptic virus 2
HM560702, Deltapartitivirus, Beet cryptic virus 2
FR687855, Deltapartitivirus, Fig cryptic virus
1

Figure S7 A

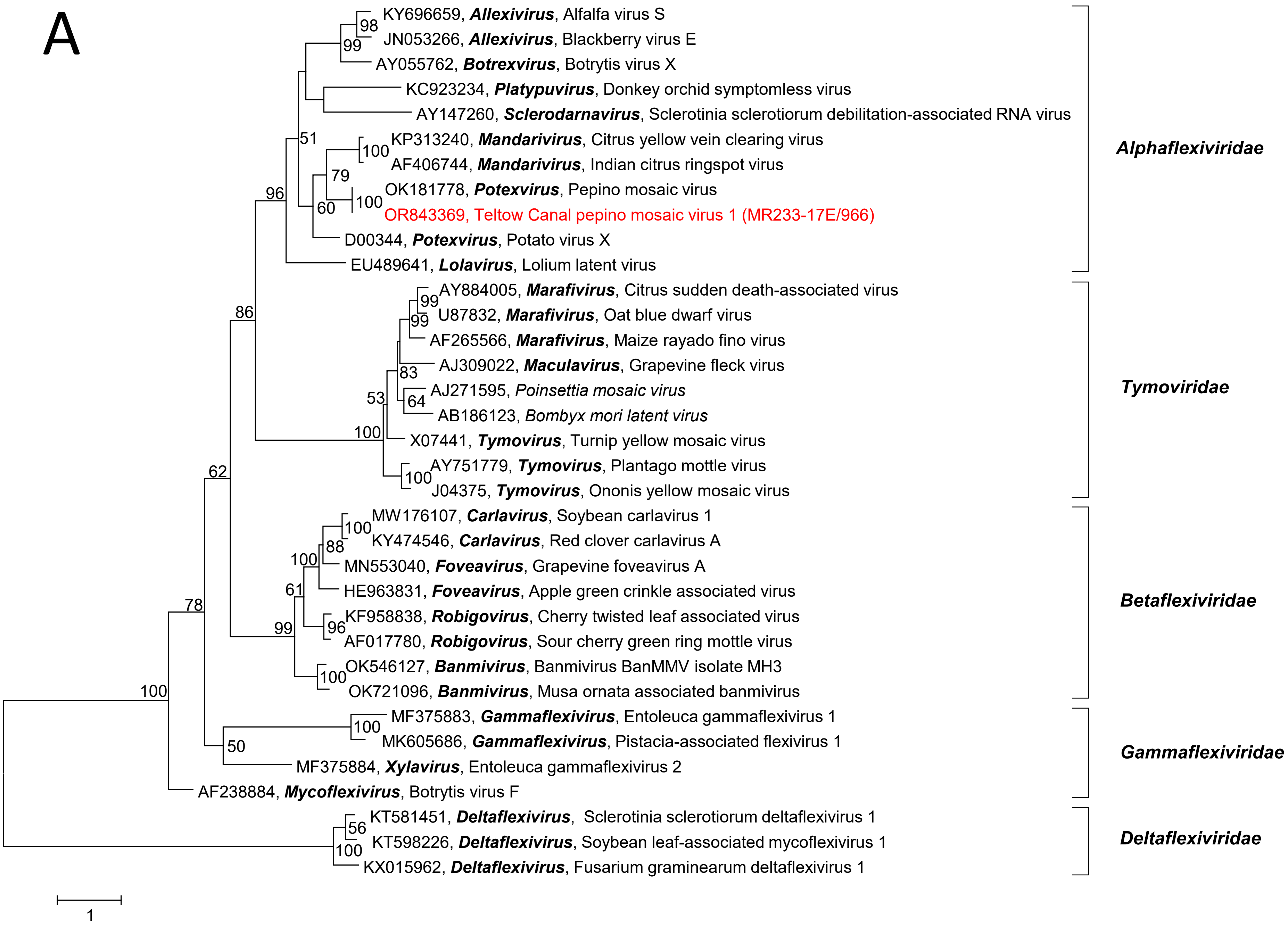
A
KY696659, Allexivirus, Alfalfa virus S
JN053266, Allexivirus, Blackberry virus E
AY055762, Botrexvirus, Botrytis virus X
KC923234, Platypuvirus, Donkey orchid symptomless virus
AY147260, Sclerodarnavirus, Sclerotinia sclerotiorum debilitation-associated RNA virus
KP313240, Mandarivirus, Citrus yellow vein clearing virus
AF406744, Mandarivirus, Indian citrus ringspot virus
OK181778, Potexvirus, Pepino mosaic virus
OR843369, Teltow Canal pepino mosaic virus 1 (MR233-17E/966)
D00344, Potexvirus, Potato virus X
EU489641, Lolavirus, Lolium latent virus
AY884005, Marafivirus, Citrus sudden death-associated virus
U87832, Marafivirus, Oat blue dwarf virus
AF265566, Marafivirus, Maize rayado fino virus
AJ309022, Maculavirus, Grapevine fleck virus
AJ271595, Poinsettia mosaic virus
AB186123, Bombyx mori latent virus
X07441, Tymovirus, Turnip yellow mosaic virus
AY751779, Tymovirus, Plantago mottle virus
J04375, Tymovirus, Ononis yellow mosaic virus
MW176107, Carlavirus, Soybean carlavirus 1
KY474546, Carlavirus, Red clover carlavirus A
MN553040, Foveavirus, Grapevine foveavirus A
HE963831, Foveavirus, Apple green crinkle associated virus
KF958838, Robigovirus, Cherry twisted leaf associated virus
AF017780, Robigovirus, Sour cherry green ring mottle virus
OK546127, Banmivirus, Banmivirus BanMMV isolate MH3
OK721096, Banmivirus, Musa ornata associated banmivirus
MF375883, Gammaflexivirus, Entoleuca gammaflexivirus 1
MK605686, Gammaflexivirus, Pistacia-associated flexivirus 1
MF375884, Xylavirus, Entoleuca gammaflexivirus 2
AF238884, Mycoflexivirus, Botrytis virus F
KT581451, Deltaflexivirus, Sclerotinia sclerotiorum deltaflexivirus 1
KT598226, Deltaflexivirus, Soybean leaf-associated mycoflexivirus 1
KX015962, Deltaflexivirus, Fusarium graminearum deltaflexivirus 1
Alphaflexiviridae
Tymoviridae
Betaflexiviridae
Gammaflexiviridae
Deltaflexiviridae
98
99
51
100
79
100
60
96
99
99
86
83
64
53
100
62
100
88
100
61
96
99
100
78
100
100
50
56
100
1

Figure S7 B

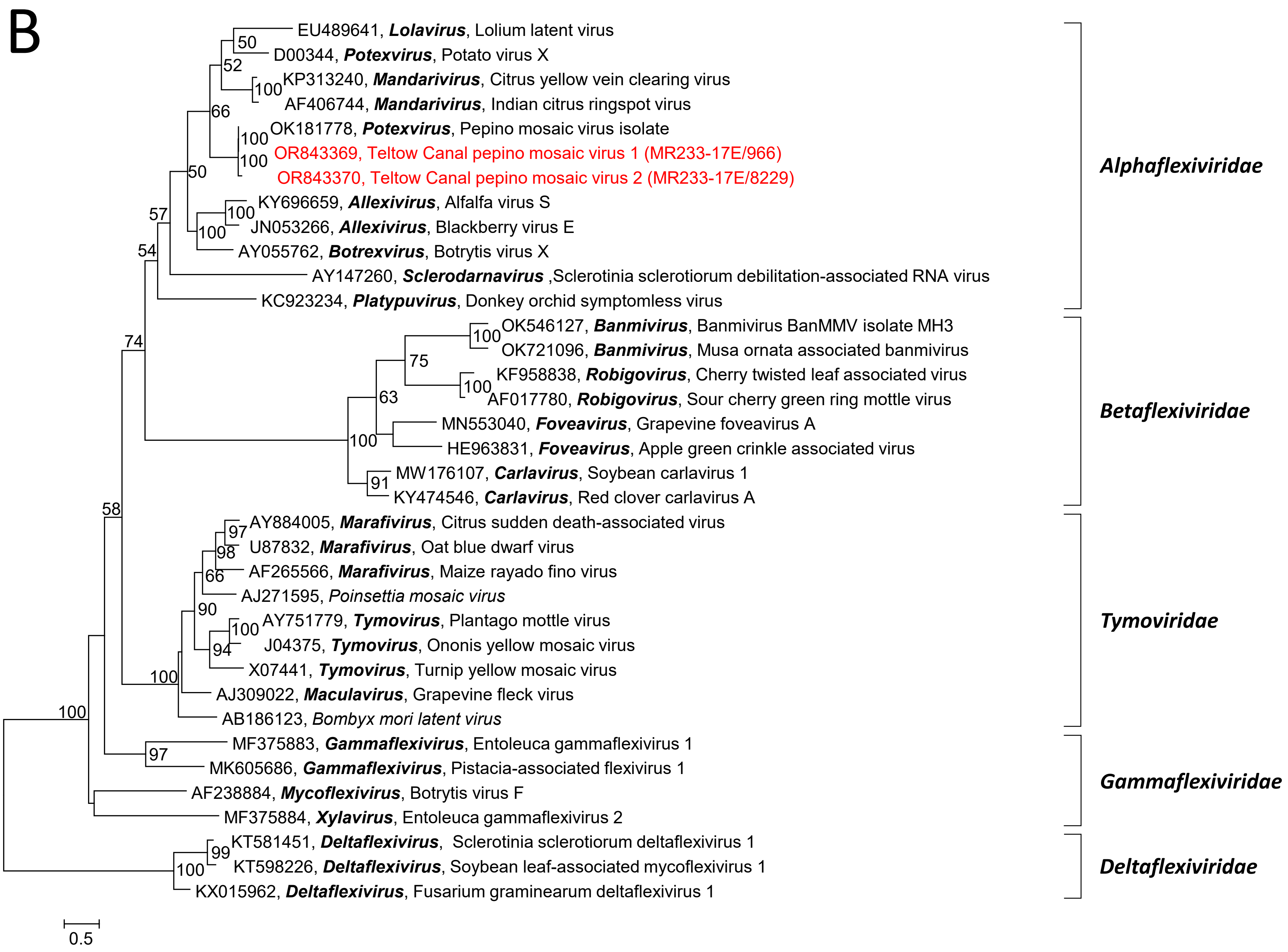
B
EU489641, Lolavirus, Lolium latent virus
D00344, Potexvirus, Potato virus X
KP313240, Mandarivirus, Citrus yellow vein clearing virus
AF406744, Mandarivirus, Indian citrus ringspot virus
OK181778, Potexvirus, Pepino mosaic virus isolate
OR843369, Teltow Canal pepino mosaic virus 1 (MR233-17E/966)
OR843370, Teltow Canal pepino mosaic virus 2 (MR233-17E/8229)
KY696659, Allexivirus, Alfalfa virus S
JN053266, Allexivirus, Blackberry virus E
AY055762, Botrexvirus, Botrytis virus X
AY147260, Sclerodarnavirus ,Sclerotinia sclerotiorum debilitation-associated RNA virus
KC923234, Platypuvirus, Donkey orchid symptomless virus
OK546127, Banmivirus, Banmivirus BanMMV isolate MH3
OK721096, Banmivirus, Musa ornata associated banmivirus
KF958838, Robigovirus, Cherry twisted leaf associated virus
AF017780, Robigovirus, Sour cherry green ring mottle virus
MN553040, Foveavirus, Grapevine foveavirus A
HE963831, Foveavirus, Apple green crinkle associated virus
MW176107, Carlavirus, Soybean carlavirus 1
KY474546, Carlavirus, Red clover carlavirus A
AY884005, Marafivirus, Citrus sudden death-associated virus
U87832, Marafivirus, Oat blue dwarf virus
AF265566, Marafivirus, Maize rayado fino virus
AJ271595, Poinsettia mosaic virus
AY751779, Tymovirus, Plantago mottle virus
J04375, Tymovirus, Ononis yellow mosaic virus
X07441, Tymovirus, Turnip yellow mosaic virus
AJ309022, Maculavirus, Grapevine fleck virus
AB186123, Bombyx mori latent virus
MF375883, Gammaflexivirus, Entoleuca gammaflexivirus 1
MK605686, Gammaflexivirus, Pistacia-associated flexivirus 1
AF238884, Mycoflexivirus, Botrytis virus F
MF375884, Xylavirus, Entoleuca gammaflexivirus 2
KT581451, Deltaflexivirus, Sclerotinia sclerotiorum deltaflexivirus 1
KT598226, Deltaflexivirus, Soybean leaf-associated mycoflexivirus 1
KX015962, Deltaflexivirus, Fusarium graminearum deltaflexivirus 1
Alphaflexiviridae
Betaflexiviridae
Tymoviridae
Gammaflexiviridae
Deltaflexiviridae
0.5

Figure S8

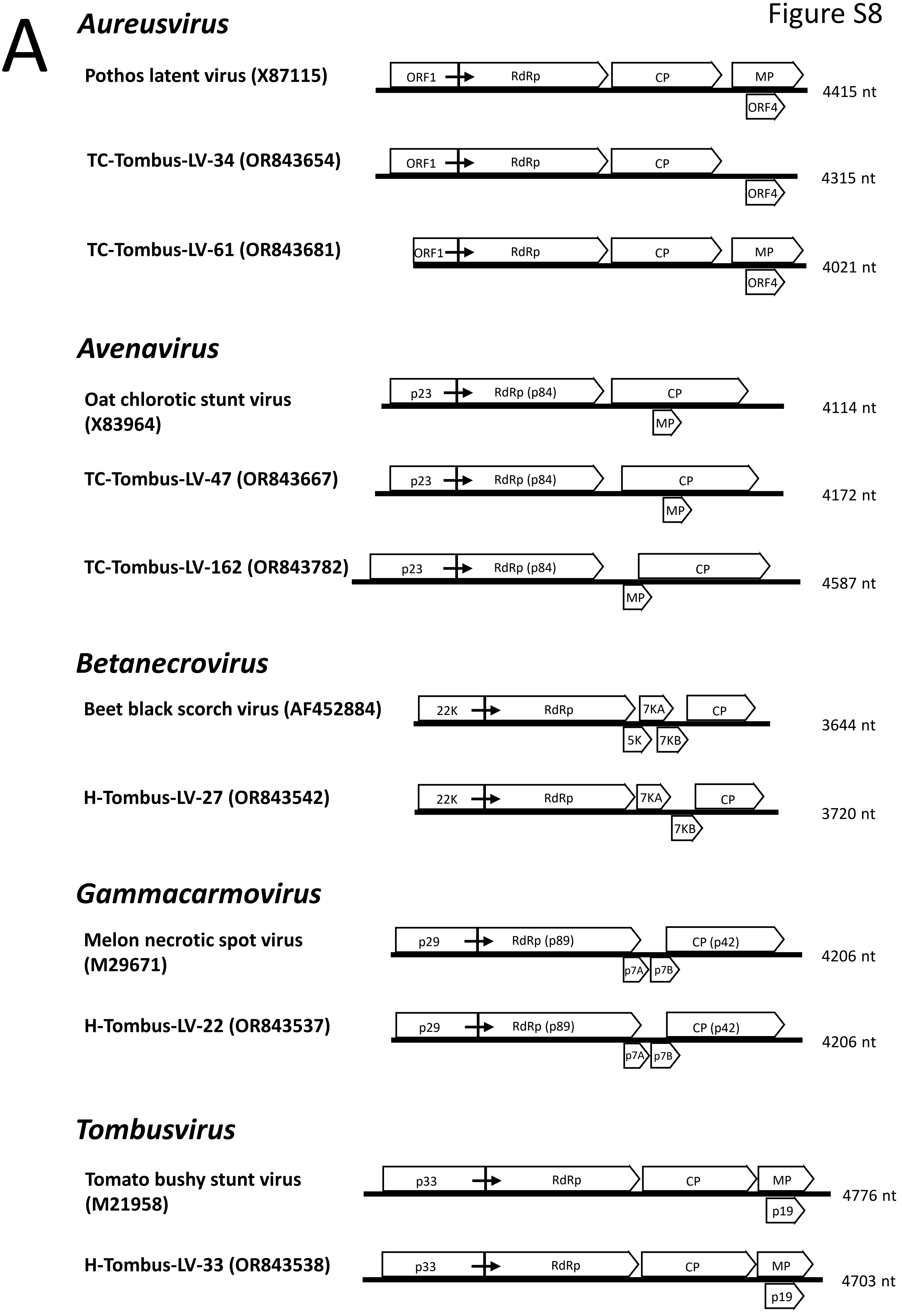
A
Aureusvirus
Pothos latent virus (X87115)
ORF1
RdRp
CP
MP
ORF4
4415 nt
TC-Tombus-LV-34 (OR843654)
ORF1
RdRp
CP
ORF4
4315 nt
TC-Tombus-LV-61 (OR843681)
ORF1
RdRp
CP
MP
ORF4
4021 nt
Avenavirus
Oat chlorotic stunt virus (X83964)
p23
RdRp (p84)
CP
MP
4114 nt
TC-Tombus-LV-47 (OR843667)
p23
RdRp (p84)
CP
MP
4172 nt
TC-Tombus-LV-162 (OR843782)
p23
RdRp (p84)
CP
MP
4587 nt
Betanecrovirus
Beet black scorch virus (AF452884)
22K
RdRp
7KA
CP
5K
7KB
3644 nt
H-Tombus-LV-27 (OR843542)
22K
RdRp
7KA
CP
7KB
3720 nt
Gammacarmovirus
Melon necrotic spot virus (M29671)
p29
RdRp (p89)
CP (p42)
p7A
p7B
4206 nt
H-Tombus-LV-22 (OR843537)
p29
RdRp (p89)
CP (p42)
p7A
p7B
4206 nt
Tombusvirus
Tomato bushy stunt virus (M21958)
p33
RdRp
CP
MP
p19
4776 nt
H-Tombus-LV-33 (OR843538)
p33
RdRp
CP
MP
p19
4703 nt

Figure S8 (contd.)

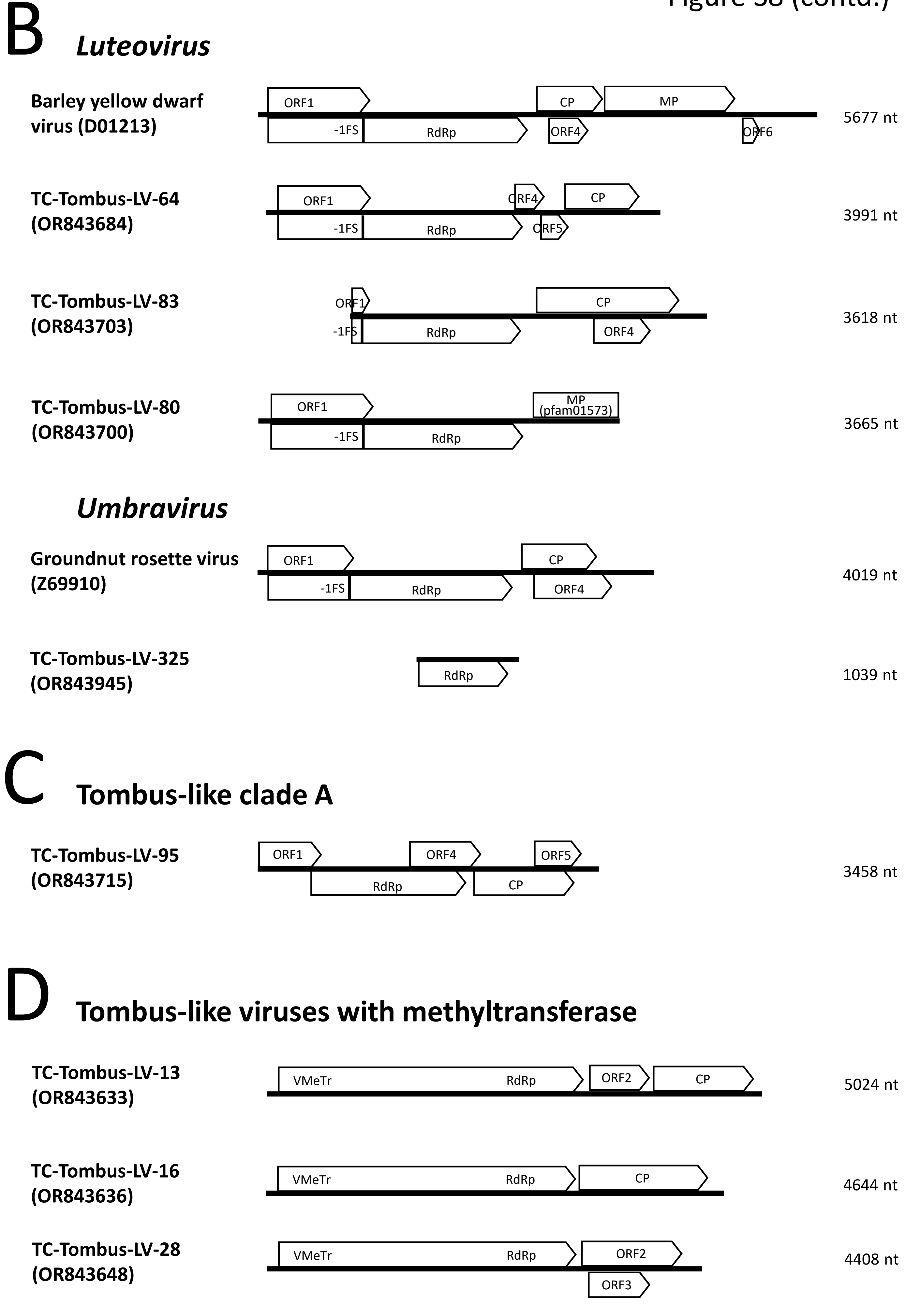

E **Solemo-like viruses**

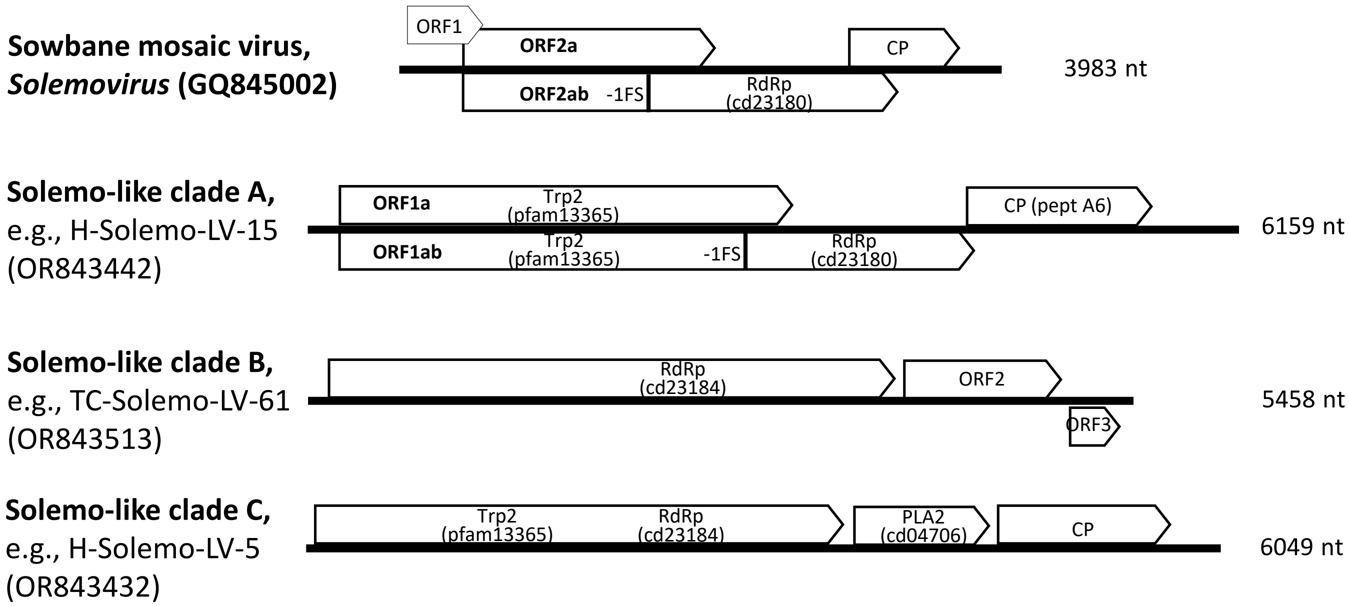

Supplementary Table							
**Related Genus/Family**	**Virus**	**Strain**	**Length**	**GenBank acc. no.**	**Coverage**	**Mapped reads**	**Comment**
Albetovirus	H-Albeto-LV-1	MR644-18E/2952	1312 nt	OR843342	16.3948	160	orf1: CP (pfam03898, cd00259); orf2-par
	H-Albeto-LV-2	MR644-18E/4748	1093 nt	OR843343	15.0192	120	orf-par: CP (pfam03898, cd00259)
	H-Albeto-LV-3	MR644-18E/7375	909 nt	OR843344	8.94939	58	orf: CP (pfam03898, cd00259)
	H-Albeto-LV-4	MR644-18E/23463	556 nt	OR843345	6.46583	30	orf-par: CP (pfam03898, cd00259)
	TC-Albeto-LV-1	MR233-17E/23417	1272 nt	OR843346	110.7660	998	UGA readthrough; orf: CP (pfam03898, cd00259)
	TC-Albeto-LV-2	MR233-17E/26183	1204 nt	OR843347	26.0399	224	orf-par: CP (pfam03898, cd00259)
	TC-Albeto-LV-3	MR233-17E/28086	1163 nt	OR843348	64.8126	548	orf-par: CP (pfam03898, cd00259)
	TC-Albeto-LV-4	MR233-17E/28659	1151 nt	OR843349	21.6429	178	orf: CP (pfam03898, cd00259)
	TC-Albeto-LV-5	MR233-17E/31420	1180 nt	OR843350	13.2941	112	orf-par: CP (pfam03898, cd00259)
	TC-Albeto-LV-6	MR233-17E/32763	1079 nt	OR843351	10.873	82	orf-par: CP (pfam03898, cd00259)
	TC-Albeto-LV-7	MR233-17E/33731	1064 nt	OR843352	13.2397	100	orf: CP (pfam03898, cd00259)
	TC-Albeto-LV-8	MR233-17E/41695	961 nt	OR843353	6.29136	44	orf: CP (pfam03898, cd00259)
	TC-Albeto-LV-9	MR233-17D/16470	1257 nt	OR843354	109.527	986	orf: CP (pfam03898, cd00259)
	TC-Albeto-LV-10	MR233-17D/4056	1220 nt	OR843355	39.4475	352	UGA readthrough; orf: CP (pfam03898, cd00259)
	TC-Albeto-LV-11	MR233-17D/85089	934 nt	OR843356	10.7398	70	orf: CP (pfam03898, cd00259)
	TC-Albeto-LV-12	MR233-17E/64990	763 nt	OR843357	3.82045	20	orf: CP (pfam03898, cd00259)
	TC-Albeto-LV-13	MR233-17D/384198	873 nt	OR843358	4.70218	30	orf: CP (pfam03898, cd00259)
	TC-Albeto-LV-14	MR233-17E/73956	711 nt	OR843359	5.68917	30	orf-par: CP (pfam03898, cd00259)
	TC-Albeto-LV-15	MR233-17E/22895	1897 nt	OR843360	188.807	2574	orf-par: CP (pfam03898, cd00259)
	TC-Albeto-LV-16	MR233-17E/88685	644 nt	OR843361	12.0373	56	orf-par: CP (pfam03898, cd00259)
	TC-Albeto-LV-17	MR233-17E/93272	627 nt	OR843362	6.6236	28	orf-par: CP (pfam03898, cd00259)
	TC-Albeto-LV-18	MR233-17D/327169	595 nt	OR843363	2.18487	10	orf-par: CP (pfam03898, cd00259)
	TC-Albeto-LV-19	MR233-17D/8875	1247 nt	OR843364	123.363	1112	orf-par: CP (pfam03898, cd00259)
	TC-Albeto-LV-20	MR233-17E/138842	506 nt	OR843365	2.31621	8	orf-par: CP (pfam03898, cd00259)
	TC-Albeto-LV-21	MR233-17E/135569	901 nt	OR843366	5.05882	30	orf: CP (pfam03898, cd00259)
	TC-Albeto-LV-22	MR233-17E/28400	1156 nt	OR843367	40.0882	334	orf: CP (pfam03898, cd00259)
	TC-Albeto-LV-23	MR233-17D/56545	730 nt	OR843368	3.60137	18	orf: CP (pfam03898, cd00259)
Alphaflexiviridae	Teltow Canal pepino mosaic virus 1	MR233-17E/966	6317 nt	OR843369	20.9242	936	orf1: VMethylTr (pfam01660), Hel1 (pfam01443), RdRp2 (pfam00978,cd23246); orf2: TRP1 (Hel1 pfam01443); orf3: TRP2 (MP pfam01307); orf4: TRP3 (pfam02495); orf5-par: CP (pfam00286)
	Teltow Canal pepino mosaic virus 2	MR233-17E/8229	2153 nt	OR843370	5.6828	86	orf1-par: Hel1 (pfam01443)
Aspiviridae	H-Aspi-LV-1	MR644-18D/11828	7284 nt	OR843371	23.4395	1234	RNA1; orf-par: RdRp (pfam00946)
	TC-Aspi-LV-1	MR233-17E/774	7082 nt	OR843372	24.2749	1248	RNA1; orf: RdRp (pfam00946)
	TC-Aspi-LV-2	MR233-17E/24594	1242 nt	OR843373	4.5709	38	RNA1; orf-par: RdRp (pfam00946)
	TC-Aspi-LV-3	MR233-17E/8303	2142 nt	OR843374	5.1881	82	RNA1; orf-par: RdRp (pfam00946)
Bromoviridae	TC-Bromo-LV-1	MR233-17E/20882	1344 nt	OR843375	33.7723	320	RNA3; orf: movement protein (pfam01573)
	TC-Bromo-LV-2	MR233-17E22280	1303 nt	OR843376	34.8734	330	RNA3; orf-par: movement protein (pfam01573)
Endornaviridae	TC-Endorna-LV-1	MR233-17E/63	15160 nt	OR843377	948.767	104848	orf1-par: RdRp2 (pfam00978, cd23255); orf2-par
	TC-Endorna-LV-2	MR233-17E/76	14026 nt	OR843378	76.1062	7712	orf-par: Hel1 (pfam01443), RdRp2 (pfam00978, cd23255)
	TC-Endorna-LV-3	MR233-17E/80	13469 nt	OR843379	29.2984	2878	orf-par: Hel1 (pfam01443), UDP-glycosyltransferase (cd03784, COG1819), RdRp2 (pfam00978, cd23255)
	TC-Endorna-LV-4	MR233-17E/81	13449 nt	OR843380	140.376	13534	orf-par: Hel1 (pfam01443), RdRp2 (pfam00978, cd23255)
	TC-Endorna-LV-5	MR233-17E/93	13085 nt	OR843381	23.2727	2198	orf-par: Hel1 (pfam01443), RdRp2 (pfam00978, cd23255)
	TC-Endorna-LV-6	MR233-17E/847	6769 nt	OR843382	52.3021	2526	suppression of UAG and UAA codons; orf-par: Hel1 (pfam01443)
	TC-Endorna-LV-7	MR233-17E/1061	6083 nt	OR843383	76.1093	3284	orf-par: RdRp2 (pfam00978, cd23255)
	TC-Endorna-LV-8	MR233-17E/1548	5088 nt	OR843384	12.2415	450	suppression of UAG and UAA codons; orf-par: Hel1 (pfam01443)
	TC-Endorna-LV-9	MR233-17E/1838	4676 nt	OR843385	12.2062	410	orf-par: Hel1 (pfam01443)
	TC-Endorna-LV-10	MR233-17E/1990	4480 nt	OR843386	30.3272	992	orf-par: RdRp2 (pfam00978, cd23255)
	TC-Endorna-LV-11	MR233-17E/2180	4283 nt	OR843387	20.834	638	suppression of UAG and UAA codons; orf-par
	TC-Endorna-LV-12	MR233-17E/2372	4091 nt	OR843388	29.4632	870	suppression of UAG and UAA codons; orf-par: RdRp2 (pfam00978, cd23255)
	TC-Endorna-LV-13	MR233-17E/2418	4059 nt	OR843389	121.735	3536	suppression of UAG and UAA codons + UGA readthrough; orf-par: RdRp2 (pfam00978, cd23255)
	TC-Endorna-LV-14	MR233-17E/4097	3124 nt	OR843390	14.3857	330	orf-par: Hel1 (pfam01443)
	TC-Endorna-LV-15	MR233-17E/4432	2984 nt	OR843391	33.51.91	716	suppression of UAG and UAA codons; orf-par: RdRp2 (pfam00978, cd23255)
	TC-Endorna-LV-16	MR233-17E/5308	2278 nt	OR843392	11.1173	218	orf-par: RdRp2 (pfam00978, cd23255)
	TC-Endorna-LV-17	MR233-17E/7446	2268 nt	OR843393	16.4101	270	orf-par: RdRp2 (pfam00978, cd23255)
	TC-Endorna-LV-18	MR233-17E/9238	2030 nt	OR843394	17.8906	262	orf-par: RdRp2 (pfam00978, cd23255)

	TC-Endorna-LV-19	MR233-17E/133	11565 nt	OR843395	24.6337	2020	suppression of UAG and UAA codons + UGA readthrough; orf-par: Hel1 (pfam01443), UDP-glycosyltransferase (cd03784, COG1819); RdRp2 (pfam00978, cd23255)
	TC-Endorna-LV-20	MR233-17E/206	10286 nt	OR843396	23.1245	1728	suppression of UAG and UAA codons; orf-par: UDP-glycosyltransferase (cd03784, COG1819), RdRp2 (pfam00978, cd23255)
Partitiviridae	H-Partiti-LV-1	MR644-18D/30555	1805 nt	OR843397	7.33407	94	dsRNA2; orf-par: CP
	TC-Partiti-LV1	MR233-17E/13286	1689 nt	OR843398	33.5565	430	dsRNA1; orf: RdRp1 (pfam00680, cd01699)
	TC-Partiti-LV-2	MR233-17E/14844	1595 nt	OR843399	47.4517	624	dsRNA1; orf: RdRp1 (pfam00680, cd01699)
	TC-Partiti-LV-3	MR233-17E/15217	1576 nt	OR843400	17.7747	204	dsRNA1; orf-par: RdRp1 (pfam00680, cd01699)
	TC-Partiti-LV-4	MR233-17E/17139	1485 nt	OR843401	9.96162	104	dsRNA1; orf-par: RdRp1 (pfam00680, cd01699)
		MR233-17E/15574	1559 nt	OR843402	17.771	206	dsRNA2; orf: CP
	TC-Partiti-LV-5	MR233-17E/17621	1465 nt	OR843403	142.902	1608	dsRNA1; orf: RdRp1 (pfam00680, cd01699)
	TC-Partiti-LV-6	MR233-17E/18278	1438 nt	OR843404	11.8039	118	dsRNA1; orf-par: RdRp1 (pfam00680, cd01699)
		MR233-17E/20832	1346 nt	OR843405	11.9354	118	dsRNA2; orf-par: CP
	TC-Partiti-LV-7	MR233-17E/18641	1424 nt	OR843406	22.4796	226	dsRNA1; orf: RdRp1 (pfam00680, cd01699)
	TC-Partiti-LV-8	MR233-17E/34528	1052 nt	OR843407	8.23954	64	dsRNA1; orf-par: RdRp1 (pfam00680, cd01699)
	TC-Partiti-LV-9	MR233-17E/21220	1334 nt	OR843408	41.4798	402	dsRNA1; orf: RdRp1 (pfam00680, cd01699)
	TC-Partiti-LV-10	MR233-17E/21366	1330 nt	OR843409	12.7053	120	dsRNA1; orf-par: RdRp1 (pfam00680, cd01699)
	TC-Partiti-LV-11	MR233-17E/28140	1162 nt	OR843410	11.6291	94	dsRNA1; orf-par: RdRp1 (pfam00680, cd01699)
	TC-Partiti-LV-12	MR233-17E/34453	1605 nt	OR843411	10.5768	116	dsRNA1; orf: RdRp1 (pfam00680, cd01699)
Potyviridae	TC-Poty-LV-1	MR233-17E/846	6770 nt	OR843412	81.3059	3918	orf1-par: prot (G$_{1033}$x**C**GxxxxxxxxxxxxxxxxGxH); orf2: RdRp1 (pfam00680, cd01699), CP (pfam00729)
	TC-Poty-LV-2	MR233-17E/1278	5912 nt	OR843413	11.9545	496	orf1-par: prot (G$_{837}$x**C**GxxxxxxxxxxxxGxH); orf2: RdRp1 (pfam00680, cd23175)
	TC-Poty-LV-3	MR233-17E/7275	2296 nt	OR843414	6.56228	110	orf1-par: RdRp1 (pfam00680, cd01699); orf2-par: CP (pfam00729); orf3
	TC-Poty-LV-4	MR233-17E/629	7838 nt	OR843415	2411.83	134754	orf1-par: trypsin 2 (pfam13365), RdRp1 (pfam00680, cd01699); orf2
	TC-Poty-LV-5	MR233-17E/706	7389 nt	OR843416	53.4595	2816	orf1-par: prot (G$_{1203}$x**C**GxxxxxxxxxxxxxxxxxxxxxxxxxxxxxGxH); orf2: RdRp1 (pfam00680, cd23175)
	TC-Poty-LV-6	MR233-17E/749	7140 nt	OR843417	39.6062	2020	orf1-par: prot? (G$_{942}$x**S**G); orf2: RdRp1 (pfam00680, cd23175), CP (pfam00729)
	TC-Poty-LV-7	MR233-17E/790	6983 nt	OR843418	31.0443	1546	orf1-par; orf2: RdRp1 (pfam00680, cd23175), CP (pfam00729)
	TC-Poty-LV-8	MR233-17E/798	6947 nt	OR843419	116.926	5832	orf1-par; orf2: RdRp1 (pfam00680, cd23175), CP (pfam00729)
	TC-Poty-LV-9	MR233-17E/794	6969 nt	OR843420	46.267	2310	orf1-par; orf2: RdRp1 (pfam00680, cd23175), CP (pfam00729)
	TC-Poty-LV-10	MR233-17E/860	6718 nt	OR843421	533.091	25662	orf1-par; orf2: RdRp1 (pfam00680, cd01699), CP (pfam00729)
	TC-Poty-LV-11	MR233-17E/915	6524 nt	OR843422	49.7784	2310	orf1-par; orf2: RdRp1 (pfam00680, cd23175), CP (pfam00729)
	TC-Poty-LV-12	MR233-17E/1300	5548 nt	OR843423	27.0319	1076	orf-par: RdRp1 (pfam00680, cd23175), CP (pfam00729)
	TC-Poty-LV-13	MR233-17E/1549	5082 nt	OR843424	16.06	584	orf1: Peptidase C4 (pfam00863); orf2-par: RdRp1 (pfam00680, cd23175), CP (pfam00729)
	TC-Poty-LV-14	MR233-17E/2352	7221 nt	OR843425	78.8446	4100	orf1-par; orf2: RdRp1 (pfam00680, cd23169), CP (pfam00729)
	TC-Poty-LV-15	MR233-17E/3501	4053 nt	OR843426	15.586	452	orf1-par: trypsin 2 (pfam13365); orf2: RdRp1 (pfam00680, cd23175); orf3
	TC-Poty-LV-16	MR233-17E/11552	2281 nt	OR843427	16.2679	262	orf1-par; orf2-par: RdRp1 (pfam00680, cd23175)
Solemoviridae	H-Solemo-LV-1	MR644-18E/243	4297 nt	OR843428	30.9367	968	orf1; orf2: Trypsin 2 (pfam13365); orf3: RdRp4 (pfam02123, cd23180); orf4: CP (pfam00729)
	H-Solemo-LV-2	MR644-18D/28050	5913 nt	OR843429	13.0908	554	orf1: Barnaviridae-RdRp (cd23184); orf2: parvo coat N (pfam08398); orf3-par: CP (pfam00729)
	H-Solemo-LV-3	MR644-18E/145	5532 nt	OR843430	63.9817	2596	orf2-par; orf3: RdRp4 (pfam02123, cd23180); orf4
	H-Solemo-LV-4	MR644-18E/131	5710 nt	OR843431	39.2278	1676	orf1: Trypsin 2 (pfam13365); orf2: RdRp4 (pfam02123, cd23180); orf3: Peptidase A6 (pfam01829)
	H-Solemo-LV-5	MR644-18E/121	6049 nt	OR843432	93.0665	4198	orf1: Trypsin 2 (pfam13365), Barnaviridae-RdRp (cd23184); orf2: phospholipase A2-like (cd04706); orf3: CP (pfam00729)
	H-Solemo-LV-6	MR644-18E/140	5584 nt	OR843433	186.344	7846	orf1: Peptidase S39 (pfam02122); orf2: RdRp4 (pfam02123, cd23180); orf3
	H-Solemo-LV-7	MR644-18D/2388	5554 nt	OR843434	94.7533	3816	orf1-par; orf2: RdRp4 (pfam02123, cd23180); orf3: CP (pfam00729)
	H-Solemo-LV-8	MR644-18E/208	4641 nt	OR843435	37.526	1254	orf1-par: Trypsin 2 (pfam13365); orf2: RdRp4 (pfam02123, cd23180); orf3: CP (pfam00729)
	H-Solemo-LV-9	MR644-18E/270	4135 nt	OR843436	10.9241	326	orf1-par: Trypsin 2 (pfam13365); orf2: RdRp4 (pfam02123, cd23180); orf3-par
	H-Solemo-LV-10	MR644-18D/18211	5376 nt	OR843437	63.4407	2462	orf1: Trypsin 2 (pfam13365); orf2: RdRp4 (pfam02123, cd23180); orf3
	H-Solemo-LV-11	MR644-18E/433	5386 nt	OR843438	147.962	5814	orf1-par; orf2: RdRp (cd23180); orf3: CP (pfam00729)
	H-Solemo-LV-12	MR644-18/541	2888 nt	OR843439	15.2961	316	orf-par: RdRp4 (pfam02123, cd23180), CP (pfam00729)
	H-Solemo-LV-13	MR644-18E/664	2571 nt	OR843440	12.942	244	orf1-par: Protease? (G$_{93}$x**S**GxxxxxxxxxxxxGxH), orf2-par: RdRp4 (pfam02123, cd23180)
	H-Solemo-LV-14	MR644-18E/3735	1195 nt	OR843441	4.9523	42	orf-par: RdRp4 (pfam02123, cd23180)

H-Solemo-LV-15	MR644-18E/116	6159 nt	OR843442	50.4269	2274	orf1: Trypsin 2 (pfam13365); orf2: RdRp4 (pfam02123, cd23180); orf3: Peptidase A6 (pfam01829)
H-Solemo-LV-16	MR644-18E/160	5245 nt	OR843443	227.527	8892	orf2-par; orf3: RdRp4 (pfam02123, cd23180); orf4
H-Solemo-LV-17	MR644-18E/182	4936 nt	OR843444	219.137	7830	orf1: Trypsin 2 (pfam13365); orf2: RdRp4 (pfam02123, cd23180); orf3: CP (pfam00729)
H-Solemo-LV-18	MR644-18E/257	4209 nt	OR843445	75.4688	2314	orf1; orf2: RdRp (cd23180); orf3
H-Solemo-LV-19	MR644-18E/298	3934 nt	OR843446	36.457	1042	orf1-par: Trypsin 2 (pfam13365); orf2: RdRp4 (pfam02123, cd23180); orf3
H-Solemo-LV-20	MR644-18E/460	3118 nt	OR843447	13.1947	296	orf1-par; orf2-par: RdRp (cd23180)
H-Solemo-LV-21	MR644-18D/30335	3258 nt	OR843448	9.76182	230	orf1-par; orf2: RdRp4 (pfam02123, cd23180); orf3-par: CP (pfam00729)
H-Solemo-LV-22	MR644-18E/1539	1709 nt	OR843449	9.05442	110	orf1-par: RdRp4 (pfam02123, cd23123); orf2-par
H-Solemo-LV-23	MR644-18E/2695	1356 nt	OR843450	10.1202	100	orf1: CP (pfam00729); orf2
H-Solemo-LV-24	MR644-18E/144	5542 nt	OR843451	50.5754	2062	orf1-par; orf2: RdRp (cd23180); orf3: CP (pfam00729)
H-Solemo-LV-25	MR644-18E/262	4184 nt	OR843452	11.1379	332	orf1-par; orf2: RdRp (cd23180); orf3: CP (pfam00729)
TC-Solemo-LV-1	MR233-17E/11830	5919 nt	OR843453	12.8332	540	orf1: Trypsin 2 (pfam13365), Barnaviridae-RdRp (cd23184); orf2; orf3: CP (pfam00729)
TC-Solemo-LV-2	MR233-17/263	5830 nt	OR843454	3620.59	150862	orf1-par: Barnaviridae-RdRp (cd23184); orf2: Parvo coat N (pfam08398); orf3: CP (pfam00729)
TC-Solemo-LV-3	MR233-17E/1105	5994 nt	OR843455	105.307	4464	orf1-par: Protease? (G834x**S**GxxxxxxxxxxxxGxH); orf2: RdRp4 (pfam02123, cd23180); orf3: CP (pfam00729)
TC-Solemo-LV-4	MR233-17E/1266	5632 nt	OR843456	16.6928	672	orf0-par: silencing repressor (pfam04662); orf1: Peptidase S39 (pfam02122); orf2: RdRp4 (pfam02123, cd23180); orf3: CP (pfam00894); orf3a: readthrough protein (pfam01690); orf4: MP (pfam01659),
TC-Solemo-LV-5	MR233-17E/1310	5537 nt	OR843457	1769.97	69788	orf1-par; orf2: RdRp4 (pfam02123, cd23180); orf3
TC-Solemo-LV-6	MR233-17E/1312	5533 nt	OR843458	1323.6	52394	orf1: Trypsin 2 (pfam13365); orf2: RdRp4 (pfam02123, cd23180); orf3
TC-Solemo-LV-7	MR233-17E/1313	5531 nt	OR843459	350.563	13852	orf1: Peptidase S39 (pfam02122); orf2: RdRp4 (pfam02123, cd23180); orf3
TC-Solemo-LV-8	MR233-17E/28299	1159 nt	OR843460	6.50733	56	orf-par: RdRp (cd23167)
TC-Solemo-LV-9	MR233-17E/1355	5453 nt	OR843461	29928.8	1165420	orf1: Trypsin 2 (pfam13365); orf2: RdRp4 (pfam02123, cd23180); orf3
TC-Solemo-LV-10	MR233-17E/1413	5332 nt	OR843462	579.733	21948	orf1: Protease? (G364x**S**GxxxxxxxxxxxxxxxGxH); orf2: RdRp4 (pfam02123, cd23180); orf3
TC-Solemo-LV-11	MR233-17E/1417	5318 nt	OR843463	25.9199	988	orf1-par: Trypsin 2 (pfam13365); orf2: RdRp4 (pfam02123, cd23180); orf3: Peptidase A6 (pfam01829)
TC-Solemo-LV-12	MR233-17D/27512	5545 nt	OR843464	54.4023	2152	orf1-par; orf2: RdRp1 (pfam00680, cd23180); orf3: CP (pfam00729)
TC-Solemo-LV-13	MR233-17D/12247	5540 nt	OR843465	51.0439	2020	orf1-par: Protease? (G542xSGxxxxxxxxxxxxxxGxH); orf2: RdRp1 (pfam00680, cd23180); orf3: CP (pfam00729)
TC-Solemo-LV-14	MR233-17E/1496	5173 nt	OR843466	246.722	9078	orf1-par: Protease? (G370x**S**GxxxxxxxxxxxxxxGxH); orf2: RdRp4 (pfam02123, cd23180); orf3
TC-Solemo-LV-15	MR233-17E/1520	5125 nt	OR843467	17.7982	648	orf1-par: Protease? (G489x**S**GxxxxxxxxxxxxxGxH); orf2-par: RdRp4 (pfam02123, cd23180)
TC-Solemo-LV-16	MR233-17E/1526	5110 nt	OR843468	102.269	3734	orf1: Trypsin 2 (pfam13365); orf2: RdRp4 (pfam02123, cd23180); orf3: CP (pfam00729)
TC-Solemo-LV-17	MR233-17E/1527	5109 nt	OR843469	17.2312	618	orf1-par; orf2: RdRp4 (pfam02123, cd23180); orf3
TC-Solemo-LV-18	MR233-17E/1569	5061 nt	OR843470	89.8295	3226	orf1; orf2: RdRp4 (pfam02123, cd23180); orf3
TC-Solemo-LV-19	MR233-17E/1581	5043 nt	OR843471	122.684	4434	orf1-par: Protease? (G518x**S**GxxxxxxxxxxxxxxGxhH); orf2: RdRp (cd23180); orf3: CP (pfam00729)
TC-Solemo-LV-20	MR233-17E/1596	5021 nt	OR843472	23.3989	1048	orf1-par; orf2: RdRp4 (pfam02123,cd23180); orf3: CP (pfam00729)
TC-Solemo-LV-21	MR233-17D/4707	5547 nt	OR843473	79.9751	3156	orf1-par: Protease? (G_{587}x**S**GxxxxxxxxxxxxxxGxH); orf2: RdRp4 (pfam02123, cd23180); orf3
TC-Solemo-LV-22	MR233-17E/6087	2523 nt	OR843474	12.9564	232	orf1-par; orf2-par: RdRp3 (pfam00998, cd23179)
TC-Solemo-LV-23	MR233-17E/6271	2485 nt	OR843475	15.6036	276	orf1-par: Peptidase S39 (pfam02122); orf2: RdRp4 (pfam02123, cd23180)
TC-Solemo-LV-24	MR233-17E/6795	2380 nt	OR843476	20.1672	346	orf1; orf2: Peptidase S39 (pfam02122); orf3-par: RdRp4 (pfam02123, cd23180)
TC-Solemo-LV-25	MR233-17E/2042	4427 nt	OR843477	41.7836	1312	orf1-par: Trypsin 2 (pfam13365); orf2: RdRp4 (pfam02123, cd23180); orf3: CP (pfam00729)
TC-Solemo-LV-26	MR233-17E/2335	4130 nt	OR843478	112.371	3310	orf1; orf2: Peptidase S39 (pfam02122); orf3: RdRp4 (pfam02123, cd23180); orf4: CP (pfam00729)
TC-Solemo-LV-27	MR233-17E/2404	4068 nt	OR843479	26.9299	778	orf1: Silencing suppressor; orf2: Peptidase S39 (pfam02122); orf3: RdRp4 (pfam02123, cd23180); orf4: CP (pfam00729)
TC-Solemo-LV-28	MR233-17/2579	4054 nt	OR843480	16.3389	474	orf1: Trypsin 2 (pfam13365), orf2: RdRp4 (pfam02123, cd23180); orf3: CP (pfam00729)
TC-Solemo-LV-29	MR233-17E/36496	1025 nt	OR843481	9.51805	70	orf1-par; orf2-par: RdRp4 (pfam02123, cd23180)

	TC-Solemo-LV-30	MR233-17E/2702	3847 nt	OR843482	577.937	15720	orf1-par: Peptidase S39 (pfam02122); orf2: RdRp4 (pfam02123, cd23180); orf3
	TC-Solemo-LV-31	MR233-17E/3014	4293 nt	OR843483	45.1372	1382	orf1; orf2: Trypsin 2 (pfam13365); orf3: RdRp4 (pfam02123, cd23180); orf4: CP (pfam00729)
	TC-Solemo-LV-32	MR233-17E/3060	3622 nt	OR843484	13.0555	334	orf1-par; orf2: Barnaviridae-RdRp (cd23184); orf3
	TC-Solemo-LV-33	MR233-17E/3193	3552 nt	OR843485	11.7424	298	orf1-par; orf2: RdRp4 (pfam02123, cd23180); orf3-par: CP (pfam00729)
	TC-Solemo-LV-34	MR233-17E/3271	4219 nt	OR843486	12.233	364	orf1-par: Trypsin 2 (pfam13365); orf2: RdRp4 (pfam02123, cd23180); orf3
	TC-Solemo-LV-35	MR233-17E/8803	2076 nt	OR843487	10.5048	154	orf1-par: RdRp4 (pfam02123, cd23180); orf2: CP (pfam00729)
	TC-Solemo-LV-36	MR233-17E/3753	3279 nt	OR843488	24.2897	566	orf1-par; orf2: Barnaviridae-RdRp (cd23184); orf3
	TC-Solemo-LV-37	MR233-17E/5389	2699 nt	OR843489	11.1423	216	orf1-par: RdRp1 (pfam00680, cd01699); orf2
	TC-Solemo-LV-38	MR233-17E/5960	3753 nt	OR843490	28.3893	764	orf1-par; orf2: RdR4 (pfam02123, cd23180)
	TC-Solemo-LV-39	MR233-17E/5961	2553 nt	OR843491	24.7932	448	orf-par: RdRp4 (pfam02123, cd23180)
	TC-Solemo-LV-40	MR233-17D/361	1710 nt	OR843492	5098.56	63036	orf: RdRp4 (pfam02123, cd23180)
	TC-Solemo-LV-41	MR233-17E/1715	4848 nt	OR843493	28.0243	962	orf1-par; orf2: RdRp1 (pfam00680, cd23180); orf3
	TC-Solemo-LV-42	MR233-17E/11509	1817 nt	OR843494	7.58008	98	orf-par: RdRp4 (pfam02123, cd23180), CP (pfam00729)
	TC-Solemo-LV-43	MR233-17E/6842	4014 nt	OR843495	213.603	6082	orf1-par: RdRp3 (pfam00998, cd23242); orf2: CP (pfam00729); orf3
	TC-Solemo-LV-44	MR233-17E/13244	1692 nt	OR843496	8.80095	104	orf1-par: Peptidase S39 (pfam02122); orf2-par
	TC-Solemo-LV-45	MR233-17E/14465	1616 nt	OR843497	5.64233	64	orf-par: RdRp4 (pfam02123, cd23180), CP (pfam00729)
	TC-Solemo-LV-46	MR233-17E/17111	1486 nt	OR843498	21.0868	224	orf1-par: CP (pfam00729); orf2
	TC-Solemo-LV-47	MR233-17E/19393	1396 nt	OR843499	16.8259	170	orf1-par: RdRp4 (pfam02123, cd23180); orf2-par: CP (pfam00729)
	TC-Solemo-LV-48	MR233-17D/2000	1359 nt	OR843500	250.399	2422	orf1: CP (pfam00729); orf2
	TC-Solemo-LV-49	MR233-17E/21734	1320 nt	OR843501	6.09924	56	orf: CP (pfam00729)
	TC-Solemo-LV-50	MR233-17E/22052	1310 nt	OR843502	17.1023	156	orf1-par: CP (pfam00729); orf2
	TC-Solemo-LV-51	MR233-17E/23527	1270 nt	OR843503	3.6189	32	orf1-par: RdRp4 (pfam02123, cd23180); orf2-par
	TC-Solemo-LV-52	MR233-17E/26654	1409 nt	OR843504	11.1679	110	orf-par: RdRp4 (pfam02123, cd23180)
	TC-Solemo-LV-53	MR233-17E/28631	1152 nt	OR843505	7.08767	56	orf-par: RdRp4 (pfam02123, cd23180)
	TC-Solemo-LV-54	MR233-17E/29307	1138 nt	OR843506	10.8884	86	orf-par: RdRp (c23180)
	TC-Solemo-LV-55	MR233-17E/29415	2350 nt	OR843507	5.63861	96	orf1-par: RdRp3 (pfam00998, cd23206); orf2: CP (pfam00729)
	TC-Solemo-LV-56	MR233-17E/29640	1132 nt	OR843508	8.61296	70	orf: CP (pfam00729)
	TC-Solemo-LV-57	MR233-17E/1122	1122 nt	OR843509	18.4376	148	orf: CP (pfam00729)
	TC-Solemo-LV-58	MR233-17E/30591	1115 nt	OR843510	12.9803	100	orf-par: Barnaviridae RdRp (cd23184)
	TC-Solemo-LV-59	MR233-17E/33157	1073 nt	OR843511	8.21994	62	orf-par: RdRp4 (pfam02123, cd23180)
	TC-Solemo-LV-60	MR233-17E/34672	1050 nt	OR843512	7.34	54	orf-par: RdRp4 (pfam02123, cd23180)
	TC-Solemo-LV-61	MR233-17E/1352	5454 nt	OR843513	14.8604	574	orf1: Barnaviridae RdRp (cd23184); orf2; orf3
	TC-Solemo-LV-62	MR233-17E/2602	5566 nt	OR843514	16.1985	632	orf1: Barnaviridae RdRp (cd23184); orf2; orf3
	TC-Solemo-LV-63	MR233-17E/19827	1380 nt	OR843515	6.64058	68	orf: Barnaviridae RdRp (cd23184)
Tombusviridae	H-Tombus-LV-1	MR644-18E/776	3628 nt	OR843516	231.065	6110	suppression of UAG and UAA codons, orf1-par; orf2: RdRp3 (pfam00998, cd23206); orf3: CP (pfam00729); orf4-par
	H-Tombus-LV-2	MR644-18E/176	5005 nt	OR843517	26.3447	956	orf1: VMethylTr (pfam01660), RdRp3 (pfam00998, cd01699); orf2: CP (pfam00729)
	H-Tombus-LV-3	MR644-18E/190	4848 nt	OR843518	79.4837	2762	orf1; orf2: RdRp3 (pfam00998, cd23179); orf3; orf4
	H-Tombus-LV-4	MR644-18E/210	4611 nt	OR843519	504.292	17142	orf1; orf2: RdRp3 (pfam00998, cd23179); orf3
	H-Tombus-LV-5	MR644-18E/214	4542 nt	OR843520	17.6449	576	orf1; orf2: ReRp3 (pfam00998, cd23179); orf3: CP (pfam00729)
	H-Tombus-LV-6	MR644-18E/225	4455 nt	OR843521	118.905	3996	orf1: P33 (pfam08500); orf2: RdRp3 (pfam00998, cd23242); orf3: PLA2 (cd04706), CP (pfam00729)
	H-Tombus-LV-7	MR644-18E/228	4431 nt	OR843522	23.2009	738	orf1; orf2: RdRp3 (pfam00998, cd23242); orf3: CP (pfam00729)
	H-Tombus-LV-8	MR644-18E/233	4389 nt	OR843523	816.977	26116	orf1; orf2: RdRp3 (pfam00998, cd23242); orf3: CP (pfam00729)
	H-Tombus-LV-9	MR644-18E/235	4381 nt	OR843524	30.3093	962	orf1; orf2: RdRp3 (pfam00998, cd23179); orf3: CP (pfam00729)
	H-Tombus-LV-10	MR644-18E/242	4319 nt	OR843525	58.2079	1816	orf1; orf2: RdRp3 (pfam00998, cd23179); orf3
	H-Tombus-LV-11	MR644-18E/245	4283 nt	OR843526	140.799	4342	orf1-par: P33 (pfam08500); orf2: RdRp3 (pfam00998, cd23242); orf3: CP (pfam00729)
	H-Tombus-LV-12	MR644-18E/248	4253 nt	OR843527	23.6906	728	orf1; orf2: RdRp3 (pfam00998, cd23239); orf3: CP (pfam00729)
	H-Tombus-LV-13	MR644-18E/251	4244 nt	OR843528	38.504	1186	orf1; orf2: RdRp3 (pfam00998, cd23242); orf3: CP (pfam00729)
	H-Tombus-LV-14	MR644-18E/259	4208 nt	OR843529	40.222	1256	orf1-par: P33 (pfam08500); orf2: RdRp3 (pfam00998, cd23242); orf3: PLA2 (cd04706), CP (pfam00729)
	H-Tombus-LV-15	MR644-18E/265	4162 nt	OR843530	38.8787	1244	orf1-par; orf2: RdRp3 (pfam00998, cd23242); orf3: CP (pfam00729)
	H-Tombus-LV-16	MR644-18E/266	4158 nt	OR843531	157.897	4774	orf1-par: Panicovirus-RdRp (cd23238); orf2: RdRp3 (pfam00998, cd23242); orf3: CP (pfam00729)
	H-Tombus-LV-17	MR644-18E/275	4081 nt	OR843532	36.5812	1102	orf1-par: P33 (pfam08500); orf2: RdRp3 (pfam00998, cd23242); orf3: PLA2 (cd04706), CP (pfam00729)

H-Tombus-LV-18	MR644-18E/284	4008 nt	OR843533	23.7625	694	orf1: P33 (pfam08500, cd23238); orf2: RdRp3 (pfam00998, cd23206); orf3: CP (pfam00729)
H-Tombus-LV-19	MR644-18E/285	4006 nt	OR843534	52.4201	1524	orf1-par; orf2: RdRp3 (pfam00998, cd23241); orf3: CP (pfam00729)
H-Tombus-LV-20	MR644-18E/286	3997 nt	OR843535	16.9039	488	orf1-par; orf2: RdRp3 (pfam00998, cd23179); orf3: CP (pfam00729)
H-Tombus-LV-21	MR644-18E/296	3948 nt	OR843536	27.0385	774	orf1-par; orf2: RdRp3 (pfam00998, cd23179); orf3: CP (pfam00729)
H-Tombus-LV-22	MR644-18E/300	3925 nt	OR843537	32.9386	938	orf1: Panicovirus-RdRp (cd23238); orf2: RdRp3 (pfam00998, cd23242); orf3: MP (pfam05318); orf4: CP (pfam00729)
H-Tombus-LV-23	MR644-18E/303	3909 nt	OR843538	33.3904	950	orf1-par, orf2: RdRp3 (pfam00998, cd23239); orf3
H-Tombus-LV-24	MR644-18E/323	3768 nt	OR843539	26.4639	752	orf1-par, orf2: RdRp3 (pfam00998, cd23242); orf3: CP (pfam00729)
H-Tombus-LV-25	MR644-18E/328	3739 nt	OR843540	3401.89	92598	orf1, orf2: RdRp3 (pfam00998, cd23242); orf3: CP (pfam00729)
H-Tombus-LV-26	MR644-18E/331	3735 nt	OR843541	41.2817	1120	suppression of UAG and UAA codons; orf1-par, orf2: RdRp3 (pfam00998, cd23206); orf3: CP (pfam00729)
H-Tombus-LV-27	MR644-18E/338	3720 nt	OR843542	411.815	11160	orf1-par: P33 (pfam08500); orf2: RdRp3 (pfam00998, cd23244); orf3; orf4; orf5
H-Tombus-LV-28	MR644-18E/342	3703 nt	OR843543	15.3408	418	orf1, orf2: RdRp3 (pfam00998, cd23242); orf3
H-Tombus-LV-29	MR644-18E/351	4097 nt	OR843544	33.575	966	orf1-par: P33 (pfam08500); orf2: RdRp3 (pfam00998, cd23206); orf3: CP (pfam00729)
H-Tombus-LV-30	MR644-18E/357	3604 nt	OR843545	21.5591	564	suppression of UAG and UAA codons, orf1; orf2: RdRp3 (pfam00998, cd23206); orf3: CP (pfam00729)
H-Tombus-LV-31	MR644-18E/362	5490 nt	OR843546	22.5811	898	orf1, orf2: RdRp3 (pfam00998, cd23242); orf3: CP (pfam00729)
H-Tombus-LV-32	MR644-18E/394	3459 nt	OR843547	25.2732	670	orf1: RdRp3 (pfam00998, cd23206); orf2: CP (pfam00729)
H-Tombus-LV-33	MR644-18E/429	4703 nt	OR843548	38.2492	1312	orf1: P33 (pfam08500); orf2: RdRp3 (pfam00998, cd23236); orf3: CP (pfam00729); orf4: P22/MP (pfam03558); orf5: P19/silencing suppressor protein (pfam03220)
H-Tombus-LV-34	MR644-18D/3709	3202 nt	OR843549	78.6324	1816	orf1-par; orf2: RdRp3 (pfam00998, cd23206); orf3
H-Tombus-LV-35	MR644-18E/506	4506 nt	OR843550	10.5202	344	orf1; orf2: RdRp (pfam00998, cd23179); orf3; orf4
H-Tombus-LV-36	MR644-18E/522	3570 nt	OR843551	20.1157	522	orf1-par; orf2: RdRp3 (pfam00998, cd23179); orf3; orf4
H-Tombus-LV-37	MR644-18E/542	2825 nt	OR843552	7.32035	152	orf1-par; orf2: RdRp (pfam00998, cd23179)
H-Tombus-LV-38	MR644-18E/586	2739 nt	OR843553	34.0661	682	orf1-par: RdRp (pfam00998, cd23242); orf2: PLA2 (cd04706), CP (pfam00729)
H-Tombus-LV-39	MR644-18E/593	2724 nt	OR843554	14.489	290	orf1-par; orf2: RdRp3 (pfam00998, cd23206); orf3-par
H-Tombus-LV-40	MR644-18E/642	2611 nt	OR843555	8.40061	156	orf1: p33 (pfam08500); orf2-par: RdRp (pfam00998, cd23242)
H-Tombus-LV-41	MR644-18E/710	2482 nt	OR843556	13.9363	254	orf1: RdRp (pfam00998, cd23206); orf2
H-Tombus-LV-42	MR644-18E/715	3537 nt	OR843557	3.78513	98	orf1-par; orf2: RdRp (pfam00998, cd23242); orf3
H-Tombus-LV-43	MR644-18E/864	2255 nt	OR843558	7.21818	116	orf1-par; orf2-par: RdRp3 (pfam00998, cd23206)
H-Tombus-LV-44	MR644-18E/1014	2092 nt	OR843559	13.1826	198	orf1-par; orf2: RdRp3 (pfam00998, cd23242)
H-Tombus-LV-45	MR644-18E/1044	2068 nt	OR843560	13.6654	204	orf1-par: RdRp3 (pfam00998, cd23206); orf2-par: Peptidase M3 (pfam01432)
H-Tombus-LV-46	MR644-18E/1090	2026 nt	OR843561	8.58144	128	orf1-par: Panicovirus-RdRp (pfam23238); orf2-par: RdRp3 (pfam00998, cd23206)
H-Tombus-LV-47	MR644-18D/24915	2058 nt	OR843562	16.2799	244	orf: RdRp3 (pfam00998, cd23242)
H-Tombus-LV-48	MR644-18E/1202	1939 nt	OR843563	8.9608	130	orf1-par: RdRp3 (pfam00998, cd23242); orf2
H-Tombus-LV-49	MR644-18E/1381	1804 nt	OR843564	12.1818	156	orf: RdRp3 (pfam00998, cd23241)
H-Tombus-LV-50	MR644-18E/1473	1756 nt	OR843565	6.9049	86	orf-par: RdRp3 (pfam00998, cd23179)
H-Tombus-LV-51	MR644-18E/1683	1644 nt	OR843566	7.90937	96	orf-par: RdRp3 (pfam00998, cd23242)
H-Tombus-LV-52	MR644-18E/1816	1590 nt	OR843567	5.12075	60	orf1-par: P33 (pfam08500); orf2-par: RdRp3 (pfam00998, cd23206)
H-Tombus-LV-53	MR644-18E/2394	1605 nt	OR843568	7.16885	84	orf1-par: RdRp3 (pfam00998, cd23206); orf2-par
H-Tombus-LV-54	MR644-18E/2840	1330 nt	OR843569	20.5624	200	orf-par: CP (pfam00729)
H-Tombus-LV-55	MR644-18E/3317	1388 nt	OR843570	10.3876	110	orf-par: RdRp3 (pfam00998, cd23206)
H-Tombus-LV-56	MR644-18E/4252	1142 nt	OR843571	10.0009	82	orf-par
H-Tombus-LV-57	MR644-18D/19461	4480 nt	OR843572	25.7656	864	orf1; orf2: RdRp3 (pfam00998, cd23241)
H-Tombus-LV-58	MR644-18E/359	3589 nt	OR843573	33.0666	868	suppression of UAG and UAA codons, orf1-par; orf2: RdRp3 (pfam00998, cd23206); orf3: CP (pfam00729);
H-Tombus-LV-59	MR644-18E/363	3567 nt	OR843574	104.635	2706	orf1-par: P33 (pfam08500); orf2: RdRp3 (pfam00998, cd23243); orf3: CP (pfam00729); orf4; orf5
H-Tombus-LV-60	MR644-18E/421	3321 nt	OR843575	15.9807	384	orf1; orf2: RdRp3 (pfam00998, cd23179)
H-Tombus-LV-61	MR644-18E/491	2960 nt	OR843576	29.4956	634	orf-par: VMethylTr (pfam01660), RdRp3 (pfam00998, cd23179)
H-Tombus-LV-62	MR644-18E/527	2872 nt	OR843577	102.256	2154	orf1; orf2: RdRp (cd23179)
H-Tombus-LV-63	MR644-18E/532	2860 nt	OR843578	20.1888	420	orf1-par; orf2-par: RdRp3 (pfam00998, cd01699)
H-Tombus-LV-64	MR644-18E/555	2797 nt	OR843579	11.1963	232	orf1-par: RdRp3 (pfam00998, cd23179); orf2
H-Tombus-LV-65	MR644-18E/564	2784 nt	OR843580	10.0295	200	orf1; orf2-par: RdRp3 (pfam00998, cd23179)
H-Tombus-LV-66	MR644-18E/978	2128 nt	OR843581	10.0498	152	orf-par: RdRp3 (pfam00998, cd23179)

H-Tombus-LV-67	MR644-18E/1331	2396 nt	OR843582	14.2684	248	orf1-par: RdRp3 (pfam00998, cd23179); orf2: CP (pfam00729)
H-Tombus-LV-68	MR644-18E/1477	1753 nt	OR843583	24.7296	322	orf1-par: Panicovirus-RdRp (cd23238); orf2-par: RdRp3 (pfam00998, cd23206)
H-Tombus-LV-69	MR644-18E/1675	1646 nt	OR843584	11.9034	140	orf: RdRp3 (pfam00998, cd23242)
H-Tombus-LV-70	MR644-18E/1703	1637 nt	OR843585	10.1179	124	orf1-par: RdRp3 (pfam00998, cd23239); orf2-par: CP (pfam00719)
H-Tombus-LV-71	MR644-18D/13390	1662 nt	OR843586	12.6679	156	orf: RdRp3 (pfam00998, cd23242)
H-Tombus-LV-72	MR644-18E/2085	1506 nt	OR843587	10.9934	122	orf-par: RdRp2 (pfam00978, cd01699)
H-Tombus-LV-73	MR644-18E/2179	1480 nt	OR843588	6.55135	70	orf-par: RdRp3 (pfam00998, cd23206)
H-Tombus-LV-74	MR644-18E/2210	1472 nt	OR843589	4.68886	52	orf1-par: RdRp3 (pfam00998, cd23206); orf2-par
H-Tombus-LV-75	MR644-18E/2225	1467 nt	OR843590	7.00341	76	orf-par: RdRp3 (pfam00998, cd23179)
H-Tombus-LV-76	MR644-18E/20890	1385 nt	OR843591	9.33405	92	orf-par: CP (pfam00729)
H-Tombus-LV-77	MR644-18E/2950	2664 nt	OR843592	12.914	258	orf1; orf2: RdRp3 (pfa,00998, cd23242)
H-Tombus-LV-78	MR644-18E/3051	1800 nt	OR843593	5.98278	78	orf1; orf2: RdRp3 (pfam00998, cd23179); orf3-par
H-Tombus-LV-79	MR644-18E/3199	1269 nt	OR843594	10.4027	92	orf1-par: RdRp3 (pfam00998, cd23179); orf2-par
H-Tombus-LV-80	MR644-18E/3256	1261 nt	OR843595	4.85408	44	orf1: RdRp (pfam00998, cd23238); orf2-par: PLA 2 (cd04706), CP (pfam00729)
H-Tombus-LV-81	MR644-18E/3474	1229 nt	OR843596	5.53377	48	orf1-par: RdRp3 (pfam00998, cd23242); orf2-par
H-Tombus-LV-82	MR644-18E/3654	1205 nt	OR843597	14.1867	124	orf1-par: P33 (pfam08500); orf2-par: RdRp3 (pfam00998, cd23242)
H-Tombus-LV-83	MR644-18D/54690	1439 nt	OR843598	5.9319	62	orf-par: RdRp3 (pfam00998, cd23179)
H-Tombus-LV-84	MR644-18E/3932	1174 nt	OR843599	36.2116	316	orf-par: CP (pfam00729)
H-Tombus-LV-85	MR644-18E/4150	1152 nt	OR843600	4.23351	36	orf1-par: CP (pfam00729); orf2-par: silencing suppressor protein (pfam19239)
H-Tombus-LV-86	MR644-18E/4228	1144 nt	OR843601	6.27972	50	orf1-par; orf2-par: RdRp3 (pfam00998, cd23242)
H-Tombus-LV-87	MR644-18E/4715	1096 nt	OR843602	11.7318	96	orf-par: CP (pfam00729)
H-Tombus-LV-88	MR644-18E/4859	1083 nt	OR843603	19.0905	92	suppression of UAG and UAA codons; orf-par: CP (pfam00729)
H-Tombus-LV-89	MR644-18E/1585	3291 nt	OR843604	9.0869	148	orf1-par; orf2-par: RdRp3 (pfam00998, cd23206)
H-Tombus-LV-90	MR644-18E/5288	1048 nt	OR843605	19.9485	222	orf-par: CP (pfam00729)
H-Tombus-LV-91	MR644-18E/5410	1039 nt	OR843606	9.08566	154	orf-par
H-Tombus-LV-92	MR644-18E/5898	1002 nt	OR843607	3.76946	66	orf1-par: RdRp3 (pfam00998, cd23242); orf2-par
H-Tombus-LV-93	MR644-18D/78707	873 nt	OR843608	8.49255	28	orf-par: RdRp3 (pfam00998, cd23243)
H-Tombus-LV-94	MR644-18E/373	3538 nt	OR843609	17.7337	52	suppression of UAG and UAA codons; orf1-par; orf2: RdRp3 (pfam00998, cd23206), orf3: CP (pfam00729)
H-Tombus-LV-95	MR644-18E/384	3490 nt	OR843610	32.1544	452	suppression of UAG and UAA codons; orf1; orf2: RdRp3 (pfam00998, cd23206); orf3: CP (pfam00729)
H-Tombus-LV-96	MR644-18E/389	3482 nt	OR843611	35.2823	812	suppression of UAG and UAA codons; orf1-par; orf2: RdRp3 (pfam00998, cd23206), orf3: CP (pfam00729)
H-Tombus-LV-97	MR644-18E/571	2769 nt	OR843612	18.1585	894	suppression of UAG and UAA codons; orf1-par; orf2: RdRp3 (pfam00998, cd23206), orf3-par: CP (pfam00729)
H-Tombus-LV-98	MR644-18D/18329	2602 nt	OR843613	16.0415	364	suppression of UAG and UAA codons; orf1-par: RdRp3 (pfam00998, cd23206), orf2: CP (pfam00729)
H-Tombus-LV-99	MR644-18E/22849	562 nt	OR843614	5.37011	296	orf-par: RdRp3 (pfam00998, cd23206)
H-Tombus-LV-100	MR644-18E/805	2346 nt	OR843615	12.3457	22	orf1-par; orf2: RdRp (cd23179)
H-Tombus-LV-101	MR644-18E/1214	2199 nt	OR843616	8.51387	214	orf1-par; orf2-par: RdRp (cd23179)
H-Tombus-LV-102	MR644-18E/1545	1706 nt	OR843617	10.2884	136	orf1-par; orf2-par: CP (pfam00729)
H-Tombus-LV-103	MR644-18E/2001	1529 nt	OR843618	31.6344	128	orf: CP (pfam00729)
H-Tombus-LV-104	MR644-18E/3839	1184 nt	OR843619	6.95524	62	orf1-par; orf2: RdRp3 (pfam00998, cd23244)
H-Tombus-LV-105	MR644-18E/5912	1001 nt	OR843620	4.99201	36	orf-par: CP (pfam00729)
TC-Tombus-LV-1	MR233-17E/8915	2760 nt	OR843621	38.9449	770	orf1; orf2: RdRp3 (pfam00998, cd23240); orf3
TC-Tombus-LV-2	MR233-17E/8986	2056 nt	OR843622	17.3234	250	orf1: RdRp3 (pfam00998, cd23179); orf2-par
TC-Tombus-LV-3	MR233-17D/20250	2236 nt	OR843623	34.4615	548	orf1-par: RdRp3 (pfam00998, cd23242); orf2: CP (pfam00729)
TC-Tombus-LV-4	MR233-17E/9475	2017 nt	OR843624	14.76	210	orf1-par: RdRp (cd23179); orf2: CP (pfam00729)
TC-Tombus-LV-5	MR233-17E/9475	2002 nt	OR843625	67.0772	1016	orfx; orf1; orf2-par: RdRp3 (pfam00998, cd23242)
TC-Tombus-LV-6	MR233-17D/10091	3455 nt	OR843626	66.2955	1644	suppression of UAG and UAA codons; orf1-par; orf2: RdRp3 (pfam0998, cd23206); orf3: CP (pfam00729); orf4-par
TC-Tombus-LV-7	MR233-17E/3079	3613 nt	OR843627	12.873	336	orf1: VMethylTr (pfam01660), RdRp2 (pfam00978, cd23179); orf2-par: CP (pfam00729)
TC-Tombus-LV-8	MR233-17E/807	6910 nt	OR843628	725.653	35746	orf1: RdRp1 (pfam00680, cd01699); orf2: CP (pfam00729)
TC-Tombus-LV-9	MR233-17E/1240	5678 nt	OR843629	26.0859	1058	orf1; orf2: RdRp (cd23179); orf3: Peptidase A21 (pfam03566)
TC-Tombus-LV-10	MR233-17E/1713	4851 nt	OR843630	1955.97	67772	orf1; orf2: RdRp3 (pfam00998, cd23179); orf3; orf4
TC-Tombus-LV-11	MR233-17E/1726	4832 nt	OR843631	91.1163	3144	orf1; orf2: RdRp3 (pdam00998, cd23179); orf3; orf4
TC-Tombus-LV-12	MR233-17E/1729	4829 nt	OR843632	1425.57	49166	orf1; orf2: RdRp3 (pfam00998, cd23179); orf3

TC-Tombus-LV-13	MR233-17E/1593	5024 nt	OR843633	35.3607	1266	orf1: VMethylTr (pfam01660), RdRp3 (pfam0998, cd23179); orf2; orf3: CP (pfam00729)
TC-Tombus-LV-14	MR233-17E/1701	4868 nt	OR843634	155,143	5352	orf1: RdRp3 (pfam0998, cd23179)
TC-Tombus-LV-15	MR233-17E/1765	4776 nt	OR843635	45.3185	1550	orf1-par; orf2: CBPV-like RdRp (cd23174)
TC-Tombus-LV-16	MR233-17E/1861	4644 nt	OR843636	72.2752	2390	orf1: VMethylTr (pfam01660), RdRp3 (pfam09988, cd23179); orf2: CP (pfam00729)
TC-Tombus-LV-17	MR233-17E/1924	4575 nt	OR843637	52.6424	1720	orf1; orf2: RdRp3 (pfam00998, cd23206); orf3: CP (pfam00729)
TC-Tombus-LV-18	MR233-17D/3535	4988 nt	OR843638	183.23	6558	orf1; orf2: RdRp3 (pfam00998, cd23179); orf3: CP (pfam00729)
TC-Tombus-LV-19	MR233-17E/1949	4537 nt	OR843639	82.8475	2688	orf-par: RdRp1 (pfam00680, cd01699)
TC-Tombus-LV-20	MR233-17E/1954	4533 nt	OR843640	80.3722	2602	orf1; orf2: RdRp3 (pfam00998, cd23206); orf3: CP (pfam00729)
TC-Tombus-LV-21	MR233-17E/1970	4516 nt	OR843641	15.0983	490	orf1; orf2: RdRp3 (pfam00998, cd23179), orf3
TC-Tombus-LV-22	MR233-17E/4509	4509 nt	OR843642	44.0189		orf1: VMethylTr (pfam01660), RdRp3 (pfam00998, cd23179), orf2: PLA2 (cd04706), CP (pfam00729)
TC-Tombus-LV-23	MR233-17E/2010	4456 nt	OR843643	118.136	3754	orf1; orf2: RdRp3 (pfam00998, cd23206); orf3: CP (pfam00729)
TC-Tombus-LV-24	MR233-17E/2024	4441 nt	OR843644	43.7976	1396	orf1: P33 (pfam08500); orf2: RdRp3 (pfam09988, cd23242); orf3: PLA2 (cd04706), CP (pfam00729)
TC-Tombus-LV-25	MR233-17E/2038	4429 nt	OR843645	20.9621	654	orf1-par; orf2: RdRp3 (pfam09988, cd23179); orf3-par
TC-Tombus-LV-26	MR233-17E/2056	4412 nt	OR843646	2585.88	80818	orf1; orf2: RdRp3 (pfam00998, cd23206); orf3: CP (pfam00729)
TC-Tombus-LV-27	MR233-17E/2060	4410 nt	OR843647	27.1474	866	orf1; orf2: RdRp3 (pfam00998, cd23179); orf3: CP (pfam00729)
TC-Tombus-LV-28	MR233-17E/2061	4408 nt	OR843648	17.801	560	orf1: VMethylTr (pfam01660), RdRp3 (pfam00998, cd23179); orf2
TC-Tombus-LV-29	MR233-17E/2073	4400 nt	OR843649	33.417	1050	orf1; orf2: RdRp3 (pfam00998, cd23241); orf3: PLA2 (cd04706), CP (pfam00729)
TC-Tombus-LV-30	MR233-17E/2084	4385 nt	OR843650	51.8951	1622	suppression of UAG and UAA codons; orf1-par: RdRp3 (pfam00998, cd23179); orf2: CP (pfam00729)
TC-Tombus-LV-31	MR233-17E/2120	4342 nt	OR843651	24.0661	740	orf1; orf2: RdRp3 (pfam00998, cd23239); orf3: CP (pfam00729)
TC-Tombus-LV-32	MR233-17E/2125	4338 nt	OR843652	19.503	612	orf1: P33 (pfam08500); orf2: RdRp3 (pfam00998, cd23238); orf3: PLA2 (cd04706), CP (pfam00729)
TC-Tombus-LV-33	MR233-17E/2133	4328 nt	OR843653	96.7442	2986	orf1; orf2: RdRp3 (pfam00998, cd23242); orf3: CP (pfam00729)
TC-Tombus-LV-34	MR233-17E/2145	4315 nt	OR843654	237.387	7294	orf1: P33 (pfam08500); orf2: RdRp3 (pfam00998, cd23243); orf3: CP (pfam00729); orf4
TC-Tombus-LV-35	MR233-17E/9436	2007 nt	OR843655	19.0169	274	orf1: Panicovirus-RdRp (cd23238); orf2: RdRp3 (pfam00998, cd23206)
TC-Tombus-LV-36	MR233-17E/2164	4300 nt	OR843656	33.8837	1032	orf1-par; orf2: RdRp3 (pfam00998, cd23179); orf3
TC-Tombus-LV-37	MR233-17E/2184	4280 nt	OR843657	13.8769	422	orf1; orf2: RdRp3 (pfam00998, cd23242); orf3: CP (pfam00729)
TC-Tombus-LV-38	MR233-17E/2195	4268 nt	OR843658	140.045	4248	orf1; orf2: RdRp3 (pfam00998, cd23179); orf3
TC-Tombus-LV-39	MR233-17E/2218	4247 nt	OR843659	179.283	5442	orf1: Panicovirus-RdRp (cd23238); orf2: RdRp3 (pfam00998, cd23242); orf3: CP (pfam00729)
TC-Tombus-LV-40	MR233-17E/2236	4227 nt	OR843660	991.116	30124	orf1; orf2: RdRp3 (pfam00998, cd23242); orf3: CP (pfam00729)
TC-Tombus-LV-41	MR233-17E/2238	4224 nt	OR843661	121.123	3660	orf1; orf2: RdRp3 (pfam00998, cd23206); orf3: CP (pfam00729)
TC-Tombus-LV-42	MR233-17E/2241	4220 nt	OR843662	42.1308	1276	orf1: Panicovirus-RdRp (cd23238); orf2: RdRp3 (pfam00998, cd23242); orf3: CP (pfam00729)
TC-Tombus-LV-43	MR233-17E/2257	4201 nt	OR843663	200.493	6054	orf1; orf2
TC-Tombus-LV-44	MR233-17E/2275	4182 nt	OR843664	16.1478	490	suppression of UAG and UAA codons; orf1-par: RdRp2 (pfam00978, cd01699); orf2: CP (pfam00729)
TC-Tombus-LV-45	MR233-17E/2279	4178 nt	OR843665	353.539	10440	orf1-par; orf2: RdRp3 (pfam00998, cd23206); orf3: CP (pfam00729)
TC-Tombus-LV-46	MR233-17E/2286	4176 nt	OR843666	18.2426	544	orf1-par: RdRp3 (pfam00998, cd01699); orf2-par: CP (pfam00729)
TC-Tombus-LV-47	MR233-17E/2291	4172 nt	OR843667	13.9456	416	orf1: P33 (pfam08500); orf2: RdRp3 (pfam00998, cd23242); orf3: CP (pfam00729)
TC-Tombus-LV-48	MR233-17E/2293	4171 nt	OR843668	66.3045	1954	orf1; orf2: RdRp3 (pfam00998, cd23179); orf3: CP (pfam00729)
TC-Tombus-LV-49	MR233-17E/2299	4164 nt	OR843669	34.4625	1016	orf1; orf2: RdRp3 (pfam00998, cd23242); orf3: CP (pfam00729)
TC-Tombus-LV-50	MR233-17E/2300	4163 nt	OR843670	212.074	6284	orf1-par: P33 (pfam08500); orf2: RdRp3 (pfam00998, cd23242); orf3: CP (pfam00729); orf4
TC-Tombus-LV-51	MR233-17E/2318	4145 nt	OR843671	147.754	4360	orf1; orf2: RdRp3 (pfam00998, cd23179); orf3: CP (pfam00729)
TC-Tombus-LV-52	MR233-17E/2326	4139 nt	OR843672	142.963	4240	orf1; orf2: RdRp3 (pfam00998, cd23206); orf3: CP (pfam00729)
TC-Tombus-LV-53	MR233-17E/2328	4137 nt	OR843673	17.7832	516	orf1; orf2: CBPV-RdRp (cd23174)
TC-Tombus-LV-54	MR233-17E/2345	4120 nt	OR843674	157.643	4614	orf1; orf2: RdRp3 (pfam00998, cd23206); orf3: CP (pfam00729)
TC-Tombus-LV-55	MR233-17E/2365	4096 nt	OR843675	25.552	742	orf1: P33 (pfam08500); orf2: RdRp3 (pfam00998, cd23242); orf3
TC-Tombus-LV-56	MR233-17E/2374	4089 nt	OR843676	16.8831	486	orf1-par; orf2: RdRp3 (pfam00998, cd23206); orf3: CP (pfam00729)
TC-Tombus-LV-57	MR233-17E/2385	4082 nt	OR843677	20918.6	608386	orf1-par; orf2: RdRp3 (pfam00998, cd23179); orf3: CP (pfam00729)
TC-Tombus-LV-58	MR233-17E/2413	4062 nt	OR843678	23.2937	674	orf1; orf2: RdRp3 (pfam00998, cd23242); orf3: CP (pfam00729)

TC-Tombus-LV-59	MR233-17E/2435	4042 nt	OR843679	56.9807	1642	orf1-par: P33 (pfam08500); orf2: RdRp3 (pfam00998, cd23242); orf3: CP (pfam00729)
TC-Tombus-LV-60	MR233-17E/2438	4040 nt	OR843680	31.4067	904	orf1; orf2: RdRp3 (pfam00998, cd23206); orf3: CP (pfam00729)
TC-Tombus-LV-61	MR233-17E/2466	4021 nt	OR843681	23.4628	674	orf1: P33 (pfam08500); orf2: RdRp3 (pfam00998,cd23243); orf3: CP (pfam00729); orf4: P22 (pfam03558)
TC-Tombus-LV-62	MR233-17E/2471	4017 nt	OR843682	20.6433	582	orf1; orf2: RdRp3 (pfam00998, cd23242); orf3: CP (pfam00729)
TC-Tombus-LV-63	MR233-17E/2497	3994 nt	OR843683	54.6557	1540	orf1-par; orf2: RdRp3 (cd23241); orf3: CP (pfam00729)
TC-Tombus-LV-64	MR233-17E/2502	3991 nt	OR843684	16.132	468	orf1; orf2: RdRp3 (pfam00998, cd23242); orf3: CP (pfam00729)
TC-Tombus-LV-65	MR233-17E/2510	3982 nt	OR843685	28.0771	798	orf1-par: P33 (pfam08500); orf2: RdRp3 (pfam00998, cd23242); orf3
TC-Tombus-LV-66	MR233-17D/4666	4211 nt	OR843686	69.2954	2086	orf1; orf2: RdRp3 (pfam00998, cd23242); orf3: CP (pfam00729)
TC-Tombus-LV-67	MR233-17E/2542	3954 nt	OR843687	22.0693	626	orf1-par: Panicovirus-RdRp (cd23238); orf2: RdRp3 (pfam00998, cd23206); orf3: CP (pfam00729)
TC-Tombus-LV-68	MR233-17E/2552	3949 nt	OR843688	88.7691	2516	orf1-par; orf2: RdRp3 (pfam00998, cd23179); orf3
TC-Tombus-LV-69	MR233-17E/2582	3929 nt	OR843689	28.5182	790	orf1; orf2: RdRp3 (pfam00998, cd23242); orf3: CP (pfam00729)
TC-Tombus-LV-70	MR233-17D/1252	4189 nt	OR843690	76.0208	2300	orf1; orf2: RdRp3 (pfam00998, cd23242); orf3: CP (pfam00729)
TC-Tombus-LV-71	MR233-17E/2625	3897 nt	OR843691	23.7259	664	orf1-par: Panicovirus-RdRp (cd23238); orf2: RdRp3 (pfam00998, cd23242); orf3: CP (pfam00729)
TC-Tombus-LV-72	MR233-17E/2663	3871 nt	OR843692	49.4368	1360	orf1-par: P33 (pfam08500); orf2: RdRp3 (pfam00998, cd23242); orf3: CP (pfam00729)
TC-Tombus-LV-73	MR233-17E/2665	3871 nt	OR843693	22.504	624	orf1-par: P33 (pfam08500); orf2: RdRp3 (pfam00998, cd23242); orf3: CP (pfam00729)
TC-Tombus-LV-74	MR233-17E/2704	3847 nt	OR843694	23.8505	656	orf1; orf2: RdRp3 (pfam00998, cd23242); orf3
TC-Tombus-LV-75	MR233-17E/2724	3829 nt	OR843695	31.695	858	orf1-par: P33 (pfam08500); orf2: RdRp3 (cd23239); orf3: CP (pfam00729)
TC-Tombus-LV-76	MR233-17E/2727	3828 nt	OR843696	25.61	686	orf1; orf2: RdRp3 (pfam00998, cd23242); orf3: CP (pfam00729)
TC-Tombus-LV-77	MR233-17E/2739	3822 nt	OR843697	19.9969	544	orf1-par; orf2: RdRp3 (pfam00998, cd23179); orf3: CP (pfam00729)
TC-Tombus-LV-78	MR233-17E/2882	3725 nt	OR843698	41.1487	1094	orf1: P33 (pfam08500); orf2: RdRp3 (pfam00998, cd23242); orf3: CP (pfam00729)
TC-Tombus-LV-79	MR233-17E/2941	3697 nt	OR843699	30.6833	800	orf1; orf2: RdRp3 (pfam00998, cd23206); orf3
TC-Tombus-LV-80	MR233-17E/2989	3665 nt	OR843700	13.743	358	orf1; orf2: RdRp (cd23233); orf3-par: MP (pfam01573)
TC-Tombus-LV-81	MR233-17E/3004	3659 nt	OR843701	22.9002	604	orf1-par; orf2: RdRp3 (pfam00998, cd23179); orf3
TC-Tombus-LV-82	MR233-17E/3033	3636 nt	OR843702	735.523	19118	orf1: Panicovirus-RdRp (cd23238); orf2: RdRp3 (pfam00998, cd23242); orf3: MP1 (pfam05318); orf4: MP2 (pfam06692); orf5-par: CP (pfam00729)
TC-Tombus-LV-83	MR233-17E/3068	3618 nt	OR843703	35.202	910	orf1-par; orf2: RdRp3 (pfam00998, cd23241); orf3: CP (pfam00729)
TC-Tombus-LV-84	MR233-17E/3086	3608 nt	OR843704	23.6014	610	orf1; orf2: RdRp3 (pfam00998, cd23206); orf3
TC-Tombus-LV-85	MR233-17E/3091	3606 nt	OR843705	199.647	5124	suppression of UAG and UAA codons; orf1-par; orf2: RdRp3 (pfam00998, cd23206); orf3: CP (pfam00729)
TC-Tombus-LV-86	MR233-17E/3106	3597 nt	OR843706	39.5076	1010	orf1: P33 (pfm08500); orf2: RdRp3 (pfam00998, cd23206); orf3
TC-Tombus-LV-87	MR233-17E/3122	3587 nt	OR843707	188.892	4804	suppression of UAG and UAA codons; orf1-par; orf2: RdRp3 (pfam00998, cd23206); orf3: CP (pfam00729)
TC-Tombus-LV-88	MR233-17E/3124	3587 nt	OR843708	26.4653	676	orf1: RdRp3 (pfam00998, cd23179); orf2: Peptidase A21 (pfam03566)
TC-Tombus-LV-89	MR233-17E/3246	3529 nt	OR843709	33.9974	856	orf1; orf2: RdRp3 (pfam00998, cd23179)
TC-Tombus-LV-90	MR233-17E/3270	3515 nt	OR843710	14.5807	364	orf1: P33 (pfam08500); orf2: RdRp3 (pfam00998, cd23206); orf3
TC-Tombus-LV-91	MR233-17E/3280	3507 nt	OR843711	79.8155	1998	orf1: P33 (pfam08500); orf2: RdRp3 (pfam00998, cd23206); orf3
TC-Tombus-LV-92	MR233-17E/3296	3497 nt	OR843712	24.0972	610	orf1; orf2: RdRp3 (pfam00998, cd23179); orf3
TC-Tombus-LV-93	MR233-17E/3303	3493 nt	OR843713	12.3856	308	orf1-par: RdRp3 (pfam00998, cd23237); orf2; orf3-par
TC-Tombus-LV-94	MR233-17E/3323	3480 nt	OR843714	22.9282	576	orf1-par: P33 (pfam08500); orf2: RdRp (cd23243); orf3: CP (pfam00729)
TC-Tombus-LV-95	MR233-17E/3376	3458 nt	OR843715	44.2964	1104	orf1-par: P33 (pfam08500); orf2: RdRp3 (pfam00998, cd23242); orf3: CP (pfam00729)
TC-Tombus-LV-96	MR233-17E/3403	3449 nt	OR843716	13.1806	318	orf1-par; orf2: RdRp3 (pfam00998, cd23206); orf3
TC-Tombus-LV-97	MR233-17E/3478	3411 nt	OR843717	15.3313	372	orf1-par; orf2-par: CP (pfam00729)
TC-Tombus-LV-98	MR233-17E/3511	3394 nt	OR843718	18.5854	462	orf1: Panicovirus-RdRp (cd23238); orf2: RdRp3 (pfam00998, cd23206); orf3
TC-Tombus-LV-99	MR233-17E/3532	3383 nt	OR843719	13.4478	322	orf1-par; orf2: RdRp3 (pfam00998, cd23237); orf3: CP (pfam00729); orf4
TC-Tombus-LV-100	MR233-17E/3551	3371 nt	OR843720	153.231	4878	orf1-par; orf2: RdRp3 (pfam00998, cd23241); orf3: CP (pfam00729)
TC-Tombus-LV-101	MR233-17E/3562	3365 nt	OR843721	13.9955	328	orf1-par; orf2: RdRp3 (pfam00998, cd23206); orf3: CP (pfam00729)
TC-Tombus-LV-102	MR233-17E/3580	3349 nt	OR843722	4143.25	94568	orf1; orf2: RdRp3 (pfam00998, cd23206); orf3
TC-Tombus-LV-103	MR233-17E/3591	3345 nt	OR843723	43.3229	1032	orf1-par: Panicovirus-RdRp (cd23238); orf2: RdRp3 (pfam00998, cd23242); orf3
TC-Tombus-LV-104	MR233-17E/3653	3320 nt	OR843724	28.775	678	orf1-par; orf2: RdRp3 (pfam00998, cd23206); orf3
TC-Tombus-LV-105	MR233-17E/3713	3296 nt	OR843725	10.034	236	orf1-par; orf2: RdRp3 (pfam00998, cd23179)

TC-Tombus-LV-106	MR233-17E/3742	3284 nt	OR843726	38.5253	900	orf1-par; orf2: RdRp3 (pfam00998, cd23206); orf3; orf4
TC-Tombus-LV-107	MR233-17E/3814	3248 nt	OR843727	105.969	2446	orf1: P33 (pfam08500); orf2: RdRp3 (pfam00998, cd23241); orf3; orf4-par
TC-Tombus-LV-108	MR233-17E/3849	3230 nt	OR843728	694.082	16036	orf1; orf2: RdRp3 (pfam00998, cd23179)
TC-Tombus-LV-109	MR233-17E/3857	3227 nt	OR843729	1054.12	24454	orf1; orf2: RdRp3 (pfam00998, cd23179)
TC-Tombus-LV-110	MR233-17E/3903	3208 nt	OR843730	59.6162	1846	orf1; orf2: RdRp3 (pfam00998, cd23242); orf3: CP (pfam00729)
TC-Tombus-LV-111	MR233-17E/3948	3186 nt	OR843731	64.9972	2006	orf1; orf2: RdRp3 (pfam00998, cd23242); orf3: CP (pfam00729)
TC-Tombus-LV-112	MR233-17E/3966	3178 nt	OR843732	37.5409	842	orf1-par: P33 (pfam08500); orf2: RdRp3 (pfam00998, cd23242); orf3
TC-Tombus-LV-113	MR233-17E/4271	3051 nt	OR843733	41.6317	1208	orf1-par: P33 (pfam08500); orf2: RdRp3 (pfam00998, cd23244); orf3: MP1 (pfam17493); orf4: MP2; orf5: CP (pfam00729)
TC-Tombus-LV-114	MR233-17E/4333	3019 nt	OR843734	5535.93	145204	orf1: ParB (pfam02195); orf2: RdRp3 (pfam00998, cd23179)
TC-Tombus-LV-115	MR233-17E/4395	3000 nt	OR843735	14.684	312	orf1-par: P33 (pfam08500); orf2: RdRp3 (pfam00998, cd23242); orf3: CP (pfam00729)
TC-Tombus-LV-116	MR233-17E/4425	2988 nt	OR843736	90.0977	1914	orf1-par: Panicovirus-RdRp (cd23238); orf2: RdRp3 (pfam00998, cd23241); orf3
TC-Tombus-LV-117	MR233-17E/4436	2983 nt	OR843737	253.643	5392	orf1-par: Panicovirus-RdRp (cd23238); orf2: RdRp3 (pfam00998, cd23241); orf3: CP (pfam00729)
TC-Tombus-LV-118	MR233-17E/4442	2982 nt	OR843738	10.3667	242	orf1; orf2: RdRp3 (pfam00998, cd23241); orf3-par: CP (pfam00729)
TC-Tombus-LV-119	MR233-17E/4454	2977 nt	OR843739	17.3863	368	orf-par: VMethylTr (pfam01660), RdRp3 (pfam00998, cd23179)
TC-Tombus-LV-120	MR233-17E/4468	2972 nt	OR843740	141.761	3004	suppression of UAG and UAA codons; orf1-par: RdRp3 (pfam00998, cd23206); orf2: CP (pfam00729)
TC-Tombus-LV-121	MR233-17E/4545	2941 nt	OR843741	35.4352	748	orf1: Panicovirus-RdRp (cd23238); orf2: RdRp3 (pfam00998, cd23240); orf3
TC-Tombus-LV-122	MR233-17E/4567	2934 nt	OR843742	47.3473	984	orf1-par; orf2: RdRp3 (pfam00998, cd23179); orf3-par: CP (pfam00729)
TC-Tombus-LV-123	MR233-17E/4573	2932 nt	OR843743	21.8663	456	orf1-par; orf2: RdRp3 (pfam00998, cd23242); orf3-par: CP (pfam00729)
TC-Tombus-LV-124	MR233-17E/4628	2916 nt	OR843744	150.824	3134	orf1-par; orf2: RdRp3 (pfam00998, cd23242); orf3: CP (pfam00729)
TC-Tombus-LV-125	MR233-17E/4634	2912 nt	OR843745	67.4681	1400	orf1-par; orf2: RdRp3 (pfam00998, cd23241); orf3
TC-Tombus-LV-126	MR233-17E/4652	2906 nt	OR843746	133.734	2768	orf1-par; orf2: RdRp3 (pfam00998, cd23242); orf3: CP (pfam00729)
TC-Tombus-LV-127	MR233-17E/4682	2894 nt	OR843747	127.313	2644	orf1-par; orf2: RdRp3 (pfam00998, cd23239); orf3; orf4-par
TC-Tombus-LV-128	MR233-17E/4701	2887 nt	OR843748	17.682	362	orf1-par; orf2: RdRp3 (pfam00998, cd23242); orf3: CP (pfam00729)
TC-Tombus-LV-129	MR233-17E/4712	2882 nt	OR843749	18.432	382	orf1-par: RdRp3 (pfam00998, cd23206); orf2: CP (pfam00729)
TC-Tombus-LV-130	MR233-17D/6096	2958 nt	OR843750	32.1018	678	suppression of UAG and UAA codons; orf1-par: RdRp3 (pfam00998, cd23206); orf2: CP (pfam00729)
TC-Tombus-LV-131	MR233-17E/4740	2871 nt	OR843751	39.3152	806	orf1-par; orf2: RdRp3 (pfam00998, cd23240); orf3-par
TC-Tombus-LV-132	MR233-17E/4770	2861 nt	OR843752	28.8724	582	orf1-par; orf2: RdRp3 (pfam00998, cd23240); orf3
TC-Tombus-LV-133	MR233-17E/4812	2852 nt	OR843753	350.341	8872	suppression of UAG and UAA codons; orf1-par; orf2: RdRp3 (pfam00998, cd23206); orf3: CP (pfam00729)
TC-Tombus-LV-134	MR233-17E/4821	2848 nt	OR843754	530.532	10730	orf1-par: P33 (pfam08500); orf2: RdRp3 (pfam00998, cd23242); orf3
TC-Tombus-LV-135	MR233-17E/4828	2846 nt	OR843755	283.855	5726	orf1-par; orf2: RdRp3 (pfam00998, cd23242); orf3
TC-Tombus-LV-136	MR233-17E/4839	2843 nt	OR843756	30.3345	618	orf1; orf2; orf3-par: RdRp3 (pfam00998, cd23206)
TC-Tombus-LV-137	MR233-17E/4906	2826 nt	OR843757	152.048	3050	orf1: P33 (pfam08500); orf2: RdRp3 (pfam00998, cd23242); orf3: CP (pfam00729)
TC-Tombus-LV-138	MR233-17E/4932	2818 nt	OR843758	50.1838	998	orf1: P33 (pfam08500); orf2: RdRp3 (pfam00998, cd23240); orf3: CP (pfam00729)
TC-Tombus-LV-139	MR233-17E/4948	2813 nt	OR843759	1472.17	38374	orf1: ParB (pfam02195); orf2: RdRp3 (pfam00998, cd23179)
TC-Tombus-LV-140	MR233-17E/4951	2813 nt	OR843760	13.3957	268	orf1-par; orf2: RdRp3 (pfam00998, cd23242); orf3: CP (pfam00729)
TC-Tombus-LV-141	MR233-17E/4965	2809 nt	OR843761	19.7156	396	orf1-par; orf2: RdRp1 (pfam00680, cd23174)
TC-Tombus-LV-142	MR233-17E/4967	2808 nt	OR843762	34.7714	696	orf1-par: RdRp3 (pfam00998, cd23206); orf2: CP (pfam00729)
TC-Tombus-LV-143	MR233-17E/5032	2789 nt	OR843763	32.8021	652	orf1-par: P33 (pfam08500); orf2: RdRp3 (pfam00998, cd23240); orf3: CP (pfam00729)
TC-Tombus-LV-144	MR233-17E/5085	2774 nt	OR843764	30.9211	608	orf1; orf2: RdRp3 (pfam00998, cd23179)
TC-Tombus-LV-145	MR233-17E/5164	2754 nt	OR843765	13.8021	274	orf1; orf2: CBPV-RdRp (cd23174)
TC-Tombus-LV-146	MR233-17E/5202	2743 nt	OR843766	17.3358	356	orf1-par; orf2: RdRp3 (pfam00998, cd23206); orf3
TC-Tombus-LV-147	MR233-17E/5213	3830 nt	OR843767	119.424	3264	orf1-par; orf2: RdRp3 (pfam00998, cd23206); orf3: CP (pfam00729)
TC-Tombus-LV-148	MR233-17E/5352	2708 nt	OR843768	14.0798	274	orf1-par: RdRp3 (pfam00998, cd23206); orf2: CP (pfam00729)
TC-Tombus-LV-149	MR233-17E/9898	3547 nt	OR843769	49.3045	1254	orf0-par; orf1; orf2: RdRp3 (pfam00998, cd23206); orf3-par
TC-Tombus-LV-150	MR233-17E/5470	2676 nt	OR843770	764.984	14550	orf1-par; orf2: RdRp3 (pfam00998, cd23179)
TC-Tombus-LV-151	MR233-17E/5518	2662 nt	OR843771	95.9574	3006	orf1; orf2: RdRp3 (pfam00998, cd23206); orf3: CP (pfam00729)
TC-Tombus-LV-152	MR233-17E/5562	2650 nt	OR843772	13.9389	266	orf1-par: RdRp3 (pfam00998, cd23206); orf2
TC-Tombus-LV-153	MR233-17E/5662	2624 nt	OR843773	26.1598	704	orf1: Panicovirus-RdRp (cd23238); orf2: RdRp3 (pfam00998, cd23242); orf3: MP/p7A (pfam05318); orf4; orf5: CP (pfam00729)

TC-Tombus-LV-154	MR233-17E/5707	2613 nt	OR843774	11.9399	224	orf1-par: P33 (pfam08500); orf2: RdRp3 (pfam00998, cd23206)
TC-Tombus-LV-155	MR233-17E/14189	4178 nt	OR843775	23.7645	700	orf1: P33 (pfam08500); orf2: RdRp3 (pfam00998, cd23242); orf3: CP (pfam00729)
TC-Tombus-LV-156	MR233-17E/5746	2604 nt	OR843776	8.08909	154	orf1-par; orf2: RdRp3 (pfam00998, cd23242)
TC-Tombus-LV-157	MR233-17E/5754	2601 nt	OR843777	12.3779	248	orf1-par: RdRp3 (pfam00998, cd23179); orf2: CP (pfam00729)
TC-Tombus-LV-158	MR233-17E/5758	4221 nt	OR843778	11.2507	336	orf1; orf2: RdRp3 (pfam00998, cd23242); orf3: PLA2 (cd04706), CP (pfam00729)
TC-Tombus-LV-159	MR233-17E/5821	2587 nt	OR843779	17.4682	424	orf1: ParB (pfam02195); orf2-par: RdRp2 (pfam00978, cd23179)
TC-Tombus-LV-160	MR233-17E/5835	2584 nt	OR843780	17.4981	318	orf1-par; orf2: RdRp3 (pfam00998, cd23179)
TC-Tombus-LV-161	MR233-17E/5870	2578 nt	OR843781	18.922	344	orf1: RdRp3 (pfam00998, cd23206); orf2
TC-Tombus-LV-162	MR233-17E/5910	4587 nt	OR843782	22.5701	740	orf1; orf2: RdRp3 (pfam00998, cd23242); orf3: CP (pfam00729)
TC-Tombus-LV-163	MR233-17E/5953	2555 nt	OR843783	13.319	240	orf1-par: RdRp3 (pfam00998, cd23242); orf2; orf3
TC-Tombus-LV-164	MR233-17E/6073	2526 nt	OR843784	10.3234	184	orf1-par: RdRp3 (pfam00998, cd23179); orf2
TC-Tombus-LV-165	MR233-17E/6207	4168 nt	OR843785	29.0054	838	orf1: P33 (pfam0855); orf2: RdRp3 (pfam00998, cd23239); orf3: CP (pfam00729)
TC-Tombus-LV-166	MR233-17E/6222	2495 nt	OR843786	10.2834	188	orf1-par: RdRp3 (pfam00998, cd23239); orf2: CP (pfam00729)
TC-Tombus-LV-167	MR233-17E/6255	4258 nt	OR843787	53.7586	1644	orf1; orf2: RdRp3 (pfam00998, cd23242); orf3; orf4: CP (pfam00729)
TC-Tombus-LV-168	MR233-17E/6454	2449 nt	OR843788	559.307	9788	orf1-par; orf2: RdRp3 (pfam00998, cd23179)
TC-Tombus-LV-169	MR233-17D/7860	3492 nt	OR843789	37.695	946	orf1: P33 (pfam08500); orf2: RdRp3 (pfam00998, cd23206); orf3
TC-Tombus-LV-170	MR233-17E/6769	2386 nt	OR843790	13.0457	218	orf1-par: P33 (pfam0855); orf2: RdRp3 (pfam00998, cd23242); orf3-par: CP (pfam00729)
TC-Tombus-LV-171	MR233-17E/6842	4014 nt	OR843791	211.881	6060	orf1-par: Panicovirus-RdRp (cd23238); orf2: RdRp3 (pfam00998, cd23242); orf3: CP (pfam00729); orf4
TC-Tombus-LV-172	MR233-17E/6924	2893 nt	OR843792	68815.7	1428396	orf1; orf2: RdRp (cd23179)
TC-Tombus-LV-173	MR233-17E/6927	2356 nt	OR843793	24.927	414	orf1; orf2: RdRp3 (pfam00998; cd23242)
TC-Tombus-LV-174	MR233-17E/6963	2348 nt	OR843794	18.7159	320	orf1-par: RdRp3 (pfam00998, cd23239); orf2: PLA2 (cd04706), CP (pfam00729)
TC-Tombus-LV-175	MR233-17E/6977	3888 nt	OR843795	25.1337	690	orf1; orf2: RdRp3 (pfam00998, cd23179)
TC-Tombus-LV-176	MR233-17E/6986	2344 nt	OR843796	11.9646	198	orf1-par: RdRp3 (pfam00998, cd23242); orf2; orf3: CP (pfam00729)
TC-Tombus-LV-177	MR233-17E/7001	2861 nt	OR843797	87.0003	1760	orf1-par; orf2: RdRp3 (pfam00998, cd23179)
TC-Tombus-LV-178	MR233-17E/7046	3264 nt	OR843798	13.8505	314	orf1; orf2: RdRp3 (pfam00998, cd23242); orf3
TC-Tombus-LV-179	MR233-17E/4881	4254 nt	OR843799	123.851	3728	orf1; orf2: RdRp3 (pfam00998, cd23242); orf3: CP (pfam00729); orf4
TC-Tombus-LV-180	MR233-17E/7345	2286 nt	OR843800	13.0542	212	suppression of UAG and UAA codons; orf1-par; orf2: RdRp3 (pfam00998, cd23206); orf3-par: CP (pfam00729)
TC-Tombus-LV-181	MR233-17E/7357	3555 nt	OR843801	996.337	25298	suppression of UAG and UAA codons: orf1-par; orf2: RdRp3 (pfam00998, cd23206); orf3: CP (pfam00729)
TC-Tombus-LV-182	MR233-17E/7451	2267 nt	OR843802	10.7186	176	orf1-par: RdRp3 (pfam00998, cd23206); orf2-par: CP (pfam00729)
TC-Tombus-LV-183	MR233-17E/7474	2264 nt	OR843803	12.2597	196	orf1-par; orf2: RdRp3 (pfam00998, cd23179); orf3-par: CP (pfam11729)
TC-Tombus-LV-184	MR233-17D/2652	2841 nt	OR843804	30.5945	626	orf1-par; orf2: RdRp3 (pfam00998, cd23242); orf3
TC-Tombus-LV-185	MR233-17E/7986	2186 nt	OR843805	14.57	226	orf1-par: P33 (pfam08500); orf2: RdRp3 (pfam00998, cd23240)
TC-Tombus-LV-186	MR233-17E/8076	2174 nt	OR843806	16.1812	248	orf1-par: RdRp3 (pfam00998, cd23242); orf2: CP (pfam00729)
TC-Tombus-LV-187	MR233-17E/8198	2156 nt	OR843807	28.9133	438	orf1-par; orf2: RdRp3 (pfam00998, cd23242); orf3-par
TC-Tombus-LV-188	MR233-17E/8280	2146 nt	OR843808	25.2307	382	orf1: P33 (pfam08500); orf2-par: RdRp3 (pfam00998, cd23206)
TC-Tombus-LV-189	MR233-17E/8334	2138 nt	OR843809	23.703	360	suppression of UAG and UAA codons; orf-par: CP (pfam00729)
TC-Tombus-LV-190	MR233-17E/8438	2122 nt	OR843810	12.6159	194	suppression of UAG and UAA codons; orf1-par: RdRp3 (pfam00998, cd23244); orf2: CP (pfam00729)
TC-Tombus-LV-191	MR233-17E/8485	2863 nt	OR843811	25.8893	526	orf1-par; orf2: RdRp3 (pfam00998, cd23240); orf3
TC-Tombus-LV-192	MR233-17E/8559	2108 nt	OR843812	9,14469	136	orf1-par; orf2: CP (pfam00729)
TC-Tombus-LV-193	MR233-17E/8570	2106 nt	OR843813	14.916	226	orf1-par: RdRp3 (pfam00998, cd23206); orf2
TC-Tombus-LV-194	MR233-17E/8636	3821 nt	OR843814	17.9142	484	orf1; orf2: RdRp3 (pfam00998, cd23206); orf3: CP (pfam00729)
TC-Tombus-LV-195	MR233-17E/8673	2093 nt	OR843815	197.51	2960	suppression of UAG and UAA codons: orf1-par; orf2-par: RdRp3 (pfam00998, cd23206)
TC-Tombus-LV-196	MR233-17E/8761	2082 nt	OR843816	11.5672	168	orf1-par: RdRp3 (pfam00998, cd23206); orf2
TC-Tombus-LV-197	MR233-17E/4162	3095 nt	OR843817	1940.64	42732	orf1-par; orf2: RdRp2 (pfam00978, cd01699)
TC-Tombus-LV-198	MR233-17/5670	5024 nt	OR843818	149.401	5366	orf1-par; orf2: RdRp1 (pfam00680, cd23173); orf3: CP (pfam00729)
TC-Tombus-LV-199	MR233-17E/1826	4982 nt	OR843819	353.77	12594	orf1-par; orf2: RdRp1 (pfam00680, cd23173); orf3: CP (pfam00729)
TC-Tombus-LV-200	MR233-17E/10090	1938 nt	OR843820	23.4541	328	orf1: P33 (pfam08500); orf2-par: RdRp3 (pfam00998, cd23244)
TC-Tombus-LV-201	MR233-17D/5671	2911 nt	OR843821	96.5946	2002	orf1-par; orf2: RdRp1 (pfam00680; cd23173); orf3: CP (pfam00729)
TC-Tombus-LV-202	MR233-17/12072	1967 nt	OR843822	30.303	432	orf1-par; orf2: CP (pfam00729)

TC-Tombus-LV-203	MR233-17E/9681	1981 nt	OR843823	43.3049	616	orf1: Panicovirus-RdRp (cd23238); orf2-par: RdRp3 (pfam00998, cd23242)
TC-Tombus-LV-204	MR233-17E/9685	1981 nt	OR843824	9.73195	140	orf1-par: RdRp3 (pfam00998, cd23242); orf2: CP (pfam00729)
TC-Tombus-LV-205	MR233-17E/9781	2552 nt	OR843825	24.8734	454	orf1-par: P33 (pfam08500); orf2: RdRp3 (pfam00998, cd23242); orf3-par
TC-Tombus-LV-206	MR233-17E/5426	2688 nt	OR843826	84.292	1622	orf1-par; orf2: RdRp3 (pfam00998, cd23242)
TC-Tombus-LV-207	MR233-17E/10491	2121 nt	OR843827	16.8029	254	orf1-par: RdRp3 (pfam00998, cd23236); orf2: CP (pfam00729)
TC-Tombus-LV-208	MR233-17D/10691	2288 nt	OR843828	49.3239	802	orf1-par: RdRp3 (pfam00998, cd23236); orf2: CP (pfam00729); orf3: P22 (pfam03558)
TC-Tombus-LV-209	MR233-17E/13549	3791 nt	OR843829	252.348	6806	orf1-par: P33 (pfam08500); orf2: RdRp3 (pfam00998, cd23236); orf3: CP (pfam00729); orf4: P22 (pfam03558)
TC-Tombus-LV-210	MR233-17/17622	3545 nt	OR843830	77.4666	1970	orf1-par: RdRp3 (pfam00998, cd23236); orf2: CP (pfam00729); orf3: P22 (pfam03558)
TC-Tombus-LV-211	MR233-17E/1027	6472 nt	OR843831	56.5596	2624	orf1-par; orf2: RdRp (cd01699)
TC-Tombus-LV-212	MR233-17E/814	6883 nt	OR843832	22.2024	1102	orf1: RdRp (cd01699)
TC-Tombus-LV-213	MR233-17E/1476	5199 nt	OR843833	278.516	10312	orf1; orf2: RdRp3 (pfam00998, cd23179); orf3; orf4
TC-Tombus-LV-214	MR233-17E/1336	5490 nt	OR843834	17.7424	696	orf1; orf2-par: noda-like RdRp (cd23173)
TC-Tombus-LV-215	MR233-17D/76372	1906 nt	OR843835	19.4963	264	orf1-par: RdRp3 (pfam00998, cd23236); orf2: CP (pfam00729); orf3-par: P22 (pfam03558)
TC-Tombus-LV-216	MR233-17E/10743	2782 nt	OR843836	9.22466	178	orf1-par: RdRp3 (pfam00998, cd23242); orf2: CP (pfam00729)
TC-Tombus-LV-217	MR233-17E/10904	1867 nt	OR843837	15.2989	202	orf1-par: RdRp3 (pfam00998, cd23242); orf2
TC-Tombus-LV-218	MR233-17E/10985	2384 nt	OR843838	37.5692	630	orf1: P33 (pfam08500); orf2: RdRp (cd23240); orf3-par: CP (pfam00729)
TC-Tombus-LV-219	MR233-17E/11065	1852 nt	OR843839	20.0248	260	orf1-par; orf2-par: RdRp3 (pfam00998, cd23206)
TC-Tombus-LV-220	MR233-17E/12068	1774 nt	OR843840	9.11894	114	orf1-par; orf2-par: RdRp3 (pfam00998, cd23179)
TC-Tombus-LV-221	MR233-17E/10954	2846 nt	OR843841	76.9807	1564	orf1-par: RdRp3 (pfam00998, cd23179); orf2
TC-Tombus-LV-222	MR233-17D/2000	1359 nt	OR843842	237.829	2314	orf1: CP (pfam00729); orf2
TC-Tombus-LV-223	MR233-17E/12101	1771 nt	OR843843	14.5726	182	orf: CP (pfam00729)
TC-Tombus-LV-224	MR233-17E/12241	1762 nt	OR843844	6.82804	86	orf1-par; orf2-par: RdRp (cd23206)
TC-Tombus-LV-225	MR233-17E/12295	1758 nt	OR843845	6.7025	86	orf-par: RdRp3 (pfam00998, cd23179)
TC-Tombus-LV-226	MR233-17E/12345	1754 nt	OR843846	14.0296	176	orf1; orf2-par: RdRp3 (pfam00998, cd23206)
TC-Tombus-LV-227	MR233-17E/12390	1751 nt	OR843847	11.9754	156	orf1-par: RdRp3 (pfam00998, cd23206); orf2-par: CP (pfam00729)
TC-Tombus-LV-228	MR233-17E/12415	1749 nt	OR843848	22.1727	278	orf1-par: RdRp3 (pfam00998, cd23244); orf2: CP (pfam00729)
TC-Tombus-LV-229	MR233-17E/12934	1713 nt	OR843849	5.95914	70	orf1-par; orf2-par: RdRp3 (pfam00998, cd23206)
TC-Tombus-LV-230	MR233-17E/12986	1914 nt	OR843850	22.3558	304	orf1; orf2-par: RdRp3 (pfam00998, cd23206)
TC-Tombus-LV-231	MR233-17E/13018	1707 nt	OR843851	9.42121	114	orf1-par: RdRp3 (pfam00998, cd23206); orf2-par: CP (pfam00729)
TC-Tombus-LV-232	MR233-17E/13081	1703 nt	OR843852	9.35643	112	orf1-par; orf2-par: RdRp3 (pfam00998, cd23240)
TC-Tombus-LV-233	MR233-17E/13174	1697 nt	OR843853	956099	118	orf1-par; orf2-par: RdRp3 (pfam00998, cd23179)
TC-Tombus-LV-234	MR233-17E/13189	1696 nt	OR843854	9.898	122	suppression of UAG and UAA codons; orf1-par; orf2-par: RdRp (cd23206)
TC-Tombus-LV-235	MR233-17E/8093	4091 nt	OR843855	101.536	2948	orf1-par; orf2: RdRp3 (pfam00998, cd23206); orf3: CP (pfam00729)
TC-Tombus-LV-236	MR233-17E/13295	1689 nt	OR843856	10.2872	126	orf1-par: RdRp3 (pfam00998, cd23237); orf2-par: CP (pfam00729)
TC-Tombus-LV-237	MR233-17E/13968	2961 nt	OR843857	10.4583	222	orf1; orf2: RdRp3 (pfam00998, cd23242); orf3-par
TC-Tombus-LV-238	MR233-17/13984	1782 nt	OR843858	12.5684	156	orf1-par: P33 (pfam08500); orf2-par: RdRp3 (pfam00998, cd23240)
TC-Tombus-LV-239	MR233-17E/14041	1642 nt	OR843859	8.08709	94	orf-par: RdRp3 (pfam00998, cd23179)
TC-Tombus-LV-240	MR233-17E/14258	3312 nt	OR843860	131.956	3144	orf1-par: RdRp3 (pfam00998, cd23179); orf2: CP (pfam00729)
TC-Tombus-LV-241	MR233-17E/14259	2605 nt	OR843861	96.6614	1812	orf-par: RdRp3 (pfam00998, cd23179)
TC-Tombus-LV-242	MR233-17E/14302	3099 nt	OR843862	6.62343	144	orf1-par; orf2: RdRp3 (pfam00998, cd23242); orf3-par: CP (pfam00729)
TC-Tombus-LV-243	MR233-17E/14504	1614 nt	OR843863	5.47026	60	suppression of UAG and UAA codons; orf1-par: RdRp3 (pfam00998, cd23206); orf2-par: CP (pfam00729)
TC-Tombus-LV-244	MR233-17E/15069	1743 nt	OR843864	8.69421	82	orf1-par: RdRp3 (pfam00998, cd23239); orf2-par: PLA2 (cd00618), CP (pfam00729)
TC-Tombus-LV-245	MR233-17E/31227	3022 nt	OR843865	24.6625	526	orf1-par: P33 (pfam08500); orf2: RdRp3 (pfam00998, cd23244); orf3-par
TC-Tombus-LV-246	MR233-17E/15198	2898 nt	OR843866	14.1825	284	orf1-par: P33 (pfam08500); orf2: RdRp3 (pfam00998, cd23206); orf3: CP (pfam00729)
TC-Tombus-LV-247	MR233-17E/15460	1564 nt	OR843867	6.29923	70	orf1-par; orf2-par: CBPV-RdRp (cd23174)
TC-Tombus-LV-248	MR233-17E/15832	1546 nt	OR843868	7.94955	86	orf1-par; orf2-par: RdRp3 (pfam00998, cd23239)
TC-Tombus-LV-249	MR233-17E/15888	1543 nt	OR843869	20.6086	226	orf-par: CP (pfam00729)
TC-Tombus-LV-250	MR233-17E/16172	2874 nt	OR843870	24.222	492	orf1-par; orf2: RdRp3 (pfam00998, cd23206); orf3: CP (pfam00729)
TC-Tombus-LV-251	MR233-17E/16227	3259 nt	OR843871	36.4851	852	orf1-par: RdRp3 (pfam00998, cd23206); orf2; orf3: CP (pfam00729)
TC-Tombus-LV-252	MR233-17E/16281	3864 nt	OR843872	120.952	3330	orf1: P33 (pfam08500); orf2: RdRp3 (pfam00998, cd23244); orf3: CP (pfam00729)
TC-Tombus-LV-253	MR233-17E/16474	1513 nt	OR843873	14.115	152	orf-par: CP (pfam00729)

TC-Tombus-LV-254	MR233-17D/11999	2599 nt	OR843874	85.5278	1620	orf1-par: RdRp3 (pfam00998, cd23244); orf2: CP (pfam00729)
TC-Tombus-LV-255	MR233-17E/16842	1497 nt	OR843875	9.42151	98	orf1-par: RdRp3 (pfam00998, cd23206); orf2-par
TC-Tombus-LV-256	MR233-17E/17057	1488 nt	OR843876	465.605	4952	orf: CP (pfam11729)
TC-Tombus-LV-257	MR233-17E/17101	1487 nt	OR843877	7.01479	74	orf1; orf2: RdRp3 (pfam00998, cd23179)
TC-Tombus-LV-258	MR233-17E/17288	1479 nt	OR843878	12.3867	130	orf1-par: RdRp3 (pfam00998, cd23206); orf2-par
TC-Tombus-LV-259	MR233-17E/17568	1467 nt	OR843879	24.5106	258	orf-par: CP (pfam00729)
TC-Tombus-LV-260	MR233-17E/17828	1457 nt	OR843880	15.0384	154	orf1-par: RdRp3 (pfam00998, cd23206); orf2-par
TC-Tombus-LV-261	MR233-17E/18091	1446 nt	OR843881	143.003	1486	suppression of UAG and UAA codons; orf: CP (pfam00729)
TC-Tombus-LV-262	MR233-17E/18123	1445 nt	OR843882	8.48997	86	orf1-par: RdRp3 (pfam00998, cd23242); orf2
TC-Tombus-LV-263	MR233-17/18241	1440 nt	OR843883	9.91736	110	orf1-par; orf2-par: RdRp3 (pfam00998, cd23241)
TC-Tombus-LV-264	MR233-17E/18330	1977 nt	OR843884	36.9555	524	orf1-par: RdRp3 (pfam00998, cd23242); orf2: CP (pfam00729)
TC-Tombus-LV-265	MR233-17E/18332	1436 nt	OR843885	15.9854	162	orf-par: CP (pfam00729)
TC-Tombus-LV-266	MR233-17E/18411	1433 nt	OR843886	7.01256	72	orf1-par; orf2-par: RdRp3 (pfam00998, cd23179)
TC-Tombus-LV-267	MR233-17E/18776	1419 nt	OR843887	18.3805	190	orf1; orf2-par: RdRp3 (pfam00998, cd23206)
TC-Tombus-LV-268	MR233-17E/18779	1627 nt	OR843888	13.4831	156	orf1-par: RdRp3 (pfam00998, cd23243); orf2-par: CP (pfam00729)
TC-Tombus-LV-269	MR233-17E/18874	1416 nt	OR843889	4,84534	48	orf1-par: RdRp3 (pfam00998, cd23242); orf2
TC-Tombus-LV-270	MR233-17E/19161	1634 nt	OR843890	4.85129	56	orf1-par: Panicovirus-RdRp (cd23238); orf2-par: RdRp3 (pfam00998, cd23206)
TC-Tombus-LV-271	MR233-17E/19317	1719 nt	OR843891	13.6353	164	orf1-par; orf2-par: RdRp3 (pfam00998, cd23206)
TC-Tombus-LV-272	MR233-17E/19417	1395 nt	OR843892	150.365	1504	orf: CP (pfam00729)
TC-Tombus-LV-273	MR233-17E/19604	1388 nt	OR843893	6.43156	64	orf-par: RdRp3 (pfam00998, cd23242)
TC-Tombus-LV-274	MR233-17D/205827	1455 nt	OR843894	8.00275	84	orf1-par: RdRp3 (pfam00998, cd23242); orf2-par
TC-Tombus-LV-275	MR233-17E/19972	1375 nt	OR843895	22.9418	230	orf: CP (pfam00729)
TC-Tombus-LV-276	MR233-17E/20039	1373 nt	OR843896	6.47706	62	orf-par: RdRp3 (pfam00998, cd23179)
TC-Tombus-LV-277	MR233-17E/20409	1360 nt	OR843897	188.612	2734	orf: CP (pfam00729)
TC-Tombus-LV-278	MR233-17E/20792	1347 nt	OR843898	18.9272	182	orf-par: RdRp (cd23206)
TC-Tombus-LV-279	MR233-17E/20873	1345 nt	OR843899	6.57621	62	orf-par: RdRp3 (pfam00998, cd23179)
TC-Tombus-LV-280	MR233-17E20967	1661 nt	OR843900	84.9825	1002	orf1-par: RdRp3 (pfam00998, cd23242); orf2-par
TC-Tombus-LV-281	MR233-17E/354003	2325 nt	OR843901	93.1996	1552	orf1-par: RdRp3 (pfam00998, cd23236); orf2: CP (pfam00729); orf3-par: P22 (pfam03558)
TC-Tombus-LV-282	MR233-17E/21417	1329 nt	OR843902	3.80286	36	suppression of UAG and UAA codons; orf1-par; orf2-par: CP (pfam00729)
TC-Tombus-LV-283	MR233-17E/21461	1720 nt	OR843903	93.2884	1156	suppression of UAG and UAA codons; orf1-par: RdRp (cd23243); orf2: CP (pfam00729); orf3-par
TC-Tombus-LV-284	MR233-17E/21823	1317 nt	OR843904	5.78664	54	orf-par: RdRp2 (pfam00978, cd01699)
TC-Tombus-LV-285	MR233-17E/102450	5410 nt	OR843905	32.9854	1270	orf1; orf2: RdRp3 (pfam00998, cd23206); orf3: CP (pfam00729)
TC-Tombus-LV-286	MR233-17D/42383	4804 nt	OR843906	22.8494	794	orf1; orf2; orf3: RdRp3 (pfam00998, cd23206); orf4-par: CP (pfam00729)
TC-Tombus-LV-287	MR233-17E/21850	1316 nt	OR843907	7.51414	74	orf1-par; orf2-par
TC-Tombus-LV-288	MR233-17E/22011	1540 nt	OR843908	4.32727	48	orf1-par: RdRp3 (pfam00998, cd23206); orf2-par: CP (pfam00729)
TC-Tombus-LV-289	MR233-17E/22302	1303 nt	OR843909	19.0184	182	orf1-par: RdRp3 (pfam00998, cd23206); orf2-par
TC-Tombus-LV-290	MR233-17E/22533	1881 nt	OR843910	6.58745	88	orf1-par: RdRp3 (pfam00998, cd23179); orf2-par
TC-Tombus-LV-291	MR233-17E/22562	1295 nt	OR843911	9.04402	84	orf1-par: CP (pfam00729); orf2-par
TC-Tombus-LV-292	MR233-17E/23274	1276 nt	OR843912	10.5807	98	orf: RdRp3 (pfam00998, cd23206)
TC-Tombus-LV-293	MR233-17E/23559	2388 nt	OR843913	7.2701	126	orf1-par: RdRp3 (pfam00998, cd23206); orf2: CP (pfam00729)
TC-Tombus-LV-294	MR233-17E/40814	2885 nt	OR843914	224.069	4616	orf1-par: P33 (pfam08500); orf2: RdRp3 (pfam00998, cd23239); orf3
TC-Tombus-LV-295	MR233-17E/24382	2076 nt	OR843915	7.35934	106	orf1: RdRp3 (pfam00998, cd23242); orf2-par: CP (pfam00729)
TC-Tombus-LV-296	MR233-17E/24767	1237 nt	OR843916	5.32579	44	orf1-par; orf2-par: RdRp3 (pfam00998, cd23242)
TC-Tombus-LV-297	MR233-17E/24947	2522 nt	OR843917	7.17962	128	orf1-par: RdRp3 (pfam00998, cd23206); orf2: CP (pfam00729)
TC-Tombus-LV-298	MR233-17E/25089	1229 nt	OR843918	7.72742	68	orf1-par: RdRp3 (pfam00998, cd23242); orf2-par
TC-Tombus-LV-299	MR233-17E/25566	1218 nt	OR843919	17.3259	152	orf-par: RdRp3 (pfam00998, cd23179)
TC-Tombus-LV-300	MR233-17E/26412	1199 nt	OR843920	6.52794	56	orf1-par; orf2-par: CBPV-RdRp (cd23174)
TC-Tombus-LV-301	MR233-17E/27668	1172 nt	OR843921	8.79352	72	orf1-par; orf2-par: RdRp3 (pfam00998, cd23179)
TC-Tombus-LV-302	MR233-17E/27959	1166 nt	OR843922	9.49657	80	orf-par: RdRp3 (pfam00998, cd23206)
TC-Tombus-LV-303	MR233-17E/28208	1388 nt	OR843923	4.79683	48	orf-par: Peptidase A21 (pfam03566)
TC-Tombus-LV-304	MR233-17E/28796	1149 nt	OR843924	5.34813	44	orf-par: RdRp2 (pfam00978, cd23174)
TC-Tombus-LV-305	MR233-17E/28925	1418 nt	OR843925	87.3611	886	orf-par: CP (pfam00729); orf2-par
TC-Tombus-LV-306	MR233-17E/38212	1003 nt	OR843926	7.15354	50	orf1-par: RdRp3 (pfam00998, cd23236); orf2-par
TC-Tombus-LV-307	MR233-17E/29286	1139 nt	OR843927	4.81036	42	orf-par: CP (pfam00729)
TC-Tombus-LV-308	MR233-17E/29532	1134 nt	OR843928	10.813	90	suppression of UAG and UAA codons; orf1-par: RdRp3 (pfam00998, cd23242); orf2-par: CP (pfam00729)

TC-Tombus-LV-309	MR233-17E/29541	1134 nt	OR843929	9,78836	78	orf-par: RdRp3 (pfam00998, cd23238)
TC-Tombus-LV-310	MR233-17E/29961	1126 nt	OR843930	14.4112	118	orf1-par: CP (pfam00729); orf2: P22 (pfam03558)
TC-Tombus-LV-311	MR233-17E/30235	1122 nt	OR843931	4.24866	34	orf1-par: RdRp3 (pfam00998, cd23179); orf2-par
TC-Tombus-LV-312	MR233-17E/30348	1447 nt	OR843932	52.9489	542	orf1; orf2-par: RdRp3 (pfam00998, cd23241)
TC-Tombus-LV-313	MR233-17E/30558	1116 nt	OR843933	6.43548	52	orf1-par: RdRp3 (pfam00998, cd23239); orf2-par: CP (pfam00729)
TC-Tombus-LV-314	MR233-17E/30956	1109 nt	OR843934	5.74031	46	orf1-par; orf2-par: RdRp3 (pfam00998, cd23240)
TC-Tombus-LV-315	MR233-17E/31886	2840 nt	OR843935	71.218	1444	orf1-par; orf2: RdRp3 (pfam00998, cd23240); orf3: CP (pfam00729)
TC-Tombus-LV-316	MR233-17E/32931	1167 nt	OR843936	78.3779	660	orf: P33 (pfam08500)
TC-Tombus-LV-317	MR233-17E/33228	1072 nt	OR843937	6.50933	52	orf-par: RdRp3 (pfam00998, cd23242)
TC-Tombus-LV-318	MR233-17E/33268	1071 nt	OR843938	8.23623	62	orf-par: RdRp3 (pfam00998, cd01699)
TC-Tombus-LV-319	MR233-17E/33346	1480 nt	OR843939	7.38188	74	orf1-par: RdRp3 (pfam00998, cd23237); orf2-par: CP (pfam00729)
TC-Tombus-LV-320	MR233-17E/33411	1069 nt	OR843940	9.30964	68	orf-par: CP (pfam00729)
TC-Tombus-LV-321	MR233-17E/33419	1676 nt	OR843941	7.06416	84	orf1-par; orf2-par: RdRp3 (pfam00998, cd23206)
TC-Tombus-LV-322	MR233-17E/34098	1059 nt	OR843942	3.60057	26	orf-par: RdRp3 (pfam00998, cd23206)
TC-Tombus-LV-323	MR233-17E/34177	1057 nt	OR843943	19.35	144	orf1-par; orf2-par: RdRp3 (pfam00998, cd23234)
TC-Tombus-LV-324	MR233-17E/34903	1047 nt	OR843944	6.0936	46	orf-par: RdRp3 (pfam00998, cd23179)
TC-Tombus-LV-325	MR233-17E/35444	1039 nt	OR843945	13.0308	100	orf-par: RdRp3 (pfam00998, cd23234)
TC-Tombus-LV-326	MR233-17/35596	1136 nt	OR843946	9.41109	74	orf-par: RdRp3 (pfam00998, cd23206)
TC-Tombus-LV-327	MR233-17E/35728	1035 nt	OR843947	11.4725	86	orf-par: RdRp3 (pfam00998, cd23241)

	TC-Tombus-LV-328	MR233-17E/36503	1025 nt	OR843948	11.3688	84	orf1-par: P33 (pfam08500); orf2-par: RdRp (cd23236)
	TC-Tombus-LV-329	MR233-17/37009	2471 nt	OR843949	102.212	1794	orf1: RdRp3 (pfam00998, cd23242); orf2: CP (pfam00729)
	TC-Tombus-LV-330	MR233-17E/37080	1018 nt	OR843950	3.2554	24	orf-par: CP (pfam00729)
	TC-Tombus-LV-331	MR233-17E/37903	1007 nt	OR843951	10.2006	72	orf1-par; orf2-par: RdRp3 (pfam00998, cd23241)
	TC-Tombus-LV-332	MR233-17E/557	8184 nt	OR843952	40.357	2352	orf1; orf2; orf3: CBPV-RdRp (cd23174)
	TC-Tombus-LV-333	MR233-17E/488	8546 nt	OR843953	14.6201	886	orf1; orf2; orf3: RdRp (cd01699)
	TC-Tombus-LV-334	MR233-17E/48080	1049 nt	OR843954	9.82936	74	orf-par: CP (pfam00729)
	TC-Tombus-LV-335	MR233-17D/127838	1301 nt	OR843955	6.38432	58	orf-par: PepA21 (pfam03566)
Virgaviridae	Havel cucumber green mottle mosaic virus 1	MR644-18D/6593	6487 nt	OR843956	196.244	9120	orf1-2: VMethylTr (pfam01660), Helicase 1 (pfam01443), RdRp2 (pfam00978, cd23251); orf3: MP (pfam01107); orf4: CP (pfam00721)
	Havel cucumber green mottle mosaic virus 2	MR644-18D/7065	4814 nt	OR843957	62.9887	2242	orf1-2-par: Helicase 1 (pfam01443), RdRp2 (pfam00978, cd23251), orf3: MP (pfam01107), orf4: CP (pfam00721)
	Havel cucumber green mottle mosaic virus 3	MR644-18E/367	6394 nt	OR843958	74.2477	3404	orf1-2: VMethylTr (pfam01660), Helicase 1 (pfam01443), RdRp2 (pfam00978, cd23251); orf3: MP (pfam01107); orf4: CP (pfam00721)
	Havel pepper mild mottle virus 1	MR644-18E/141	5723 nt	OR843959	67.7239	2794	orf1-2: VMethylTr (pfam01660), Helicase 1 (pfam01443), RdRp2 (pfam00978, cd01699); orf3: MP (pfam0110)
	Havel pepper mild mottle virus 2	MR644-18D/4643	3101 nt	OR843960	21.2828	466	orf1-2-par: Helicase 1 (pfam01443), RdRp2 (pfam00978, cd23251), orf3-par: MP (pfam01107)
	Havel pepper mild mottle virus 3	MR644-18D/5625	1853 nt	OR843961	26.3357	356	orf1-2-par: Helicase 1 (pfam01443), RdRp2 (pfam00978, cd23251)
	Havel tobacco mild green mosaic virus	MR644-18/1455	2389 nt	OR843962	7.65365	144	orf1-2-par: Helicase 1 (pfam01443), RdRp2 (pfam00978, cd23251)
	Havel tobacco mosaic virus 1	MR644-18E/1480	1954 nt	OR843963	11.1351	158	orf1-par: Helicase 1 (pfam01443)
	Havel tobacco mosaic virus 2	MR644-18E/3430	2586 nt	OR843964	11.5557	216	orf1-2-par: Helicase 1 (pfam01443), RdRp2 (pfam00978, cd23252); orf3-par: MP (pfam01107)
	Havel tobacco mosaic virus 3	MR644-18/10659	1083 nt	OR843965	14.8098	114	orf2-par: RdRp2 (pfam00978, cd23252); orf3-par: MP (pfam01107)
	Havel turnip vein-clearing virus 1	MR644-18E/2831	1332 nt	OR843966	6.81907	66	orf3-par: MP (pfam01107); orf4: CP (pfam00721)
	Havel turnip vein-clearing virus 2	MR644-18D/30380	998 nt	OR843967	5.56112	44	orf1-par: VMethylTr (pfam01660)
	Teltow Canal African eggplant-associated virus	MR233-17E/1297	5557 nt	OR843968	28.837	1644	orf1-2-par: VMethylTr (pfam01660), Helicase 1 (pfam01443), RdRp2 (pfam00978, cd23251); orf3: MP (pfam01107); orf4: CP (pfam00721)
	Teltow Canal chili pepper mild mottle virus	MR233-17E/976	6303 nt	OR843969	36.5905	570194	orf1-2: VMethylTr (pfam01660), Helicase 1 (pfam01443), RdRp2 (pfam00978, cd23251); orf3: MP (pfam01107); orf4: CP (pfam00721)
	Teltow Canal cucumber green mottle mosaic virus 1	MR233-17E/3327	6478 nt	OR843970	12382.4	420216	orf1-2: VMethylTr (pfam01660), Helicase 1 (pfam01443), RdRp2 (pfam00978, cd23251); orf3: MP (pfam01107); orf4: CP (pfam00721)
	Teltow Canal cucumber green mottle mosaic virus 2	MR233-17E/1485	6465 nt	OR843971	9145.23	58	orf1-2: VMethylTr (pfam01660), Helicase 1 (pfam01443), RdRp2 (pfam00978, cd23251); orf3: MP (pfam01107); orf4: CP (pfam00721)
	Teltow Canal Odontoglossum ringspot virus	MR233-17E/19797	1381 nt	OR843972	5.95655	13782	orf2-par: RdRp2 (pfam00978, cd23252)
	Teltow Canal paprika mild mottle virus	MR233-17E/920	6506 nt	OR843973	296.356	240544	orf1-2: VMethylTr (pfam01660), Helicase 1 (pfam01443), RdRp2 (pfam00978, cd23251); orf3: MP (pfam01107); orf4: CP (pfam00721)
	Teltow Canal pepper mild mottle virus	MR233-17E/1050	6342 nt	OR843974	5325.65	456	orf1-2: VMethylTr (pfam01660), Helicase 1 (pfam01443), RdRp2 (pfam00978, cd23251); orf3: MP (pfam01107); orf4: CP (pfam00721)
	Teltow Canal Rehmannia mosaic virus 1	MR233-17E/21037	1339 nt	OR843975	47.2651	534	orf1-par: Helicase 1 (pfam01443)
	Teltow Canal Rehmannia mosaic virus 2	MR233-17E/29780	1129 nt	OR843976	63.6492	430	orf3: MP (pfam01107); orf4-par: CP (pfam00721)
	Teltow Canal ribgrass mosaic virus 1	MR233-17E/20436	1359 nt	OR843977	43.8102	420	orf1-par: VMethylTr (pfam01660)
	Teltow Canal ribgrass mosaic virus 2	MR233-17E/17110	1486 nt	OR843978	39.0834	816	orf1-par: Helicase 1 (pfam01443)
	Teltow Canal ribgrass mosaic virus 3	MR233-17E/21771	1975 nt	OR843979	57.679	21140	orf1-2-par: Helicase 1 (pfam01443), RdRp2 (pfam00978, cd23251)
	Teltow Canal tobacco mild green mosaic virus	MR233-17E/963	6332 nt	OR843980	468.094	860	orf1-2: VMethylTr (pfam01660), Helicase 1 (pfam01443), RdRp2 (pfam00978, cd23251); orf3: MP (pfam01107); orf4: CP (pfam00721)
	Teltow Canal tobacco mosaic virus 1	MR233-17E/27744	2229 nt	OR843981	53.5451	8498	orf1: VMethylTr (pfam01660), Helicase 1 (pfam01443)
	Teltow Canal tobacco mosaic virus 2	MR233-17E/14113	3662 nt	OR843982	322.641	10202	orf1-2-par: VMethylTr (pfam01660, Helicase 1 (pfam01443), RdRp2 (pfam00978, cd23251)
	Teltow Canal tomato brown rugose fruit virus	MR233-17E/2703	6364 nt	OR843983	223.728	36340	orf1-2: VMethylTr (pfam01660), Helicase 1 (pfam01443), RdRp2 (pfam00978, cd23251); orf3: MP (pfam01107); orf4: CP (pfam00721)
	Teltow Canal tomato mosaic virus	MR233-17E/2015	6337 nt	OR843984	803.157	800	orf1-2: VMethylTr (pfam01660), Helicase 1 (pfam01443), RdRp2 (pfam00978, cd23251); orf3: MP (pfam01107); orf4: CP (pfam00721)
	Teltow Canal tomato mottle mosaic virus	MR233-17E/2807	5250 nt	OR843985	21.1244	1158	orf1-2-par: VMethylTr (pfam01660), Helicase 1 (pfam01443), RdRp2 (pfam00978, cd23251); orf3: MP (pfam01107); orf4: CP (pfam00721)
	Teltow Canal turnip vein-clearing virus 1	MR233-17E/23953	3120 nt	OR843986	142.97	3186	orf1-2-par: Helicase 1 (pfam01443), RdRp2 (pfam00978, cd23251)
	Teltow Canal turnip vein-clearing virus 2	MR233-17E/25949	1209 nt	OR843987	64.1572	556	orf2-par: RdRp2 (pfam00978, cd23251)
	Teltow Canal turnip vein-clearing virus 3	MR233-17E/37541	1011 nt	OR843988	54.9862	402	orf1-par: VMethylTr (pfam01660)
